# Toxic Trace Element Concentration in Commercially Available Cigarettes in Poland, Europe

**DOI:** 10.3390/toxics13121088

**Published:** 2025-12-18

**Authors:** Małgorzata Ćwieląg-Drabek, Joanna Domagalska, Marta Buczkowska, Agata Piekut

**Affiliations:** 1 Department of Environmental Health, Faculty of Public Health in Bytom, Medical University of Silesia in Katowice, 18 Piekarska Street, 41-902 Bytom, Poland; mdrabek@sum.edu.pl (M.Ć.-D.); apiekut@sum.edu.pl (A.P.); 2 Department of Chronic Diseases and Civilization-Related Hazards, Faculty of Public Health in Bytom, Medical University of Silesia in Katowice, 18 Piekarska Street, 41-902 Bytom, Poland; mbuczkowska@sum.edu.pl

**Keywords:** heavy metals, toxic trace elements, tobacco, smoking, health risk, environmental exposure

## Abstract

(1) Background: Tobacco use constitutes a significant preventable cause of morbidity and mortality on a global scale. It contributes to cumulative exposure to carcinogenic and toxic heavy metals, including cadmium, lead, nickel, and copper. Collectively, these metals promote oxidative stress, multi-organ damage, and an increased risk of cancer, cardiovascular, respiratory, and renal diseases; (2) Methods: The research material comprised 119 tobacco samples. The concentrations of cadmium, lead, copper, and nickel in the samples were subsequently determined. A series of calculations were conducted in order to estimate the non-carcinogenic and carcinogenic health risks associated with the consumption of tobacco products under a variety of exposure scenarios; (3) Results: The concentrations of heavy metals in the tobacco samples ranged as follows, with mean values: Cd: 0.3–8.6 mg/kg (mean 1.0), Cu: 3.4–92.6 mg/kg (mean 12.3), Ni: 1.1–15.4 mg/kg (mean 3.4), and Pb: 0.2–1633.3 mg/kg (mean 46.5). A health risk assessment indicated that exposure through inhalation to cadmium Cd, Ni, and Pb, even in the minimal smoking scenario of one cigarette per day, consistently exceeded internationally established thresholds for carcinogenic risk; (4) Conclusions: The presence of high inter-brand variability and high-risk outliers underscores the necessity for enhanced regulation and monitoring of toxic metals in tobacco products.

## 1. Introduction

Tobacco use continues to be a major preventable cause of death worldwide, with consequences that extend beyond nicotine addiction and exposure to carcinogens [1,2]. According to data from the World Health Organization (WHO), tobacco consumption is responsible for more than 7 million deaths worldwide each year, of which more than 1.6 million are attributable to passive exposure to tobacco smoke [3]. Evidence indicates that smoking remains a substantial risk factor for the development of non-communicable diseases, including cancer, cardiovascular and respiratory diseases, and chronic kidney disease [1,2,4]. The WHO emphasises that a reduction in tobacco consumption is one of the most cost-effective preventive measures in public health [3,5].

Historically, the focus of toxicological risk assessment related to tobacco use has been on the analysis of organic compounds, including tobacco-specific nitrosamines (TSNAs) and polycyclic aromatic hydrocarbons (PAHs) [6,7]. However, inorganic persistent elements, particularly heavy metals (HMs), are an equally important and irreversible component of the toxicological burden on the body [8,9].

The presence of cadmium (Cd) or lead (Pb) in raw tobacco has been determined not to be a result of accidental contamination. However, it has been demonstrated that this phenomenon is a consequence of Nicotiana tabacum’s capacity to hyperaccumulate elements from soils that have been contaminated by human activity, including phosphate fertilisers [10]. As with nickel (Ni) and copper (Cu), a similar accumulation pathway has been described. These elements enter the plant primarily through soils that have been impacted by industrial activities and fertiliser-derived inputs [10]. Consequently, tobacco leaves are a concentrated source of environmental toxins, which are released into tobacco aerosol during the combustion process [11]. Most smokers tend to choose one preferred cigarette brand, which they consume consistently over many years. This behavior leads to chronic exposure to the specific levels of heavy metals present in that brand, reinforcing the cumulative toxic effect.

In contrast to the degradation of organic compounds, the combustion of a cigarette results in the transformation of heavy metals into fine, highly bioavailable aerosol particles. These particles have the capacity for deep penetration into the respiratory tract and deposition in the alveoli [12,13]. Due to their protracted biological half-life (e.g., 10–30 years for Cd in the kidneys), there is gradual, chronic bioaccumulation [14]. Consequently, exposure to tobacco smoke results in permanent, cumulative heavy metal intoxication, independent of the metabolism of other xenobiotics [12,13,14].

It is evident that heavy metals such as Cd, Pb, and As are toxic, even at low levels of exposure. These toxins have the capacity to affect multiple organs [12,13,14,15]. Cadmium, classified by the International Agency for Research on Cancer (IARC) as a Group 1 carcinogen, has been associated with the development of tubulointerstitial nephropathy, osteoporosis, and lung and bladder cancer [12,13,14,15,16,17]. Lead, even in low concentrations, has been demonstrated to exhibit cardiotoxic properties, resulting in endothelial dysfunction, hypertension, and accelerated atherogenesis [12,13,14,15,16,18]. Nickel, despite its lower concentrations in tobacco, is recognised for its respiratory toxicity and sensitising potential, and selected nickel compounds are classified by the IARC as Group 1 carcinogens. Although copper is an essential element, it has the potential to become detrimental when inhaled over an extended period, contributing to oxidative stress and airway inflammation [12,14,15,16]. At the molecular level, heavy metals have been demonstrated to induce oxidative stress and chronic inflammation, resulting in increased cellular damage and synergistic potentiation of the toxicity of other components of tobacco smoke [12,13,14,15,16,17,18]. The participation of both Ni and Cu in redox cycling reactions has been shown to result in the intensification of oxidative injury [12,14,15,16].

From an epidemiological perspective, exposure to heavy metals present in tobacco products has been demonstrated to correlate with an increased incidence of respiratory cancers, cardiovascular diseases, and chronic kidney disease in populations with the highest smoking rates [2]. Despite the comparatively limited extant evidence, smoking has been shown to potentially elevate systemic levels of nickel and copper, accompanied by biomarkers of oxidative stress and subtle impairments in respiratory function [2,8]. Concurrently, the absence of globally harmonised tobacco quality control standards engenders considerable variation in the content of toxic elements in products available on the market. These disparities translate directly into differences in toxicological exposure and health burden, especially in populations with lower socioeconomic status [2,5].

In view of the mounting international political momentum to restrict tobacco smoking, novel challenges are emerging concerning the assurance of quality and the regulation of tobacco products. The heterogeneity of products available on the market—encompassing both conventional cigarettes and alternative products such as heated tobacco and e-cigarettes—engenders substantial challenges in ensuring uniform toxicological standards [19,20]. Despite the numerous initiatives undertaken by the WHO and other public health organisations, inequalities in the heavy metal content of tobacco products persist, reflecting the absence of consistent regulation at the international level [3,5]. In this context, it is imperative to develop and implement globally harmonised standards for permissible concentrations of toxic elements in raw tobacco and final products. The introduction of such restrictions would be a significant step towards reducing population exposure disparities and limiting the long-term health effects of tobacco consumption, in accordance with the principles of primary prevention and the holistic One Health approach [21]. The latter integrates human, environmental, and industrial health into a coherent model of public health protection.

The present study aimed to determine the content of selected heavy metals—cadmium, lead, nickel, and copper—in cigarettes available on the European market. Based on these measurements, non-carcinogenic and carcinogenic health risks associated with tobacco product consumption were estimated under various exposure scenarios. While it is well established that heavy metals and tobacco smoking in general have detrimental health effects, this study specifically aimed to compare the levels of exposure across different scenarios and to examine how these exposures vary between manufacturers producing specific cigarette brands. Risk assessments were conducted both on a cumulative basis for each brand and individually for each analyzed sample.

## 2. Materials and Methods

### 2.1. Sample Collection

The study material comprised 119 tobacco samples from commercially available cigarettes representing four major tobacco producers: Producer 1 (48 samples), Producer 2 (13 samples), Producer 3 (22 samples), and Producer 4 (36 samples)—(Table S1—Supplementary Material). Each analytical sample consisted of two to three cigarettes (in the case of slim cigarettes, 3–4 pieces; in the case of regular cigarettes, 2 pieces), representing a representative sample of material for a given brand. Before analysis, each sample was homogenized for approximately 30 s using a vibratory mill LMW-s (TESTCHEM, Pszów, Poland). The homogenized tobacco was then used to prepare analytical portions of 0.5 g, accurately weighed to 0.0001 g using an analytical balance (PS 750/X, RADWAG, Radom, Poland).

### 2.2. Laboratory Analyses

The digestion of the samples was performed in a multi-station microwave digestion system, ETHOS UP (MILESTONE, Sorisole, Italy). Each 0.5 g sample was placed in a Teflon vessel, to which 9 mL of 65% nitric acid (HNO_3_) (Merck, Darmstadt, Germany) and 1 mL of 30% hydrogen peroxide (H_2_O_2_) (Stanlab, Lublin, Poland) were added. The digestion process was carried out in three stages under the following conditions: (1) 20 min at 210 °C and 1800 W, (2) 15 min at 210 °C and 1800 W, followed by (3) 20 min cooling of the samples to room temperature. The digested solutions were quantitatively transferred to 50 mL volumetric flasks and diluted to the mark with deionized water.

### 2.3. Determination of Heavy Metals

The concentrations of cadmium (Cd) and lead (Pb) were determined using electrothermal atomic absorption spectrometry (ET-AAS) with a Savanta Sigma atomic absorption spectrometer (GBC Scientific Equipment, Melbourne, Australia). The concentrations of nickel (Ni) and copper (Cu) were determined using inductively coupled plasma optical emission spectrometry (ICP-OES) with an Ultima Expert LT spectrometer (HORIBA Scientific, Kyoto, Japan). Analytical parameters, including wavelength, lamp current, slit width, limit of detection (LOD), and limit of quantification (LOQ), are presented in Table 1. The limits of detection (LOD) and quantification (LOQ) for each element were determined based on the analysis of twenty mineralized blank samples. For each analytical series, the instrumental signals obtained for the blank samples were measured, and the standard deviation (SD) of these signals was calculated. The LOD was defined as the sum of the mean signal values of the blank samples and three times the standard deviation, whereas the LOQ was defined as the sum of the mean signal values of the blank samples and six times the standard deviation. The use of two analytical techniques was dictated by differences in detection limits of the instruments—ET-AAS offers higher sensitivity for Cd and Pb, whereas ICP-OES allows for accurate quantification of Ni and Cu at higher concentration ranges. Table 2 provides the calibration performance data, namely the calibration ranges, coefficients of determination (r^2^), and recovery values, which collectively confirm the accuracy, precision, and reliability of the analytical procedures employed in this study.

Automatic dilution was employed for samples exhibiting concentrations that lay outside the established calibration range. This ensured that the final measurements fell within the specified range, thus ensuring the accuracy and reliability of the results.

The standard solutions used for instrument calibration comprised certified reference materials obtained from multiple reputable suppliers to ensure analytical accuracy and traceability. For copper (Cu), calibration employed solutions from CPAchem (No. 923787), AccuStandard (221075081), and Spex CertiPrep (25-76 CUT), while nickel (Ni) calibration utilized standards from CPAchem (No. 923786), Agilent Technologies (0104978089), and Spex CertiPrep (24-187 NIT). Lead (Pb) determinations were calibrated using two independent Spex CertiPrep solutions (25-53 PBT and 26-71 PBT), providing internal cross-validation of measurement consistency. For cadmium (Cd), calibration was based on reference solutions from AccuStandard (220065069) and Agilent Technologies (5190-8491). The use of multiple certified sources for each analyte minimized supplier-specific bias, enhanced metrological reliability, and ensured conformity with international analytical quality standards.

The sample enrichment procedure involved the addition of a specific volume of a certified standard solution of known concentration (Cd—10 µL; Pb—20 µL; Cu—2.5 mg/L; Ni—4 mg/L) to the matrix sample. The prepared samples were then subjected to a microwave mineralization process that was identical to the process used for the other samples that were tested.

### 2.4. Assessment of Non-Carcinogenic Health Risk

Non-carcinogenic health risk associated with exposure to heavy metals present in tobacco was evaluated separately for ingestion and inhalation exposure pathways. The calculations were performed using Equations (1)–(4).

#### 2.4.1. Average Daily Dose (ADD)

The average daily dose via ingestion (ADD_ing) was calculated according to Equation (1) [22]:

(1) $$ADD\_ing=\frac{C\times EF\times ED\times IngR}{AT\times BW}$$

where: C—arithmetic mean concentration of the metal in the sample (mg/kg), EF—exposure frequency (days/year), ED—exposure duration (years), IngR—ingestion rate (mg/day), AT—averaging time (days), BW—body weight (kg).

Similarly, the average daily dose for the inhalation pathway (ADD_inh) was determined using Equation (2) [23]:

(2) $$ADD\_inh=\frac{C\times EF\times ED\times InhR}{AT\times BW}$$

where InhR represents the inhalation rate, expressed as the inhaled air volume during cigarette smoking (m^3^/day). The numerical values adopted for the parameters (EF, IR, ED, AT, BW) are summarized in Table 3.

#### 2.4.2. Hazard Quotient (HQ)

The Hazard Quotient (HQ) is a dimensionless metric applied to evaluate the potential non-carcinogenic health risk associated with human exposure to chemical contaminants, including heavy metals. It represents the ratio between the estimated daily exposure dose (mg/kg·day) and the reference dose (RfD)—a toxicological benchmark defining the maximum level of exposure expected to be without appreciable adverse health effects during a lifetime. The hazard quotient via ingestion (HQ_ing) for each metal was calculated using Equation (3) [29]:

(3) $$HQ\_ing=\frac{ADD\_ing}{RfD}$$

where RfD is the reference dose (mg/kg/day) for each metal (values in Table 4).

The hazard quotient for the inhalation pathway (HQ_inh) was calculated according to Equation (4) [29]:

(4) $$HQ\_inh=\frac{ADD\_inh}{RfC}$$

where RfC is the reference concentration (mg/m^3^/day) for each metal (Table 4).

An HQ ≤ 1 indicates that the exposure is below the critical threshold and is unlikely to result in harmful health outcomes. However, an HQ > 1 denotes a potential for non-carcinogenic toxicity, implying that the intake of the contaminant may exceed the organism’s detoxification capacity [29,30,31].

**Table 4:** The values of RFD, RFC, CSF, and IUR used in the study.

Element	RfD (mg/kg)	Reference	RfC (mg/m^3^)	Reference	CSF (mg/kg-day)	Reference	IUR (mg/m^3^-day)	Reference
Cd	0.001	[32]	0.00001	[33]	0.380	[34]	0.0018	[32]
Cu	0.04	[35]	0.04	[36]	-	-	-	-
Ni	0.02	[37]	0.00009	[37]	1.7	[36]	0.00024	[37]
Pb	0.004	[38]	0.00015	[39]	0.0085	[38]	0.000012	[39]

### 2.5. Assessment of Carcinogenic Health Risk

Carcinogenic health risk was assessed for both ingestion and inhalation exposure pathways using Equations (5)–(8) [29].

#### 2.5.1. Lifetime Average Daily Dose (LADD)

For the ingestion route:

(5) $$LADD\_ing=\frac{C\times EF\times ED\times IngR}{AT\times BW}$$

and for the inhalation route:

(6) $$LADD\_inh=\frac{C\times EF\times ED\times InhR}{AT\times BW}$$

The parameters are defined as in Section 2.4.1, while the averaging time (AT) was assumed to be 70 years × 365 days = 25,550 days, corresponding to a human lifetime (Table 3).

#### 2.5.2. Lifetime Cancer Risk (LCR)

The Cancer Risk (CR), or Lifetime Cancer Risk (LCR), quantifies the probability of developing cancer over a lifetime due to exposure to a carcinogenic substance. It is calculated as the product of the estimated exposure dose and the slope factor (SF)/inhalation unit risk (IUR), which represents the carcinogenic potency of a compound. The lifetime cancer risk (LCR) for each metal was calculated according to Equations (7) and (8) [29]:

(7) LCR_ing=LADD_ing× CSF

(8) LCR_inh=LADD_inh×IUR

where: CSF—cancer slope factor (mg/kg/day), IUR—inhalation unit risk (mg/m^3^/day). The values of CSF and IUR for each metal are provided in Table 4.

According to international health risk assessment standards (e.g., U.S. EPA), LCR < 1 × 10^−6^ denotes a negligible cancer risk, CR between 1 × 10^−6^ and 1 × 10^−4^ represents an acceptable or tolerable range of risk for regulatory purposes, LCR > 1 × 10^−4^ indicates a potentially unacceptable cancer risk, suggesting that the exposure level may significantly increase lifetime cancer probability [40,41].

In this study, five exposure scenarios were established to assess the intake of heavy metals present in the analyzed cigarettes, considering both inhalation and ingestion pathways. These scenarios differed in the number of cigarettes smoked per day, corresponding to one, five, ten, fifteen, and twenty cigarettes, respectively. The carcinogenic risk assessment included exposure to three heavy metals: cadmium (Cd), lead (Pb), and nickel (Ni). For the non-carcinogenic risk assessment, copper (Cu) was additionally included alongside cadmium, lead, and nickel. This approach allowed for a comprehensive evaluation of how varying levels of cigarette consumption may influence the potential health risks associated with the presence of heavy metals in cigarettes.

### 2.6. Statistical Analysis

Statistical analyses were performed using Statistica 13.3 (StatSoft, Kraków, Poland) for post-hoc testing and correlation analysis. For each metal (Cu, Pb, Ni, Cd), the Shapiro–Wilk test was used to assess normality of the distribution within each company group. Homogeneity of variances across company groups was assessed using Levene’s test (median). Between-group differences were tested separately for each metal. Where the assumption of normality (for all groups) and homoscedasticity were both met, one-way analysis of variance (ANOVA) was planned; where these assumptions were not satisfied, the Kruskal–Wallis rank-sum test was applied. For pairwise post-hoc comparisons, we used Tukey’s HSD following ANOVA, and a pairwise Mann–Whitney U test with Bonferroni correction (used as the non-parametric alternative to Dunn’s test) following Kruskal–Wallis. Correlations between individual metal concentrations (all pairwise combinations) were assessed using Pearson’s correlation coefficient when both variables showed approximate normality; otherwise, Spearman’s rank correlation was used. A significance threshold of α = 0.05 was adopted for all tests; for post-hoc pairwise comparisons, *p*-values were adjusted by the Bonferroni method.

## 3. Results

### 3.1. Concentration of Cadmium, Copper, Nickel, and Lead in Analyzed Cigarette Samples

The concentrations of heavy metals determined in the analyzed cigarette samples (*n* = 119) originating from four major tobacco companies showed considerable variability among the investigated elements (Table 5). Among the four metals studied, copper (Cu) and lead (Pb) exhibited the highest mean concentrations, whereas cadmium (Cd) and nickel (Ni) were present at comparatively lower levels. None of the samples were below the limit of quantification (LOQ), indicating a consistent presence of Cd in all analyzed cigarettes. For copper (Cu), concentrations varied between 3.4 and 92.6 mg/kg. This suggests that Cu is a commonly occurring element in the analyzed cigarette samples, likely associated with agricultural practices and manufacturing processes. Nickel (Ni) showed the highest proportion of results below the LOQ (58.8%), suggesting generally low levels of this metal in the samples. The most pronounced variability was observed for lead (Pb). Despite a relatively low median value of 1.3 mg/kg, the mean concentration (46.5 mg/kg) was markedly elevated, indicating the presence of outlier samples with exceptionally high Pb content.

Overall, Cu and Pb were the dominant elements, while Cd and Ni occurred at lower levels. The wide range of concentrations, particularly for Pb, reflects significant differences between cigarette brands and manufacturers, likely resulting from variations in tobacco origin, cultivation conditions, and production technologies (Table S2).

The concentrations of Cd, Cu, Ni, and Pb in the analyzed cigarette samples were evaluated separately for four major tobacco companies (Table 6). The obtained data revealed noticeable inter-company differences in the levels of heavy metals, although similar overall patterns were observed across all groups. Cadmium (Cd) concentrations were relatively consistent among the companies, with median values ranging from 0.7 to 0.9 mg/kg and mean values between 0.8 and 1.2 mg/kg. None of the samples contained Cd below the limit of quantification (LOQ), indicating a uniform presence of this element in all products. The Cd range (0.3–5.0 mg/kg) was comparable across the four companies. For copper (Cu), concentrations showed moderate variability. Median values ranged from 9.3 to 12.7 mg/kg, and the mean concentrations varied from 9.5 to 13.7 mg/kg. The lowest proportion of samples below the LOQ was observed for company 1 (4.2%), whereas the highest was for company 2 (23.1%). The overall Cu content ranged from 3.4 to 92.6 mg/kg, suggesting potential differences in the tobacco blend composition and cultivation environment among the manufacturers. Nickel (Ni) exhibited the highest frequency of results below the LOQ, with values ranging from 41.7% to 72.7%, confirming its generally low concentrations in the analyzed cigarettes. Median Ni levels ranged between 2.4 and 2.6 mg/kg, and mean concentrations from 3.5 to 6.7 mg/kg. The Ni range (1.7–15.4 mg/kg) was relatively narrow and consistent among the companies. The most pronounced differences were observed for lead (Pb). Although the median concentrations were generally low (0.4–2.3 mg/kg), the mean values varied widely, from 17.6 mg/kg (company 2) to 56.5 mg/kg (company 1). This discrepancy suggests the occurrence of individual samples with exceptionally high Pb contents, particularly in products from company 1. Indeed, Pb concentrations varied from 0.2 to 1633.3 mg/kg, demonstrating significant heterogeneity within and between companies.

In summary, Cu and Pb were the predominant elements across all tobacco companies, while Cd and Ni were detected at comparatively lower concentrations. Despite similar median values among companies, the large variation in Pb and Cu levels indicates differences in raw material origin, soil contamination, and production processes. These findings highlight the need for stricter monitoring of heavy metal content in tobacco products to ensure consumer safety and regulatory compliance.

In this study, the term “high-risk outliers” refers to individual cigarette samples that displayed exceptionally elevated concentrations of one or more toxic metals, markedly exceeding the distribution observed for the remaining dataset. These samples represent statistically and toxicologically significant deviations, characterised by disproportionately high levels—most notably of lead—which substantially influence both the mean concentration values and the subsequent health-risk estimations. Given their potential to distort aggregated risk metrics and to indicate localized contamination events or irregularities in raw-material sourcing, these high-risk outliers are discussed separately in the context of the health-risk assessment presented in the following sections.

In the context of this study, the term “high inter-brand variability” refers to the heterogeneity observed between individual cigarette samples or brands, irrespective of the manufacturer. This variability is driven largely by a small number of samples exhibiting disproportionately elevated concentrations of certain metals—particularly lead—which substantially deviate from the central distribution. These sample-level deviations, referred to as “high-risk outliers,” substantially influence descriptive statistics such as the overall range and mean values. Importantly, this concept differs from “between-company differences,” which are based on statistical comparisons of grouped data representing each tobacco manufacturer. While inter-brand variability was indeed high, especially for Pb, these extreme values were not clustered within a single producer, and therefore did not translate into statistically significant between-company differences.

### 3.2. Assessment of Non-Carcinogenic Health Risk Associated with Exposure to Heavy Metals in the Analyzed Cigarettes

The estimated Hazard Quotient (HQ) values were calculated based on the Average Daily Dose (ADD) for inhalation (Table S3) and ingestion (Table S5) exposure to cadmium, copper, nickel, and lead derived from the analyzed cigarette samples. The assessment considered five exposure scenarios corresponding to different smoking habits: 1, 5, 10, 15, and 20 cigarettes per day. The HQ values were computed separately for products from four major tobacco companies.

Under the lowest exposure scenario (1 cigarette per day), the calculated hazard quotient (HQ) values for copper and nickel remained below 1, suggesting that occasional smoking at this frequency is unlikely to pose a significant non-carcinogenic health risk. It is important to emphasize that these HQ values were derived from aggregated data across all cigarette samples from a given tobacco company, rather than representing individual cigarette-level HQ measurements (Table 7). In the case of copper, the HQ value did not exceed 1 in the other four scenarios either. However, several HQ values exceeded the critical threshold of 1.0, as indicated by the bolded results in Table 6. These exceedances suggest a potential health risk associated with chronic exposure to specific metals, particularly lead, cadmium, and nickel. Cadmium in cigarettes from company 2, where HQ values surpassed 1.0 beginning with the 1-cigarette-per-day scenario (Table 7). Analysis of HQ values calculated for individual cigarette samples revealed that, even under the first inhalation exposure scenario (5 cigarettes per day), certain metals posed a potential non-carcinogenic health risk. Specifically, for 12 samples, the HQ value for cadmium exceeded the threshold of 1, indicating possible concern. Similarly, 11 samples exhibited HQ values greater than 1 for lead, while two samples exceeded this threshold for nickel. Notably, one sample—C314/II/17/18 (from company 4)—demonstrated HQ values exceeding 1 for both cadmium and lead, highlighting the variability in toxic metal exposure across individual cigarette products within the same exposure scenario. The HQ values calculated for inhalation exposure to heavy metals for each of the cigarette samples tested are presented in Table S4.

The estimated non-carcinogenic health risk via the ingestion exposure pathway for the assumed scenarios involving cadmium, lead, nickel, and copper did not exceed the reference value of 1 in any scenario, for any cigarette company (Table 8). Therefore, it can be concluded that, under the assumed conditions of oral exposure to heavy metals contained in cigarettes, and given the observed levels of contamination and the assumed smoking frequency, no potential non-carcinogenic health effects are expected to occur in the exposed population. However, this conclusion should be formulated with caution, as it refers exclusively to the ingestion exposure pathway, while the situation for inhalation exposure appeared to be markedly different. Secondly, the data presented in Table 8 refer to the summed ingestion exposure calculated for all products of specific cigarette companies. However, when the results were analyzed individually for each sample, such exceedances were indeed observed. All these exceedances were associated with lead (Pb). Specifically, for sample C128/II/22/23 (from company 3), the HQ value exceeded the permissible limit already in the second scenario (corresponding to smoking 5 cigarettes per day), and this exceedance persisted in the third, fourth, and fifth scenarios. For sample C129/2/22/23 (from company 1), exceedances of the HQ value for lead were noted in the third, fourth, and fifth scenarios. In turn, for sample C130/2/22/23 (from company 3), the HQ value for lead exceeded the permissible limit in the fourth and fifth scenarios. Such individual assessment and estimation of health risk, in this case non-carcinogenic risk, for a specific type of cigarette is particularly important, considering that smokers rarely change the brand or type of cigarettes they use daily. In most cases, individuals remain loyal to a single brand and product for many years. Therefore, it should be emphasized that an individualized approach to risk estimation for each specific sample is of particular importance. It is, of course, well known that the levels of contaminants in the analyzed samples may vary in subsequent production batches. These contaminant levels may, in some cases, remain below the threshold at which the HQ value would exceed 1, and thus would theoretically not be associated with potential non-carcinogenic health effects. However, such studies should be periodically repeated, as the degree of contamination may depend on various factors, such as the specific origin of the tobacco material or the sources of raw materials used by a given brand or manufacturer. The HQ values calculated for ingestion exposure to heavy metals for each of the cigarette samples tested are presented in Table S6.

It should be emphasised that the Hazard Quotient (HQ) is a screening-level ratio comparing estimated exposure to a reference dose or reference concentration, and should not be interpreted quantitatively beyond the threshold of 1.0. In other words, if HQ > 1, a potential non-carcinogenic hazard is indicated—but the magnitude of HQ does not correspond linearly to increasing probability or severity of effect [8,42].

### 3.3. Assessment of Carcinogenic Health Risk Associated with Exposure to Heavy Metals in the Analyzed Cigarettes

Carcinogenic health risk was estimated analogously to non-carcinogenic risk, based on Lifetime Average Daily Dose (LADD) values for both inhalation and ingestion pathways. Lifetime Cancer Risk (LCR) was then calculated for elements with complete input data, namely cadmium, lead, and nickel. The LADD and LCR values for each element in individual cigarette samples are reported in Tables S7–S10. For the Lifetime Cancer Risk (LCR) assessment, exposure was also considered across five different scenarios, ranging from 1 to 20 cigarettes per day.

The analysis of the estimated inhalation-related carcinogenic health risk associated with cadmium (Cd), nickel (Ni), and lead (Pb) revealed a significant lifetime cancer risk (LCR) for products from all tobacco companies included in the study. For each company, the risk was evaluated cumulatively across all cigarette samples representing individual brands. Notably, a considerable cancer risk was already evident under the first exposure scenario, corresponding to the inhalation of smoke from one cigarette per day. This trend was consistent across all analyzed elements (Cd, Ni, and Pb) and for all exposure scenarios and tobacco companies. Among the analyzed metals, the highest LCR values were recorded for lead (Pb) (Table 9). In Table 9, LCR values exceeding the acceptable limit (1 × 10^−4^) are highlighted in bold, which applies to all reported results. Conversely, in Supplementary Table S8, which presents individual LCR values calculated for each cigarette sample, bold formatting is not used, as all estimated LCR values surpass the permissible threshold regardless of the exposure scenario, element, or sample. These findings collectively indicate that inhalation exposure to Cd, Ni, and Pb from cigarette smoking poses a carcinogenic risk exceeding the accepted safety level for all analyzed tobacco products, even under minimal exposure conditions.

Considering the acceptable LCR threshold of 1 × 10^−4^, values exceeding this limit are highlighted in bold in Table 10. All such exceedances concern lead (Pb), which surpassed the permissible level in every ingestion exposure scenario, regardless of the manufacturer. This finding indicates that even minimal daily ingestion exposure resulting from cigarette consumption could pose a carcinogenic risk associated with Pb intake that exceeds the generally accepted safety level. The highest overall LCR values were observed for products from company no. 3, suggesting that cigarettes from this manufacturer contribute most significantly to the potential lifetime cancer risk associated with heavy metal ingestion. Data presented in Supplementary Table S10, which reports individual LCR values for each cigarette sample, further support these results. Exceedances of the acceptable LCR level (1 × 10^−4^) were found for Pb in 31 out of 119 analyzed samples. Importantly, only one sample (C314/II/17/18), originating from manufacturer no. 4, exhibited an LCR for Cd exceeding the permissible value, reaching 1.63 × 10^−4^ under the fifth ingestion exposure scenario (equivalent to the consumption of 20 cigarettes per day). Overall, the results demonstrate that lead is the dominant contributor to carcinogenic risk from ingestion exposure among the analyzed metals, while cadmium and nickel generally remain below the acceptable level. However, the isolated exceedance observed for Cd highlights potential variability in metal concentrations across cigarette brands, which may substantially influence individual risk levels. These findings emphasize the necessity for ongoing monitoring of toxic metal content in tobacco products and underscore the potential health hazards arising from ingestion exposure, even at relatively low levels of cigarette consumption.

### 3.4. Results of Statistical Analyses

Shapiro–Wilk tests performed within each company indicated that not all groups met the normality assumption for the metals tested. Levene’s test (median) indicated no strong evidence of heterogeneity of variances across companies for any metal (Levene *p* > 0.05 for Cu, Pb, Ni, and Cd). Because the normality assumption was not satisfied across all groups, Kruskal–Wallis tests were used to evaluate between-company differences for each metal. The Kruskal–Wallis *p*-values were as follows: Cu: *p* = 0.565, Pb: *p* = 0.280, Ni: *p* = 0.892, Cd: *p* = 0.287. None of the overall tests reached the α = 0.05 significance threshold. Accordingly, post-hoc pairwise comparisons were performed using pairwise Mann–Whitney U tests with Bonferroni correction (applied to control the familywise error rate). After Bonferroni adjustment, no pairwise comparison between companies was statistically significant (all adjusted *p* ≥ 0.05) for any of the four metals.

**Figure 1:**
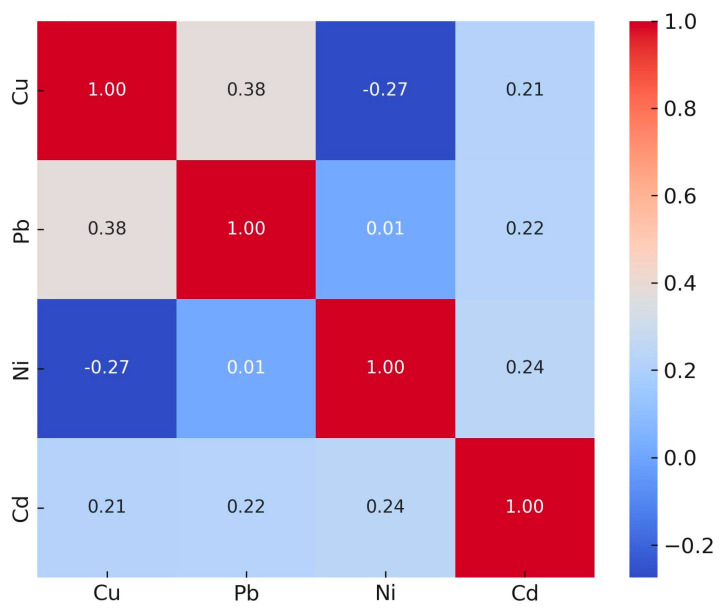
Spearman correlation matrix of heavy metal concentrations in cigarette samples.

Pairwise correlations were computed for each metal pair, and the correlation method was chosen depending on variable normality (Pearson when both variables were approximately normal, otherwise Spearman). For the dataset as a whole, Spearman correlations were used for all pairs (Pearson was not indicated because one or both variables failed normality). The correlation coefficients (r) and *p*-values were (Figure 1): Cu–Pb: r = 0.383, *p* = 0.065; Cu–Ni: r = −0.274, *p* = 0.195; Cu–Cd: r = 0.215, *p* = 0.314; Pb–Ni: r = 0.014, *p* = 0.949; Pb–Cd: r = 0.221, *p* = 0.300; Ni–Cd: r = 0.240, *p* = 0.259. None of these pairwise correlations reached statistical significance at the 0.05 level.

In summary, across the examined cigarette samples, concentrations of Cu, Pb, Ni, and Cd did not differ significantly between the four examined companies (Kruskal–Wallis *p* > 0.05 for all metals); pairwise post-hoc tests (Mann–Whitney U with Bonferroni correction) revealed no significant between-company differences. Although substantial variability was observed within the dataset—including extreme values for Pb and, to a lesser extent, Cu—this variability occurred primarily at the level of individual brands or samples rather than systematically between tobacco companies. Consequently, the presence of high inter-brand variability did not manifest as statistically significant between-company differences. This finding indicates that extreme concentration values were distributed across manufacturers rather than being attributable to a single producer, thus reinforcing the distinction between sample-level heterogeneity and group-level statistical differences. Similarly, although some metal pairs showed moderate correlations (for example, Cu–Pb, r ≈ 0.38), no pairwise metal correlation reached statistical significance (*p* > 0.05). All tests reported above used a two-tailed α = 0.05; post-hoc *p*-values were adjusted using the Bonferroni method.

## 4. Discussion

The presence of heavy metals in tobacco has been identified as a significant toxicological factor associated with cigarette smoking. A review of 76 previously published studies in this area, conducted by Felix & Ntarisa (2024) [43], revealed significant variation in the levels of Cd, Pb, and Ni in cigarettes from different manufacturers worldwide. This suggests that cultivation conditions and the origin of the raw materials have a significant impact on the quality of the product.

It is important to note that the high variability observed in the concentrations of metals—particularly the wide range reported for Pb—reflects heterogeneity at the level of individual cigarette samples rather than consistent manufacturer-specific patterns. This distinction explains why descriptive statistics indicated substantial inter-brand variability, while formal statistical testing did not identify significant between-company differences. Such patterns suggest that irregularities likely stem from batch-level or raw-material differences rather than systematic differences in production practices across manufacturers.

The present study assessed the content of selected toxic elements (Cd, Pb, Ni, and Cu) in cigarettes available on the Polish and European markets, including four major manufacturers. The obtained results confirmed both the widespread presence of these elements in tobacco and the significant variation between brands, particularly with respect to Pb. In addition, the estimated HQ and LCR values indicated that even minimal inhalation exposure (1 cigarette per day) exceeds the international acceptable risk thresholds for Cd, Ni, and Pb. These results are consistent with those of other global studies, which indicate that cigarettes are one of the most significant and well-documented sources of heavy metal exposure in smokers [8,11,14,43].

The concentrations of Cd, Ni, and Cu in this study, ranging from 0.3–8.6 mg Cd/kg, 1.1–15.4 mg Ni/kg, and 3.4–92.6 mg Cu/kg, respectively, are slightly higher in comparison with the concentrations reported in the international literature. The findings of the present study demonstrated also that the concentrations of lead (Pb) varied significantly, with a range of 0.2–1633.0 mg/kg. However, it was observed that only a limited number of samples exhibited concentrations that deviated from the median value of 1.3 mg Pb/kg. Isolated reports support the possibility of locally high Pb contamination in tobacco, particularly in regions with intensive use of phosphate fertilisers and active industrial emissions [24,43,44]. This finding indicates that the remarkably elevated Pb concentrations detected in our study may be attributable to contaminated primary crops or heterogeneity in the raw tobacco utilized by individual producers.

In a study conducted by Hasan et al. (2023) [44], the concentrations of ten heavy metals (Pb, Cd, Cr, As, Co, Ni, Mn, Fe, Cu, Zn) in commonly consumed cigarette brands in Bangladesh were assessed. The following concentration ranges were observed for Cd: 0.55–1.03 mg/kg, for Ni: 2.59–3.03 mg/kg, for Cu: 146.6–217.7 mg/kg and for Pb: 0.46–1.05 mg/kg. In addition, Ntarisa’s study (2024) [24] on the concentrations of heavy metals in commonly consumed cigarette products in Tanzania yielded comparable concentrations of Cd, Ni, and Cu, respectively. The concentrations of cadmium (Cd), nickel (Ni), and copper (Cu) in the sample range from 0.4 to 0.66 milligrams per kilogram (mg/kg), from 0.11 to 2.69 mg/kg, and from 6.94 to 16.31 mg/kg, respectively. Caruso et al. (2014) [45] and Pappas et al. (2014) [46] analysed the Cd content in cigarettes available in the USA and obtained similar results, noting that Cd is one of the most stable and reproducible metals in tobacco products, a consequence of the ability of tobacco plants to hyperaccumulate this element.

In the domain of toxicological quantitative health risk assessment, HQ and LCR indices are utilised, among other methodologies, contingent upon whether the assessment pertains to non-cancer or cancer risk [29]. The results of the health risk assessment resulting from the use of tobacco products containing heavy metals conducted in this study showed that inhalation exposure to Cd, Ni, and Pb, even in a scenario assuming smoking one cigarette per day, consistently exceeded the internationally accepted risk thresholds for both non-carcinogenic and carcinogenic risks. Concomitant findings have been reported by other researchers, suggesting a substantial toxicological and carcinogenic hazard when smokers consume between several and a dozen cigarettes per day [44,46,47]. Furthermore, Pappas et al. (2014) [46] demonstrated that the transfer of Cd and Pb from tobacco to smoke is high and can reach as much as 50–70%, leading to significant inhalation exposure. In the study by Vlachou et al. (2021) [47], it was found that the excess lifetime cancer risk (ELCR) values for Cd in the Austrian smoking population exceeded EPA standards by several fold. Hasan et al. (2023) [44] also reported HQ > 1 (i.e., indicated potential concern for non-cancer health effects) for Cu, Zn, Mn, and Ni for popular cigarette brands in Bangladesh. A health risk assessment of Tanzanian smokers also indicated that the inhalation route raises concerns: The concentrations of metals in the respiratory route have been found to exceed the acceptable limits for some of these metals. However, the carcinogenic risk was assessed as exceeding acceptable risk levels [24]. It is evident that substantial HQ and LCR exceedances signify that cigarettes persist in their capacity as a substantial yet widely underestimated source of heavy metal exposure. The presence of heavy metals in tobacco products is a pervasive global concern, as evidenced by a plethora of studies that have demonstrated significant inhalation exposure and the associated potential health risks to the population. In contrast to organic compounds, metals do not undergo biotransformation and accumulate in the body. This means that each additional year of smoking contributes to an increasing burden on the body.

Despite numerous studies demonstrating both non-carcinogenic and carcinogenic risks associated with exposure to heavy metals such as cadmium, lead, and nickel present in tobacco and cigarette smoke [43,45,46], a major challenge remains the practical limitation of reducing exposure in society. Tobacco use continues to be widespread—according to the latest WHO report, approximately 1 in 5 adults still use tobacco products [5]. Nicotine addiction, strong cultural habits, and social acceptance of smoking make it difficult to translate even clear scientific findings (e.g., exceedances of HQ or LCR values for heavy metals in cigarette smoke) into real-world interventions, such as source elimination, usage reduction, or systematic regulatory actions. Consequently, although evidence highlights the need for continuous monitoring, exposure reduction strategies, and adherence to international guideline values, implementation of effective measures is hindered by social and behavioral barriers. This complexity makes formulating actionable recommendations that can meaningfully improve public health considerably more challenging than the mere analysis of laboratory or environmental data.

### 4.1. Practical Implications

The findings of this study have several important practical implications for public health policy, regulatory agencies, and the tobacco industry. First, the substantial variability in heavy metal concentrations—particularly the extreme outliers observed for lead—indicates that current quality control measures applied during tobacco cultivation and cigarette manufacturing are insufficient to ensure consistent toxicological safety. The coexistence of substantial inter-brand variability and the absence of systematic between-company differences underscores the need for quality-control measures that operate not only at the manufacturer level but also at the level of individual production batches. This underscores the need for more stringent regulatory standards governing permissible concentrations of heavy metals in both raw tobacco and finished cigarette products. Implementing harmonized, internationally accepted limit values would help reduce disparities in exposure between brands and markets and would align with global tobacco control strategies recommended by the World Health Organization.

Second, the observation that carcinogenic risk thresholds were exceeded even under minimal smoking scenarios (one cigarette per day) highlights that heavy metal exposure from tobacco smoke represents a non-negligible contribution to the total toxicological burden among smokers. These results can support clinicians and public health practitioners in risk communication by providing quantifiable evidence that smoking-related metal exposure poses significant health risks independent of nicotine or organic toxicants. Furthermore, given that smokers typically remain loyal to a single brand over many years, the identification of high-risk outlier products emphasizes the importance of routine market surveillance and transparent reporting systems that would allow consumers and regulators to identify and respond to elevated contamination levels.

Third, the demonstrated inter-brand heterogeneity suggests that targeted regulatory interventions—such as mandatory source tracing, certification of agricultural inputs (e.g., fertilizers, water), and monitoring of tobacco leaf imports—could meaningfully reduce heavy metal content in cigarette products. For manufacturers, these findings provide a basis for implementing stricter procurement standards and quality assurance practices within their supply chains.

Finally, this study highlights the urgent need to incorporate heavy metal exposure into broader tobacco harm reduction frameworks and national cessation programs. By integrating toxic metal risks into educational campaigns, policymakers may enhance public awareness and further motivate smoking reduction or cessation efforts.

### 4.2. Strengths and Limitations of the Study

This study exhibits several methodological and analytical strengths. It is based on a relatively large and diverse sample set (n = 119) derived from four major tobacco companies, ensuring robust representativeness and enabling inter-brand comparisons. The use of two complementary analytical techniques (ET-AAS and ICP-OES) provided high sensitivity and quantification accuracy across metals present at both trace and higher concentrations. The study also applied internationally recognized toxicological risk-assessment frameworks (U.S. EPA, ATSDR) and incorporated multiple exposure scenarios reflecting a wide range of real-world smoking behaviors. Importantly, it included both carcinogenic and non-carcinogenic risk estimates and assessed these not only at the aggregated company level but also for each sample—an approach that enhances the relevance of results given smokers’ long-term brand loyalty. Rigorous statistical testing further strengthened the reliability of inter-company comparisons and minimized the likelihood of erroneous inferences.

Nonetheless, several limitations should be acknowledged when interpreting the findings. Metal concentrations were measured in raw tobacco rather than in mainstream smoke, meaning that the actual inhaled dose—affected by metal-specific transfer rates, cigarette design, and smoking topography—was not directly quantified. The exposure assessment was deterministic rather than probabilistic, which limits the representation of inter-individual variability and uncertainty. Only four metals (Cd, Pb, Ni, Cu) were analyzed, despite the known presence of additional toxic elements such as As, Cr, and Co in tobacco. Furthermore, no upstream agricultural or manufacturing data (e.g., soil contamination, fertilizer types, leaf origin) were available to contextualize the sources of metal contamination. Ingestion exposure estimates remain largely theoretical, as real ingestion from cigarette handling is minimal and difficult to quantify. These constraints highlight the need for future studies integrating smoke-yield measurements, probabilistic modelling, expanded metal panels, and supply chain characterization to more fully elucidate heavy-metal exposure associated with cigarette consumption.

## 5. Conclusions

This study demonstrates that cigarettes available on the Polish and broader European market contain measurable and, in several cases, elevated concentrations of toxic trace elements, particularly copper and lead, followed by cadmium and nickel. Although median concentrations for most metals remained relatively low, the wide concentration ranges—especially for Pb—indicate substantial heterogeneity between brands and manufacturers, likely related to differences in the geographical origin of tobacco leaves, agricultural practices, and production processes. The observed discrepancy between high inter-brand variability and the lack of significant between-company differences indicates that extreme contamination events are likely sporadic and not producer-specific, emphasizing the need for routine batch-level surveillance.

The health-risk assessment revealed that inhalation exposure to Cd, Ni, and Pb, even under the lowest smoking scenario of one cigarette per day, consistently exceeded internationally accepted carcinogenic risk thresholds. Several metals also produced non-carcinogenic hazard quotients (HQ > 1), particularly for inhalation exposure, suggesting the potential for chronic toxic effects involving renal, neurological, respiratory, and cardiovascular systems. Although ingestion exposure contributed less substantially to overall risk, exceedances of carcinogenic thresholds for Pb were observed across all scenarios, and isolated exceedances for Cd were detected in individual samples.

These findings indicate that tobacco smoke remains a significant and avoidable source of heavy-metal exposure. The large inter-brand variability and presence of high-risk outliers underscore the need for strengthened regulatory oversight, harmonized analytical standards, and systematic monitoring of toxic metal content in tobacco products. Given that most smokers consistently use the same brand for years, exposure from a single contaminated product may substantially elevate cumulative lifetime risk. The results support ongoing global public health initiatives aimed at reducing tobacco-related toxic metal exposure within the framework of the One Health approach.

## Figures and Tables

**Table 1:** The wavelength, lamp current, slit width, LOQ, and LOD of the analytical technique.

Element	Wavelength [nm]	Lamp Current [mA]	Slit Width [nm]	LOQ [mg/kg]	LOD [mg/kg]
Cd	228.8	3.0	0.5	0.02	0.01
Pb	217.0	5.0	1.0	0.16	0.09
Ni	341.5	-	-	1.65	1.00
Cu	324.7	-	-	0.09	0.05

**Table 2:** The calibration ranges, r^2^ values, and recoveries of the analytical techniques.

Trace Element	Analytical Technique	Calibration Range	*r^2^* Value	Recovery [%]	RSD [%]
Ni	ICP-OES	2.5–10.0 mg/L	0.999	85–98	3.7
Cu		2.5–10.0 mg/L	0.999	82–87	3.3
Cd	ET-AAS	5.0–20.0 µg/L	0.998–1	85–95	<20
Pb		10.0–40.0 µg/L	0.996–0.997	88–115	<20

**Table 3:** The values of EF, IR, ED, AT, and BW used in the study.

Parameter	Value	Reference
EF_ing_	365 days/year	[24,25,26,27]
EF_inh_	cigarettes per day (EF_day_) or year (EF_year_) (depends on the scenario)	[24,25,26,27]
IngR	1 cigarette = 59 mg/day (depends on the scenario)	[28]
InhR	1 cigarette = 0.0006 m^3^ (depends on the scenario)	[24,25,26,27]
ED	30 years	[24,25,26]
AT_for non-carcinogenic_	30 years × 365 days = 10,950 days	[24,25,26]
AT_for carcinogenic_	70 years × 365 days = 25,550 days	[24,25,26]
BW	70 kg	[24,25,26]

**Table 5:** Concentration [mg/kg] of cadmium, copper, nickel, and lead in the analyzed cigarette samples.

Element	<LOQ [%]	Q_1_	Median	Q_3_	Mean	Min–Max
Cd	0.0	0.6	0.8	1.0	1.0	0.3–8.6
Cu	7.6	9.6	11.0	12.7	12.3	3.4–92.6
Ni	58.8	1.7	2.4	4.1	3.4	1.1–15.4
Pb	32.7	0.3	1.3	9.7	46.5	0.2–1633.3

Notes. Q_1_—First quartile (25th percentile); Q_3_—Third quartile (75th percentile).

**Table 6:** Concentration [mg/kg] of cadmium, copper, nickel, and lead in the analyzed cigarette samples, taking into account the tobacco company.

Tobacco Company (Code)	<LOQ [%]				Q_1_				Median				Q_3_				Mean				Min–Max			
	Cd	Cu	Ni	Pb	Cd	Cu	Ni	Pb	Cd	Cu	Ni	Pb	Cd	Cu	Ni	Pb	Cd	Cu	Ni	Pb	Cd	Cu	Ni	Pb
1	0.0	4.2	41.7	29.2	0.5	10.1	2.2	0.3	0.8	11.0	2.6	0.4	1.0	12.0	5.4	7.8	0.8	13.7	4.1	56.5	0.3–3.7	7.6–92.6	1.7–11.7	0.2–1633.3
2	0.0	23.1	61.5	23.1	0.7	8.8	3.0	0.3	0.9	9.3	3.2	4.7	1.1	9.8	4.1	10.5	1.7	9.5	3.5	17.6	0.4–8.6	3.4–17.8	1.7–5.5	0.2–131.4
3	0.0	9.1	72.7	36.4	0.6	8.8	2.3	0.8	0.7	10.1	2.4	1.5	0.8	11.2	2.8	16.5	0.8	9.9	3.0	35.7	0.5–2.9	6.7–13.4	2.0–5.9	0.2–416.6
4	0.0	8.3	72.2	38.9	0.6	10.0	2.3	0.7	0.9	12.7	2.5	2.3	1.1	14.2	6.7	8.8	1.2	12.5	5.0	50.9	0.3–5.0	5.9–22.4	1.9–15.4	0.2–845.8

Notes. Q_1_—First quartile (25th percentile); Q_3_—Third quartile (75th percentile).

**Table 7:** Hazard Quotient (HQ) for inhalation exposure to cadmium, copper, nickel, and lead contained in the cigarettes tested, taking into account the tobacco company.

Tobacco Company (Code)	1 Cigarette per Day				5 Cigarettes per Day				10 Cigarettes per Day				15 Cigarettes per Day				20 Cigarettes per Day			
	Cd	Cu	Ni	Pb	Cd	Cu	Ni	Pb	Cd	Cu	Ni	Pb	Cd	Cu	Ni	Pb	Cd	Cu	Ni	Pb
1	6.94 × 10^−1^	2.94 × 10^−3^	3.89 × 10^−1^	**3.23**	**3.47**	1.47 × 10^−2^	**1.95**	**1.61 × 10^1^**	**6.94**	2.94 × 10^−2^	**3.89**	**3.23 × 10^1^**	**1.04 × 10^1^**	4.40 × 10^−2^	**5.84**	**4.84 × 10^1^**	**1.39 × 10^1^**	5.87 × 10^−2^	**7.79**	**6.45 × 10^1^**
2	**1.44**	2.04 × 10^−3^	3.32 × 10^−1^	**1.01**	**7.18**	1.02 × 10^−2^	**1.66**	**5.03**	**1.44 × 10^1^**	2.04 × 10^−2^	**3.32**	**1.01 × 10^1^**	**2.15 × 10^1^**	3.06 × 10^−2^	**4.98 × 10^1^**	**1.51 × 10^1^**	**2.87 × 10^1^**	4.08 × 10^−2^	**6.63**	**2.01 × 10^1^**
3	6.96 × 10^−1^	2.12 × 10^−3^	2.84 × 10^−1^	**2.04**	**3.48**	1.06 × 10^−2^	**1.42**	**1.02 × 10^1^**	**6.96**	2.12 × 10^−2^	**2.84**	**2.04 × 10^1^**	**1.04 × 10^1^**	3.18 × 10^−2^	**4.26**	**3.06 × 10^1^**	**1.39 × 10^1^**	4.24 × 10^−2^	**5.68**	**4.08 × 10^1^**
4	9.10 × 10^−1^	2.67 × 10^−3^	4.80 × 10^−1^	**2.91**	**4.55**	1.34 × 10^−2^	**2.40**	**1.46 × 10^1^**	**9.10**	2.67 × 10^−2^	**4.80**	**2.91 × 10^1^**	**1.37 × 10^1^**	4.01 × 10^−2^	**7.20**	**4.37 × 10^1^**	**1.82 × 10^1^**	5.35 × 10^−2^	**9.60**	**5.82 × 10^1^**

**Table 8:** Hazard Quotient (HQ) for ingestion exposure to cadmium, copper, nickel, and lead contained in the cigarettes tested, taking into account the tobacco company.

Tobacco Company (Code)	1 Cigarette per Day				5 Cigarettes per Day				10 Cigarettes per Day				15 Cigarettes per Day				20 Cigarettes per Day			
	Cd	Cu	Ni	Pb	Cd	Cu	Ni	Pb	Cd	Cu	Ni	Pb	Cd	Cu	Ni	Pb	Cd	Cu	Ni	Pb
1	7.39 × 10^−4^	2.43 × 10^−4^	1.52 × 10^−4^	8.60 × 10^−3^	3.70 × 10^−3^	1.22 × 10^−3^	7.62 × 10^−4^	4.30 × 10^−2^	7.39 × 10^−3^	2.43 × 10^−3^	7.62 × 10^−4^	8.60 × 10^−2^	1.11 × 10^−2^	3.65 × 10^−3^	3.81 × 10^−2^	1.29 × 10^−1^	1.48 × 10^−2^	4.87 × 10^−3^	3.05 × 10^−3^	1.72 × 10^−1^
2	1.30 × 10^−3^	6.04 × 10^−4^	2.05 × 10^−4^	1.04 × 10^−3^	6.51 × 10^−3^	3.02 × 10^−3^	1.02 × 10^−3^	5.21 × 10^−3^	1.30 × 10^−2^	6.04 × 10^−3^	1.02 × 10^−3^	1.04 × 10^−2^	1.95 × 10^−2^	9.06 × 10^−3^	5.12 × 10^−2^	1.56 × 10^−2^	2.60 × 10^−2^	1.21 × 10^−2^	4.10 × 10^−3^	2.08 × 10^−2^
3	6.75 × 10^−4^	2.22 × 10^−4^	1.62 × 10^−4^	2.65 × 10^−2^	3.38 × 10^−3^	1.11 × 10^−3^	8.11 × 10^−4^	1.33 × 10^−1^	6.75 × 10^−3^	2.22 × 10^−3^	8.11 × 10^−4^	2.65 × 10^−1^	1.01 × 10^−2^	3.33 × 10^−3^	4.06 × 10^−2^	3.98 × 10^−1^	1.35 × 10^−2^	4.43 × 10^−3^	3.25 × 10^−3^	5.30 × 10^−1^
4	9.34 × 10^−4^	2.45 × 10^−4^	1.81 × 10^−4^	3.43 × 10^−3^	4.67 × 10^−3^	1.22 × 10^−3^	9.07 × 10^−4^	1.71 × 10^−2^	9.34 × 10^−3^	2.45 × 10^−3^	9.07 × 10^−4^	3.43 × 10^−2^	1.40 × 10^−2^	3.67 × 10^−3^	4.53 × 10^−2^	5.14 × 10^−2^	1.87 × 10^−2^	4.90 × 10^−3^	3.63 × 10^−3^	6.86 × 10^−2^

**Table 9:** Lifetime Cancer Risk (LCR) for inhalation exposure to cadmium, nickel, and lead contained in the cigarettes tested, taking into account the tobacco company.

Tobacco Company (Code)	1 Cigarette per Day			5 Cigarettes per Day			10 Cigarettes per Day			15 Cigarettes per Day			20 Cigarettes per Day		
	Cd	Ni	Pb	Cd	Ni	Pb	Cd	Ni	Pb	Cd	Ni	Pb	Cd	Ni	Pb
1	**1.65 × 10^−2^**	**6.27 × 10^−1^**	**1.73 × 10^2^**	**8.27 × 10^−2^**	**3.13**	**8.66 × 10^2^**	**1.65 × 10^−1^**	**6.27**	**1.73 × 10^3^**	**2.48 × 10^−1^**	**9.40**	**2.60 × 10^3^**	**3.31 × 10^−1^**	**1.25 × 10^1^**	**3.46 × 10^3^**
2	**3.42 × 10^−2^**	**5.34 × 10^−1^**	**5.40 × 10^1^**	**1.71 × 10^−1^**	**2.67**	**2.70 × 10^2^**	**3.42 × 10^−1^**	**5.34**	**5.40 × 10^2^**	**5.14 × 10^−1^**	**8.01**	**8.10 × 10^2^**	**6.85 × 10^−1^**	**1.07 × 10^1^**	**1.08 × 10^3^**
3	**1.66 × 10^−2^**	**4.57 × 10^−1^**	**1.10 × 10^2^**	**8.30 × 10^−2^**	**2.28**	**5.48 × 10^2^**	**1.66 × 10^−1^**	**4.57**	**1.10 × 10^3^**	**2.49 × 10^−1^**	**6.85**	**1.64 × 10^3^**	**3.32 × 10^−1^**	**9.14**	**2.19 × 10^3^**
4	**2.17 × 10^−2^**	**7.73 × 10^−1^**	**1.56 × 10^2^**	**1.09 × 10^−1^**	**3.86**	**7.81 × 10^2^**	**2.17 × 10^−1^**	**7.73**	**1.56 × 10^3^**	**3.26 × 10^−1^**	**1.16**	**2.34 × 10^3^**	**4.34 × 10^−1^**	**1.55 × 10^1^**	**3.12 × 10^3^**

**Table 10:** Lifetime Cancer Risk (LCR) for ingestion exposure to cadmium, nickel, and lead contained in the cigarettes tested, taking into account the tobacco company.

Tobacco Company (Code)	1 Cigarette per Day			5 Cigarettes per Day			10 Cigarettes per Day			15 Cigarettes per Day			20 Cigarettes per Day		
	Cd	Ni	Pb	Cd	Ni	Pb	Cd	Ni	Pb	Cd	Ni	Pb	Cd	Ni	Pb
1	8.34 × 10^−7^	6.01 × 10^−7^	**1.74 × 10^−3^**	4.17 × 10^−6^	3.00 × 10^−6^	**8.68 × 10^−3^**	8.34 × 10^−6^	3.00 × 10^−6^	**1.74 × 10^−2^**	1.25 × 10^−5^	1.77 × 10^−6^	**2.60 × 10^−2^**	1.67 × 10^−5^	1.20 × 10^−5^	**3.47 × 10^−2^**
2	1.47 × 10^−6^	9.45 × 10^−7^	**2.10 × 10^−4^**	7.34 × 10^−6^	4.73 × 10^−6^	**1.05 × 10^−3^**	1.47 × 10^−5^	4.73 × 10^−6^	**2.10 × 10^−3^**	2.20 × 10^−5^	2.78 × 10^−6^	**3.15 × 10^−3^**	2.94 × 10^−5^	1.89 × 10^−5^	**4.20 × 10^−3^**
3	7.62 × 10^−7^	6.53 × 10^−7^	**5.35 × 10^−3^**	3.81 × 10^−6^	3.27 × 10^−6^	**2.67 × 10^−2^**	7.62 × 10^−6^	3.27 × 10^−6^	**5.35 × 10^−2^**	1.14 × 10^−5^	1.92 × 10^−6^	**8.02 × 10^−2^**	1.52 × 10^−5^	1.31 × 10^−5^	**1.07 × 10^−1^**
4	1.05 × 10^−6^	8.48 × 10^−7^	**6.92 × 10^−4^**	5.27 × 10^−6^	4.24 × 10^−6^	**3.46 × 10^−3^**	1.05 × 10^−5^	4.24 × 10^−6^	**6.92 × 10^−3^**	1.58 × 10^−5^	2.49 × 10^−6^	**1.04 × 10^−2^**	2.11 × 10^−5^	1.70 × 10^−5^	**1.38 × 10^−2^**

## Data Availability

The data that support the findings of this study are available from the corresponding author, upon reasonable request.

## Supplementary Materials

The following supporting information can be downloaded at: https://www.mdpi.com/article/10.3390/toxics13121088/s1, Table S1. Tobacco companies whose products were obtained for testing; Table S2. Concentration [mg/kg] of copper, lead, nickel, and cadmium in cigarettes available on the European market; Table S3. Average Daily Dose (ADD) [mg/kg] for inhalation exposure to copper, lead, nickel, and cadmium contained in the cigarettes tested; Table S4. Hazard Quotient (HQ) for inhalation exposure to copper, lead, nickel, and cadmium contained in the cigarettes tested; Table S5. Average Daily Dose (ADD) [mg/kg] for ingestion exposure to copper, lead, nickel, and cadmium contained in the cigarettes tested; Table S6. Hazard Quotient (HQ) for ingestion exposure to copper, lead, nickel, and cadmium contained in the cigarettes tested; Table S7. Lifetime Average Daily Dose (LADD) [mg/kg] for inhalation exposure to lead, nickel, and cadmium contained in the cigarettes tested; Table S8. Lifetime Cancer Risk (LCR) for inhalation exposure to lead, nickel, and cadmium contained in the cigarettes tested; Table S9. Lifetime Average Daily Dose (LADD) [mg/kg] for ingestion exposure to lead, nickel, and cadmium contained in the cigarettes tested; Table S10. Lifetime Cancer Risk (LCR) for ingestion exposure to lead, nickel, and cadmium contained in the cigarettes tested.

## Abbreviations

The following abbreviations are used in this manuscript:

ADD	Average Daily Dose
AT	Averaging Time
BW	Body Weight
C	Concentration
CSF	Cancer Slope Factor
ED	Exposure Duration
EF	Exposure Frequency
HQ	Hazard Quotient
IR	Ingestion/Inhalation Rate
IUR	Inhalation Unit Risk
LADD	Lifetime Average Daily Dose
LCR	Lifetime Cancer Risk
LOD	Limit of Detection
LOQ	Limit of Quantification
RfD	Reference Dose
