# Supplementary material for: Toxic Trace Element Concentration in Commercially Available Cigarettes in Poland, Europe
Source: Toxics. 2025 Dec 18;13(12):1088. doi: 10.3390/toxics13121088 (PMC12737748; doi:10.3390/toxics13121088)
Supplement: Supplementary file 1 [file toxics-13-01088-s001.zip › toxics-4031550-supplementary.pdf]

Table S1. Tobacco companies whose products were obtained for testing.

Tobacco company	Code assigned in the study	Number of samples
British American Tobacco	1	48
Imperial Tobacco	2	13
Japan Tobacco International	3	22
Philip Morris	4	36

Table S2. Concentration [mg/kg] of copper, lead, nickel, and cadmium in cigarettes available on the European market.

No.	Sample code	Brand	Tobacco company	Cu	Pb	Ni	Cd
1	C20/I/23/24	Winston	3	9.00	1.94	<LOQ	0.83
2	C21/I/23/24	LD	3	9.03	0.65	<LOQ	0.63
3	C22/I/23/24	Winston	3	7.32	1.55	<LOQ	0.78
4	C23/I/23/24	Winston	3	8.05	1.41	<LOQ	0.92
5	C24/I/23/24	Winston	3	7.86	0.16	<LOQ	0.74
6	C25/I/23/24	Winston	3	7.20	1.29	<LOQ	0.69
7	C26/I/23/24	LM	4	7.82	1.31	<LOQ	1.45
8	C27/I/23/24	LM	4	10.04	0.77	<LOQ	1.11
9	C28/I/23/24	Pall Mall	1	8.14	1.06	<LOQ	0.74
10	C29/I/23/24	LM	4	9.86	0.64	<LOQ	1.09
11	C30/I/23/24	LM	4	9.41	<LOQ	<LOQ	0.93
12	C31/I/23/24	Pall Mall	1	7.60	1.41	<LOQ	0.71
13	C32/I/23/24	LM	1	9.60	1.41	<LOQ	1.03
14	C33/I/23/24	Winston	3	6.70	1.14	<LOQ	0.80
15	C34/I/23/24	Marlboro	4	9.59	0.80	<LOQ	0.90
16	C35/I/23/24	Davidoff	2	10.91	1.44	<LOQ	0.97
17	C36/I/23/24	RGD	4	9.48	2.81	<LOQ	1.10
18	C37/I/23/24	LM	4	7.50	1.79	<LOQ	0.94
19	C38/I/23/24	LM	4	10.08	1.01	<LOQ	0.65
20	C39/I/23/24	Kent	1	10.18	0.20	2.36	0.73
21	C128 /I/22/23	Winston	3	10.93	<LOQ	2.33	0.46

22	C129 /I/22/23	Rothmans of London	1	13.47	0.35	2.30	0.50
23	C130 /I/22/23	LM	4	8.38	0.16	4.59	0.38
24	C131 /I/22/23	Pall Mall	1	10.74	0.38	9.55	3.71
25	C132 /I/22/23	Pall Mall	1	12.98	<LOQ	8.02	0.46
26	C133 /I/22/23	LM	4	11.25	<LOQ	<LOQ	1.07
27	C134 /I/22/23	LM	4	9.56	<LOQ	5.94	0.63
28	C135 /I/22/23	Camel	3	10.27	<LOQ	10.56	1.08
29	C136 /I/22/23	Marlboro	4	9.45	0.25	11.70	0.44
30	C137 /I/22/23	Pall Mall	1	10.70	<LOQ	6.04	0.54
31	C138 /I/22/23	Rothmans of London	1	9.80	0.34	<LOQ	1.38
32	C168 /I/22/23	Davidoff	2	11.34	<LOQ	2.49	0.57
33	C169 /I/22/23	Parker&Simpson	1	10.44	<LOQ	1.98	0.70
34	C170 /I/22/23	Pall Mall	1	11.43	<LOQ	<LOQ	0.56
35	C171 /I/22/23	LD	3	11.15	<LOQ	2.43	0.64
36	C172 /I/22/23	LM	4	12.72	<LOQ	2.50	0.92
37	C173 /I/22/23	Pall Mall	1	8.35	0.22	2.43	0.84
38	C174 /I/22/23	LM	4	10.20	<LOQ	1.71	0.84
39	C175 /I/22/23	Mocne	2	11.56	0.30	1.95	1.04
40	C176 /I/22/23	Rothmans of London	1	9.67	<LOQ	<LOQ	0.75
41	C177 /I/22/23	Parker&Simpson	1	8.83	<LOQ	2.96	0.72
42	C178 /I/22/23	Rothmans of London	1	10.01	0.17	2.54	0.48
43	C179 /I/22/23	Lucky Strike	1	11.20	0.29	2.19	0.62
44	C180 /I/22/23	Davidoff	2	13.19	0.31	<LOQ	0.72
45	C181 /I/22/23	LD	3	9.67	0.43	<LOQ	0.40
46	C182 /I/22/23	Rothmans of London	1	11.65	0.16	<LOQ	0.31
47	C183 /I/22/23	LM	4	8.95	<LOQ	2.22	0.41
48	C184 /I/22/23	Davidoff	2	13.88	<LOQ	1.94	0.55
49	C185 /I/22/23	Marlboro	4	10.31	0.62	2.58	0.35
50	C186 /I/22/23	Pall Mall	1	18.58	<LOQ	2.30	0.84
51	C187 /I/22/23	Parliament	4	13.13	<LOQ	10.50	0.76
52	C188 /I/22/23	Pall Mall	1	17.98	<LOQ	<LOQ	0.86
53	C 64/II/22/23	LM	4	13.44	<LOQ	2.82	0.74
54	C 65/II/22/23	Parker&Simpson	1	11.31	<LOQ	<LOQ	0.63
55	C 66/II/22/23	RGD	4	11.68	<LOQ	3.15	0.61
56	C 67/II/22/23	Winston	3	11.45	<LOQ	<LOQ	0.70
57	C 68/II/22/23	Winston	3	16.00	1633.27	<LOQ	0.63
58	C 69/II/22/23	Pall Mall	1	13.48	845.78	<LOQ	0.70

59	C 70/II/22/23	Camel	3	11.18	416.58	<LOQ	0.65
60	C 128/II/22/23	Rothmans of London	1	11.99	118.74	<LOQ	0.96
61	C 129/II/22/23	LM	4	15.88	139.01	<LOQ	3.69
62	C 130/II/22/23	Winston	3	11.55	68.30	<LOQ	0.65
63	C 131/II/22/23	Parker&Simpson	1	14.92	138.07	<LOQ	0.73
64	C 133/II/22/23	LM	4	11.95	36.64	6.00	1.02
65	C 134/II/22/23	LM	4	11.73	12.72	4.61	0.48
66	C 135/II/22/23	Pall Mall	1	12.73	15.47	7.01	1.02
67	C 136/II/22/23	Parker&Simpson	1	14.16	9.48	<LOQ	0.60
68	C 137/II/22/23	Pall Mall	1	10.29	7.36	<LOQ	0.53
69	C 138/II/22/23	Rothmans of London	1	10.91	7.98	<LOQ	0.52
70	C 139/II/22/23	LM	4	11.05	18.78	<LOQ	0.65
71	C 140/II/22/23	Kent	1	14.47	<LOQ	<LOQ	0.81
72	C 141/II/22/23	Kent	1	11.77	23.99	<LOQ	0.64
73	C 142/II/22/23	Winston	3	14.08	6.67	<LOQ	1.06
74	C 143/II/22/23	LM	4	17.81	14.47	1.67	0.91
75	C 144/II/22/23	LD	3	14.43	4.74	<LOQ	0.61
76	C 145/II/22/23	LM	4	15.07	4.07	<LOQ	0.75
77	C 146/II/22/23	Mocne	2	14.40	<LOQ	<LOQ	0.87
78	C 147/II/22/23	LM	4	11.91	10.24	<LOQ	0.88
79	C 148/II/22/23	RGD	4	14.01	0.37	<LOQ	0.93
80	C 149/II/22/23	LM	4	11.41	<LOQ	<LOQ	0.66
81	C 150/II/22/23	Rothmans of London	1	12.00	0.23	<LOQ	0.91
82	C 86/II/21/22	Rothmans of London	1	11.76	0.25	1.75	0.48
83	C 87/II/21/22	Winston	3	11.65	0.25	2.59	0.95
84	C 88/II/21/22	Rothmans of London	1	12.01	<LOQ	<LOQ	0.87
85	C 89/II/21/22	Rothmans of London	1	10.48	0.29	<LOQ	0.89
86	C 90/II/21/22	Rothmans of London	1	10.15	0.18	<LOQ	0.48
87	C 91/II/21/22	Rothmans of London	1	9.33	0.21	1.77	0.45
88	C 92/II/21/22	Rothmans of London	1	10.24	0.19	<LOQ	0.39
89	C 93/II/21/22	Rothmans of London	1	10.92	<LOQ	<LOQ	0.58
90	C 94/II/21/22	Rothmans of London	1	11.59	<LOQ	1.86	0.63
91	C 95/II/21/22	Rothmans of London	1	22.35	<LOQ	<LOQ	0.60
92	C 96/II/21/22	Winston	3	16.93	0.25	1.74	0.97
93	C 97/II/21/22	Chesterfield	4	13.45	0.28	1.95	0.99
94	C 98/II/21/22	Chesterfield	4	9.83	<LOQ	2.24	0.72
95	C 99/II/21/22	Rothmans of London	1	9.50	0.39	<LOQ	1.27

96	C 100/II/21/22	Mocne	2	9.46	0.16	15.44	0.94
97	C 101/II/21/22	Rothmans of London	1	5.88	<LOQ	2.92	1.20
98	C16/II/21/22	LD	3	11.74	<LOQ	3.18	0.84
99	C17/II/21/22	Parker&Simpson	1	10.34	<LOQ	2.87	1.06
100	C18/II/21/22	LM	4	13.92	0.33	2.56	0.85
101	C20/II/21/22	Mocne	2	94.63	0.35	3.91	3.06
102	C21/II/21/22	Chesterfield	4	23.06	0.17	6.42	1.09
103	C39/II/21/22	Davidoff	2	92.60	<LOQ	2.49	1.16
104	C40/II/21/22	Pall Mall	1	14.65	<LOQ	4.22	1.01
105	C41/II/21/22	LM	4	<LOQ	131.42	<LOQ	8.57
106	C43/II/21/22	Rothmans of London	1	<LOQ	29.10	<LOQ	2.84
107	C44/II/21/22	Rothmans of London	1	<LOQ	8.08	<LOQ	0.56
108	C46/II/21/22	Rothmans of London	1	<LOQ	4.60	<LOQ	2.86
109	C314 /II/17/18	West	2	<LOQ	15.75	<LOQ	2.87
110	C315 /II/17/18	LM	4	<LOQ	16.71	<LOQ	1.08
111	C316 /II/17/18	Davidoff	2	39.94	<LOQ	5.18	0.29
112	C317 /II/17/18	Davidoff	2	<LOQ	13.69	<LOQ	2.99
113	C319 /II/17/18	Rothmans of London	1	9.85	<LOQ	<LOQ	0.46
114	C320 /II/17/18	LM	4	9.77	0.28	<LOQ	0.67
115	C321 /II/17/18	Winston	3	6.27	0.18	<LOQ	1.50
116	C322 /II/17/18	Winston	3	9.00	0.21	4.12	1.09
117	C323 /II/17/18	Pall Mall	1	<LOQ	8.46	<LOQ	0.96
118	C324 /II/17/18	LM	4	<LOQ	11.23	<LOQ	0.62
119	C326 /II/17/18	Davidoff	2	3.36	7.54	5.48	4.05

Table S3. Average Daily Dose (ADD) [mg/kg] for inhalation exposure to copper, lead, nickel, and cadmium contained in the cigarettes tested.

No.	Sample code	1 cigarette per day				5 cigarettes per day				10 cigarettes per day				15 cigarettes per day				20 cigarettes per day			
		Cu	Pb	Ni	Cd	Cu	Pb	Ni	Cd	Cu	Pb	Ni	Cd	Cu	Pb	Ni	Cd	Cu	Pb	Ni	Cd
1	C20 /I/23/24	7.71E-05	1.66E-05	-	7.09E-06	3.86E-04	8.32E-05	-	3.55E-05	7.71E-04	1.66E-04	-	7.09E-05	1.16E-03	2.50E-04	-	1.06E-04	1.54E-03	3.33E-04	-	1.42E-04
2	C21 /I/23/24	7.74E-05	5.59E-06	-	5.39E-06	3.87E-04	2.80E-05	-	2.69E-05	7.74E-04	5.59E-05	-	5.39E-05	1.16E-03	8.39E-05	-	8.08E-05	1.55E-03	1.12E-04	-	1.08E-04
3	C22 /I/23/24	6.27E-05	1.33E-05	-	6.71E-06	3.14E-04	6.63E-05	-	3.36E-05	6.27E-04	1.33E-04	-	6.71E-05	9.41E-04	1.99E-04	-	1.01E-04	1.25E-03	2.65E-04	-	1.34E-04
4	C23 /I/23/24	6.90E-05	1.21E-05	-	7.89E-06	3.45E-04	6.06E-05	-	3.95E-05	6.90E-04	1.21E-04	-	7.89E-05	1.03E-03	1.82E-04	-	1.18E-04	1.38E-03	2.42E-04	-	1.58E-04
5	C24 /I/23/24	6.74E-05	1.41E-06	-	6.33E-06	3.37E-04	7.04E-06	-	3.16E-05	6.74E-04	1.41E-05	-	6.33E-05	1.01E-03	2.11E-05	-	9.49E-05	1.35E-03	2.82E-05	-	1.27E-04
6	C25 /I/23/24	6.17E-05	1.11E-05	-	5.91E-06	3.08E-04	5.53E-05	-	2.95E-05	6.17E-04	1.11E-04	-	5.91E-05	9.25E-04	1.66E-04	-	8.86E-05	1.23E-03	2.21E-04	-	1.18E-04
7	C26 /I/23/24	6.71E-05	1.13E-05	-	1.24E-05	3.35E-04	5.63E-05	-	6.21E-05	6.71E-04	1.13E-04	-	1.24E-04	1.01E-03	1.69E-04	-	1.86E-04	1.34E-03	2.25E-04	-	2.48E-04
8	C27 /I/23/24	8.61E-05	6.56E-06	-	9.49E-06	4.30E-04	3.28E-05	-	4.74E-05	8.61E-04	6.56E-05	-	9.49E-05	1.29E-03	9.84E-05	-	1.42E-04	1.72E-03	1.31E-04	-	1.90E-04
9	C28 /I/23/24	6.98E-05	9.11E-06	-	6.36E-06	3.49E-04	4.55E-05	-	3.18E-05	6.98E-04	9.11E-05	-	6.36E-05	1.05E-03	1.37E-04	-	9.55E-05	1.40E-03	1.82E-04	-	1.27E-04
10	C29 /I/23/24	8.45E-05	5.45E-06	-	9.32E-06	4.23E-04	2.73E-05	-	4.66E-05	8.45E-04	5.45E-05	-	9.32E-05	1.27E-03	8.18E-05	-	1.40E-04	1.69E-03	1.09E-04	-	1.86E-04
11	C30 /I/23/24	8.07E-05	-	-	7.99E-06	4.03E-04	-	-	4.00E-05	8.07E-04	-	-	7.99E-05	1.21E-03	-	-	1.20E-04	1.61E-03	-	-	1.60E-04
12	C31 /I/23/24	6.51E-05	1.21E-05	-	6.07E-06	3.26E-04	6.03E-05	-	3.04E-05	6.51E-04	1.21E-04	-	6.07E-05	9.77E-04	1.81E-04	-	9.11E-05	1.30E-03	2.41E-04	-	1.21E-04
13	C32 /I/23/24	8.23E-05	1.21E-05	-	8.86E-06	4.11E-04	6.06E-05	-	4.43E-05	8.23E-04	1.21E-04	-	8.86E-05	1.23E-03	1.82E-04	-	1.33E-04	1.65E-03	2.42E-04	-	1.77E-04
14	C33 /I/23/24	5.74E-05	9.80E-06	-	6.85E-06	2.87E-04	4.90E-05	-	3.42E-05	5.74E-04	9.80E-05	-	6.85E-05	8.61E-04	1.47E-04	-	1.03E-04	1.15E-03	1.96E-04	-	1.37E-04
15	C34 /I/23/24	8.22E-05	6.84E-06	-	7.73E-06	4.11E-04	3.42E-05	-	3.86E-05	8.22E-04	6.84E-05	-	7.73E-05	1.23E-03	1.03E-04	-	1.16E-04	1.64E-03	1.37E-04	-	1.55E-04
16	C36 /I/23/24	9.35E-05	1.23E-05	-	8.31E-06	4.68E-04	6.15E-05	-	4.16E-05	9.35E-04	1.23E-04	-	8.31E-05	1.40E-03	1.85E-04	-	1.25E-04	1.87E-03	2.46E-04	-	1.66E-04
17	C37 /I/23/24	8.12E-05	2.41E-05	-	9.42E-06	4.06E-04	1.20E-04	-	4.71E-05	8.12E-04	2.41E-04	-	9.42E-05	1.22E-03	3.61E-04	-	1.41E-04	1.62E-03	4.82E-04	-	1.88E-04

18	C38 /I/23/24	6.43E-05	1.53E-05	-	8.10E-06	3.21E-04	7.67E-05	-	4.05E-05	6.43E-04	1.53E-04	-	8.10E-05	9.64E-04	2.30E-04	-	1.21E-04	1.29E-03	3.07E-04	-	1.62E-04
19	C39 /I/23/24	8.64E-05	8.67E-06	-	5.56E-06	4.32E-04	4.33E-05	-	2.78E-05	8.64E-04	8.67E-05	-	5.56E-05	1.30E-03	1.30E-04	-	8.34E-05	1.73E-03	1.73E-04	-	1.11E-04
20	C128 /I/22/23	8.72E-05	1.69E-06	2.02E-05	6.26E-06	4.36E-04	8.45E-06	1.01E-04	3.13E-05	8.72E-04	1.69E-05	2.02E-04	6.26E-05	1.31E-03	2.54E-05	3.03E-04	9.39E-05	1.74E-03	3.38E-05	4.04E-04	1.25E-04
21	C129 /I/22/23	9.37E-05	-	2.00E-05	3.94E-06	4.69E-04	-	9.99E-05	1.97E-05	9.37E-04	-	2.00E-04	3.94E-05	1.41E-03	-	3.00E-04	5.91E-05	1.87E-03	-	4.00E-04	7.89E-05
22	C130 /I/22/23	1.15E-04	2.96E-06	1.97E-05	4.31E-06	5.77E-04	1.48E-05	9.85E-05	2.15E-05	1.15E-03	2.96E-05	1.97E-04	4.31E-05	1.73E-03	4.44E-05	2.95E-04	6.46E-05	2.31E-03	5.92E-05	3.94E-04	8.62E-05
23	C131 /I/22/23	7.18E-05	1.36E-06	3.94E-05	3.27E-06	3.59E-04	6.79E-06	1.97E-04	1.64E-05	7.18E-04	1.36E-05	3.94E-04	3.27E-05	1.08E-03	2.04E-05	5.90E-04	4.91E-05	1.44E-03	2.71E-05	7.87E-04	6.54E-05
24	C132 /I/22/23	9.21E-05	3.26E-06	8.19E-05	3.18E-05	4.60E-04	1.63E-05	4.09E-04	1.59E-04	9.21E-04	3.26E-05	8.19E-04	3.18E-04	1.38E-03	4.89E-05	1.23E-03	4.77E-04	1.84E-03	6.52E-05	1.64E-03	6.36E-04
25	C133 /I/22/23	1.11E-04	-	6.87E-05	3.95E-06	5.56E-04	-	3.44E-04	1.98E-05	1.11E-03	-	6.87E-04	3.95E-05	1.67E-03	-	1.03E-03	5.93E-05	2.23E-03	-	1.37E-03	7.90E-05
26	C134 /I/22/23	9.65E-05	-	-	9.21E-06	4.82E-04	-	-	4.61E-05	9.65E-04	-	-	9.21E-05	1.45E-03	-	-	1.38E-04	1.93E-03	-	-	1.84E-04
27	C135 /I/22/23	8.19E-05	-	5.09E-05	5.44E-06	4.10E-04	-	2.55E-04	2.72E-05	8.19E-04	-	5.09E-04	5.44E-05	1.23E-03	-	7.64E-04	8.16E-05	1.64E-03	-	1.02E-03	1.09E-04
28	C136 /I/22/23	8.81E-05	-	9.05E-05	9.29E-06	4.40E-04	-	4.52E-04	4.65E-05	8.81E-04	-	9.05E-04	9.29E-05	1.32E-03	-	1.36E-03	1.39E-04	1.76E-03	-	1.81E-03	1.86E-04
29	C137 /I/22/23	8.10E-05	2.17E-06	1.00E-04	3.74E-06	4.05E-04	1.08E-05	5.02E-04	1.87E-05	8.10E-04	2.17E-05	1.00E-03	3.74E-05	1.22E-03	3.25E-05	1.50E-03	5.61E-05	1.62E-03	4.34E-05	2.01E-03	7.48E-05
30	C138 /I/22/23	9.17E-05	-	5.18E-05	4.63E-06	4.58E-04	-	2.59E-04	2.31E-05	9.17E-04	-	5.18E-04	4.63E-05	1.38E-03	-	7.77E-04	6.94E-05	1.83E-03	-	1.04E-03	9.26E-05
31	C169 /I/22/23	8.40E-05	2.95E-06	-	1.18E-05	4.20E-04	1.47E-05	-	5.91E-05	8.40E-04	2.95E-05	-	1.18E-04	1.26E-03	4.42E-05	-	1.77E-04	1.68E-03	5.89E-05	-	2.36E-04
32	C170 /I/22/23	9.72E-05	-	2.13E-05	4.88E-06	4.86E-04	-	1.07E-04	2.44E-05	9.72E-04	-	2.13E-04	4.88E-05	1.46E-03	-	3.20E-04	7.33E-05	1.94E-03	-	4.26E-04	9.77E-05
33	C171 /I/22/23	8.95E-05	-	1.70E-05	6.01E-06	4.47E-04	-	8.49E-05	3.00E-05	8.95E-04	-	1.70E-04	6.01E-05	1.34E-03	-	2.55E-04	9.01E-05	1.79E-03	-	3.40E-04	1.20E-04
34	C172 /I/22/23	9.79E-05	-	-	4.82E-06	4.90E-04	-	-	2.41E-05	9.79E-04	-	-	4.82E-05	1.47E-03	-	-	7.23E-05	1.96E-03	-	-	9.64E-05
35	C173 /I/22/23	9.56E-05	-	2.08E-05	5.51E-06	4.78E-04	-	1.04E-04	2.75E-05	9.56E-04	-	2.08E-04	5.51E-05	1.43E-03	-	3.12E-04	8.26E-05	1.91E-03	-	4.16E-04	1.10E-04
36	C174 /I/22/23	1.09E-04	-	2.15E-05	7.90E-06	5.45E-04	-	1.07E-04	3.95E-05	1.09E-03	-	2.15E-04	7.90E-05	1.64E-03	-	3.22E-04	1.19E-04	2.18E-03	-	4.29E-04	1.58E-04

37	C176 /I/22/23	7.16E-05	1.92E-06	2.08E-05	7.18E-06	3.58E-04	9.62E-06	1.04E-04	3.59E-05	7.16E-04	1.92E-05	2.08E-04	7.18E-05	1.07E-03	2.89E-05	3.12E-04	1.08E-04	1.43E-03	3.85E-05	4.17E-04	1.44E-04
38	C177 /I/22/23	8.74E-05	-	1.47E-05	7.22E-06	4.37E-04	-	7.33E-05	3.61E-05	8.74E-04	-	1.47E-04	7.22E-05	1.31E-03	-	2.20E-04	1.08E-04	1.75E-03	-	2.93E-04	1.44E-04
39	C178 /I/22/23	9.91E-05	2.57E-06	1.67E-05	8.92E-06	4.95E-04	1.28E-05	8.34E-05	4.46E-05	9.91E-04	2.57E-05	1.67E-04	8.92E-05	1.49E-03	3.85E-05	2.50E-04	1.34E-04	1.98E-03	5.13E-05	3.34E-04	1.78E-04
40	C179 /I/22/23	8.29E-05	-	-	6.43E-06	4.14E-04	-	-	3.21E-05	8.29E-04	-	-	6.43E-05	1.24E-03	-	-	9.64E-05	1.66E-03	-	-	1.29E-04
41	C180 /I/22/23	7.57E-05	-	2.53E-05	6.15E-06	3.78E-04	--	1.27E-04	3.08E-05	7.57E-04		2.53E-04	6.15E-05	1.14E-03	-	3.80E-04	9.23E-05	1.51E-03	-	5.07E-04	1.23E-04
42	C181 /I/22/23	8.58E-05	1.42E-06	2.18E-05	4.10E-06	4.29E-04	7.11E-06	1.09E-04	2.05E-05	8.58E-04	1.42E-05	2.18E-04	4.10E-05	1.29E-03	2.13E-05	3.27E-04	6.15E-05	1.72E-03	2.84E-05	4.35E-04	8.19E-05
43	C182 /I/22/23	9.60E-05	2.47E-06	1.88E-05	5.29E-06	4.80E-04	1.24E-05	9.39E-05	2.65E-05	9.60E-04	2.47E-05	1.88E-04	5.29E-05	1.44E-03	3.71E-05	2.82E-04	7.94E-05	1.92E-03	4.94E-05	3.76E-04	1.06E-04
44	C183 /I/22/23	1.13E-04	2.64E-06	-	6.14E-06	5.65E-04	1.32E-05	-	3.07E-05	1.13E-03	2.64E-05	-	6.14E-05	1.70E-03	3.97E-05	-	9.22E-05	2.26E-03	5.29E-05	-	1.23E-04
45	C184 /I/22/23	8.29E-05	3.66E-06	-	3.47E-06	4.14E-04	1.83E-05	-	1.73E-05	8.29E-04	3.66E-05	-	3.47E-05	1.24E-03	5.49E-05	-	5.20E-05	1.66E-03	7.32E-05	-	6.94E-05
46	C185 /I/22/23	9.98E-05	1.39E-06	-	2.66E-06	4.99E-04	6.97E-06	-	1.33E-05	9.98E-04	1.39E-05	-	2.66E-05	1.50E-03	2.09E-05	-	3.99E-05	2.00E-03	2.79E-05	-	5.32E-05
47	C186 /I/22/23	7.67E-05	-	1.90E-05	3.54E-06	3.84E-04	-	9.51E-05	1.77E-05	7.67E-04	-	1.90E-04	3.54E-05	1.15E-03	-	2.85E-04	5.32E-05	1.53E-03	-	3.80E-04	7.09E-05
48	C187 /I/22/23	1.19E-04	-	1.66E-05	4.69E-06	5.95E-04	-	8.31E-05	2.34E-05	1.19E-03	-	1.66E-04	4.69E-05	1.78E-03	-	2.49E-04	7.03E-05	2.38E-03	-	3.32E-04	9.38E-05
49	C188 /I/22/23	8.83E-05	5.34E-06	2.21E-05	3.01E-06	4.42E-04	2.67E-05	1.10E-04	1.50E-05	8.83E-04	5.34E-05	2.21E-04	3.01E-05	1.33E-03	8.00E-05	3.31E-04	4.51E-05	1.77E-03	1.07E-04	4.42E-04	6.02E-05
50	C64 /II/22/23	1.59E-04	-	1.97E-05	7.17E-06	7.96E-04	-	9.86E-05	3.59E-05	1.59E-03	-	1.97E-04	7.17E-05	2.39E-03	-	2.96E-04	1.08E-04	3.19E-03	-	3.94E-04	1.43E-04
51	C65 /II/22/23	1.13E-04	-	9.00E-05	6.50E-06	5.63E-04	-	4.50E-04	3.25E-05	1.13E-03	-	9.00E-04	6.50E-05	1.69E-03	-	1.35E-03	9.74E-05	2.25E-03	-	1.80E-03	1.30E-04
52	C66 /II/22/23	1.54E-04	-	-	7.39E-06	7.71E-04	-	-	3.70E-05	1.54E-03	-	-	7.39E-05	2.31E-03	-	-	1.11E-04	3.08E-03	-	-	1.48E-04
53	C67 /II/22/23	1.15E-04	-	2.42E-05	6.35E-06	5.76E-04	-	1.21E-04	3.18E-05	1.15E-03	-	2.42E-04	6.35E-05	1.73E-03	-	3.63E-04	9.53E-05	2.30E-03	-	4.83E-04	1.27E-04
54	C68 /II/22/23	9.70E-05	-	-	5.43E-06	4.85E-04	-	-	2.71E-05	9.70E-04	-	-	5.43E-05	1.45E-03	-	-	8.14E-05	1.94E-03	-	-	1.09E-04
55	C69 /II/22/23	1.00E-04	-	2.70E-05	5.24E-06	5.01E-04	-	1.35E-04	2.62E-05	1.00E-03	-	2.70E-04	5.24E-05	1.50E-03	-	4.05E-04	7.86E-05	2.00E-03	-	5.40E-04	1.05E-04

56	C70 /II/22/23	9.81E-05	-	-	6.03E-06	4.91E-04	-	-	3.01E-05	9.81E-04	-	-	6.03E-05	1.47E-03	-	-	9.04E-05	1.96E-03	-	-	1.21E-04
57	C128 /II/22/23	1.37E-04	1.40E-02	-	5.43E-06	6.86E-04	7.00E-02	-	2.72E-05	1.37E-03	1.40E-01	-	5.43E-05	2.06E-03	2.10E-01	-	8.15E-05	2.74E-03	2.80E-01	-	1.09E-04
58	C129 /II/22/23	1.16E-04	7.25E-03	-	5.97E-06	5.78E-04	3.62E-02	-	2.99E-05	1.16E-03	7.25E-02	-	5.97E-05	1.73E-03	1.09E-01	-	8.96E-05	2.31E-03	1.45E-01	-	1.19E-04
59	C130 /II/22/23	9.58E-05	3.57E-03	-	5.54E-06	4.79E-04	1.79E-02	-	2.77E-05	9.58E-04	3.57E-02	-	5.54E-05	1.44E-03	5.36E-02	-	8.31E-05	1.92E-03	7.14E-02	-	1.11E-04
60	C131 /II/22/23	1.03E-04	1.02E-03	-	8.24E-06	5.14E-04	5.09E-03	-	4.12E-05	1.03E-03	1.02E-02	-	8.24E-05	1.54E-03	1.53E-02	-	1.24E-04	2.06E-03	2.04E-02	-	1.65E-04
61	C133 /II/22/23	9.90E-05	5.85E-04	-	5.55E-06	4.95E-04	2.93E-03	-	2.77E-05	9.90E-04	5.85E-03	-	5.55E-05	1.49E-03	8.78E-03	-	8.32E-05	1.98E-03	1.17E-02	-	1.11E-04
62	C134 /II/22/23	1.28E-04	1.18E-03	-	6.25E-06	6.39E-04	5.92E-03	-	3.13E-05	1.28E-03	1.18E-02	-	6.25E-05	1.92E-03	1.78E-02	-	9.38E-05	2.56E-03	2.37E-02	-	1.25E-04
63	C135 /II/22/23	9.84E-05	5.05E-04	-	4.86E-06	4.92E-04	2.53E-03	-	2.43E-05	9.84E-04	5.05E-03	-	4.86E-05	1.48E-03	7.58E-03	-	7.29E-05	1.97E-03	1.01E-02	-	9.71E-05
64	C136 /II/22/23	1.02E-04	3.14E-04	5.14E-05	8.71E-06	5.12E-04	1.57E-03	2.57E-04	4.36E-05	1.02E-03	3.14E-03	5.14E-04	8.71E-05	1.54E-03	4.71E-03	7.71E-04	1.31E-04	2.05E-03	6.28E-03	1.03E-03	1.74E-04
65	C137 /II/22/23	1.01E-04	1.09E-04	3.95E-05	4.13E-06	5.03E-04	5.45E-04	1.98E-04	2.06E-05	1.01E-03	1.09E-03	3.95E-04	4.13E-05	1.51E-03	1.64E-03	5.93E-04	6.19E-05	2.01E-03	2.18E-03	7.91E-04	8.25E-05
66	C138 /II/22/23	1.09E-04	1.33E-04	6.01E-05	8.71E-06	5.46E-04	6.63E-04	3.00E-04	4.35E-05	1.09E-03	1.33E-03	6.01E-04	8.71E-05	1.64E-03	1.99E-03	9.01E-04	1.31E-04	2.18E-03	2.65E-03	1.20E-03	1.74E-04
67	C139 /II/22/23	1.21E-04	8.13E-05	-	5.11E-06	6.07E-04	4.06E-04	-	2.56E-05	1.21E-03	8.13E-04	-	5.11E-05	1.82E-03	1.22E-03	-	7.67E-05	2.43E-03	1.63E-03	-	1.02E-04
68	C140 /II/22/23	8.82E-05	6.31E-05	-	4.53E-06	4.41E-04	3.15E-04	-	2.27E-05	8.82E-04	6.31E-04	-	4.53E-05	1.32E-03	9.46E-04	-	6.80E-05	1.76E-03	1.26E-03	-	9.06E-05
69	C141 /II/22/23	9.35E-05	6.84E-05	-	4.44E-06	4.68E-04	3.42E-04	-	2.22E-05	9.35E-04	6.84E-04	-	4.44E-05	1.40E-03	1.03E-03	-	6.65E-05	1.87E-03	1.37E-03	-	8.87E-05
70	C142 /II/22/23	9.47E-05	1.61E-04	-	5.53E-06	4.73E-04	8.05E-04	-	2.77E-05	9.47E-04	1.61E-03	-	5.53E-05	1.42E-03	2.41E-03	-	8.30E-05	1.89E-03	3.22E-03	-	1.11E-04
71	C143 /II/22/23	1.24E-04	-	-	6.94E-06	6.20E-04	-	-	3.47E-05	1.24E-03	-	-	6.94E-05	1.86E-03	-	-	1.04E-04	2.48E-03	-	-	1.39E-04
72	C144 /II/22/23	1.01E-04	2.06E-04	-	5.50E-06	5.04E-04	1.03E-03	-	2.75E-05	1.01E-03	2.06E-03	-	5.50E-05	1.51E-03	3.08E-03	-	8.25E-05	2.02E-03	4.11E-03	-	1.10E-04
73	C145 /II/22/23	1.21E-04	5.72E-05	-	9.06E-06	6.03E-04	2.86E-04	-	4.53E-05	1.21E-03	5.72E-04	-	9.06E-05	1.81E-03	8.57E-04	-	1.36E-04	2.41E-03	1.14E-03	-	1.81E-04
74	C146 /II/22/23	1.53E-04	1.24E-04	1.43E-05	7.79E-06	7.63E-04	6.20E-04	7.16E-05	3.89E-05	1.53E-03	1.24E-03	1.43E-04	7.79E-05	2.29E-03	1.86E-03	2.15E-04	1.17E-04	3.05E-03	2.48E-03	2.86E-04	1.56E-04

75	C147 /II/22/23	1.24E-04	4.06E-05	-	5.24E-06	6.18E-04	2.03E-04	-	2.62E-05	1.24E-03	4.06E-04	-	5.24E-05	1.85E-03	6.10E-04	-	7.86E-05	2.47E-03	8.13E-04	-	1.05E-04
76	C148 /II/22/23	1.29E-04	3.48E-05	-	6.44E-06	6.46E-04	1.74E-04	-	3.22E-05	1.29E-03	3.48E-04	-	6.44E-05	1.94E-03	5.23E-04	-	9.66E-05	2.58E-03	6.97E-04	-	1.29E-04
77	C149 /II/22/23	1.23E-04	-	-	7.49E-06	6.17E-04	-	-	3.75E-05	1.23E-03	-	-	7.49E-05	1.85E-03	-	-	1.12E-04	2.47E-03	-	-	1.50E-04
78	C15 /II/22/23	1.02E-04	8.78E-05	-	7.56E-06	5.10E-04	4.39E-04	-	3.78E-05	1.02E-03	8.78E-04	-	7.56E-05	1.53E-03	1.32E-03	-	1.13E-04	2.04E-03	1.76E-03	-	1.51E-04
79	C86 /II/21/22	1.20E-04	3.14E-06	-	7.94E-06	6.00E-04	1.57E-05	-	3.97E-05	1.20E-03	3.14E-05	-	7.94E-05	1.80E-03	4.71E-05	-	1.19E-04	2.40E-03	6.27E-05	-	1.59E-04
80	C87 /II/21/22	9.78E-05	-	-	5.64E-06	4.89E-04	-	-	2.82E-05	9.78E-04	-	-	5.64E-05	1.47E-03	-	-	8.47E-05	1.96E-03	-	-	1.13E-04
81	C88 /II/21/22	1.03E-04	1.96E-06	-	7.80E-06	5.14E-04	9.78E-06	-	3.90E-05	1.03E-03	1.96E-05	-	7.80E-05	1.54E-03	2.93E-05	-	1.17E-04	2.06E-03	3.91E-05	-	1.56E-04
82	C89 /II/21/22	1.01E-04	2.14E-06	1.50E-05	4.08E-06	5.04E-04	1.07E-05	7.52E-05	2.04E-05	1.01E-03	2.14E-05	1.50E-04	4.08E-05	1.51E-03	3.21E-05	2.26E-04	6.12E-05	2.02E-03	4.28E-05	3.01E-04	8.16E-05
83	C90 /II/21/22	9.99E-05	2.13E-06	2.22E-05	8.18E-06	4.99E-04	1.07E-05	1.11E-04	4.09E-05	9.99E-04	2.13E-05	2.22E-04	8.18E-05	1.50E-03	3.20E-05	3.33E-04	1.23E-04	2.00E-03	4.27E-05	4.44E-04	1.64E-04
84	C91 /II/21/22	1.03E-04	-	-	7.50E-06	5.15E-04	-	-	3.75E-05	1.03E-03	-	-	7.50E-05	1.54E-03	-	-	1.12E-04	2.06E-03	-	-	1.50E-04
85	C92 /II/21/22	8.99E-05	2.51E-06	-	7.65E-06	4.49E-04	1.25E-05	-	3.83E-05	8.99E-04	2.51E-05	-	7.65E-05	1.35E-03	3.76E-05	-	1.15E-04	1.80E-03	5.02E-05	-	1.53E-04
86	C93 /II/21/22	8.70E-05	1.58E-06	-	4.14E-06	4.35E-04	7.90E-06	-	2.07E-05	8.70E-04	1.58E-05	-	4.14E-05	1.31E-03	2.37E-05	-	6.21E-05	1.74E-03	3.16E-05	-	8.28E-05
87	C94 /II/21/22	8.00E-05	1.84E-06	1.52E-05	3.88E-06	4.00E-04	9.20E-06	7.59E-05	1.94E-05	8.00E-04	1.84E-05	1.52E-04	3.88E-05	1.20E-03	2.76E-05	2.28E-04	5.82E-05	1.60E-03	3.68E-05	3.04E-04	7.76E-05
88	C95 /II/21/22	8.78E-05	1.66E-06	-	3.31E-06	4.39E-04	8.29E-06	v	1.65E-05	8.78E-04	1.66E-05	-	3.31E-05	1.32E-03	2.49E-05	-	4.96E-05	1.76E-03	3.31E-05	-	6.62E-05
89	C96 /II/21/22	9.36E-05	-	-	4.96E-06	4.68E-04	-		2.48E-05	9.36E-04	-	-	4.96E-05	1.40E-03	-	-	7.44E-05	1.87E-03	-	-	9.92E-05
90	C97 /II/21/22	9.94E-05	-	1.60E-05	5.36E-06	4.97E-04	-	7.98E-05	2.68E-05	9.94E-04	-	1.60E-04	5.36E-05	1.49E-03	-	2.39E-04	8.04E-05	1.99E-03	-	3.19E-04	1.07E-04
91	C98 /II/21/22	1.92E-04	-	-	5.12E-06	9.58E-04	-	-	2.56E-05	1.92E-03	-	-	5.12E-05	2.87E-03	-	-	7.69E-05	3.83E-03	-	-	1.02E-04
92	C99 /II/21/22	1.45E-04	2.16E-06	1.49E-05	8.35E-06	7.25E-04	1.08E-05	7.46E-05	4.17E-05	1.45E-03	2.16E-05	1.49E-04	8.35E-05	2.18E-03	3.24E-05	2.24E-04	1.25E-04	2.90E-03	4.32E-05	2.98E-04	1.67E-04
93	C101 /II/21/22	1.15E-04	2.38E-06	1.67E-05	8.47E-06	5.77E-04	1.19E-05	8.37E-05	4.24E-05	1.15E-03	2.38E-05	1.67E-04	8.47E-05	1.73E-03	3.57E-05	2.51E-04	1.27E-04	2.31E-03	4.76E-05	3.35E-04	1.69E-04

94	C16 /II/21/22	8.42E-05	-	1.92E-05	6.21E-06	4.21E-04	-	9.58E-05	3.11E-05	8.42E-04	-	1.92E-04	6.21E-05	1.26E-03	-	2.87E-04	9.32E-05	1.68E-03	-	3.83E-04	1.24E-04
95	C17 /II/21/22	8.14E-05	3.33E-06	-	1.09E-05	4.07E-04	1.67E-05	-	5.43E-05	8.14E-04	3.33E-05	-	1.09E-04	1.22E-03	5.00E-05	-	1.63E-04	1.63E-03	6.67E-05	-	2.17E-04
96	C18 /II/21/22	8.10E-05	1.34E-06	1.32E-04	8.09E-06	4.05E-04	6.70E-06	6.62E-04	4.04E-05	8.10E-04	1.34E-05	1.32E-03	8.09E-05	1.22E-03	2.01E-05	1.98E-03	1.21E-04	1.62E-03	2.68E-05	2.65E-03	1.62E-04
97	C21 /II/21/22	5.04E-05	-	2.51E-05	1.03E-05	2.52E-04	-	1.25E-04	5.15E-05	5.04E-04	-	2.51E-04	1.03E-04	7.56E-04	-	3.76E-04	1.54E-04	1.01E-03	-	5.01E-04	2.06E-04
98	C39 /II/21/22	1.01E-04	-	2.73E-05	7.21E-06	5.03E-04	-	1.36E-04	3.60E-05	1.01E-03	-	2.73E-04	7.21E-05	1.51E-03	-	4.09E-04	1.08E-04	2.01E-03	-	5.46E-04	1.44E-04
99	C40 /II/21/22	8.86E-05	-	2.46E-05	9.09E-06	4.43E-04	-	1.23E-04	4.54E-05	8.86E-04	-	2.46E-04	9.09E-05	1.33E-03	-	3.69E-04	1.36E-04	1.77E-03	-	4.92E-04	1.82E-04
10	C41 /II/21/22	1.19E-04	2.83E-06	2.19E-05	7.30E-06	5.97E-04	1.41E-05	1.10E-04	3.65E-05	1.19E-03	2.83E-05	2.19E-04	7.30E-05	1.79E-03	4.24E-05	3.29E-04	1.10E-04	2.39E-03	5.66E-05	4.39E-04	1.46E-04
101	C43 /II/21/22	1.98E-04	1.43E-06	5.50E-05	9.35E-06	9.88E-04	7.16E-06	2.75E-04	4.68E-05	1.98E-03	1.43E-05	5.50E-04	9.35E-05	2.96E-03	2.15E-05	8.25E-04	1.40E-04	3.95E-03	2.87E-05	1.10E-03	1.87E-04
102	C44 /II/21/22	7.94E-04	-	2.13E-05	9.95E-06	3.97E-03	-	1.07E-04	4.98E-05	7.94E-03	-	2.13E-04	9.95E-05	1.19E-02	-	3.20E-04	1.49E-04	1.59E-02	v	4.27E-04	1.99E-04
103	C46 /II/21/22	1.26E-04	-	3.62E-05	8.68E-06	6.28E-04	-	1.81E-04	4.34E-05	1.26E-03	-	3.62E-04	8.68E-05	1.88E-03	-	5.42E-04	1.30E-04	2.51E-03		7.23E-04	1.74E-04
104	C314 /II/17/18	-	1.13E-03	-	7.35E-05	-	5.63E-03	-	3.67E-04	-	1.13E-02	-	7.35E-04	-	1.69E-02	-	1.10E-03	-	2.25E-02	-	1.47E-03
105	C315 /II/17/18	-	1.23E-04	-	4.29E-05	-	6.15E-04	-	2.15E-04	-	1.23E-03	-	4.29E-04	-	1.85E-03	-	6.44E-04	-	2.46E-03	-	8.59E-04
106	C319 /II/17/18	-	6.93E-05	-	4.80E-06	-	3.46E-04	-	2.40E-05	-	6.93E-04	-	4.80E-05	-	1.04E-03	-	7.20E-05	-	1.39E-03	-	9.60E-05
107	C320 /II/17/18	-	3.94E-05	-	2.45E-05	-	1.97E-04	-	1.23E-04	-	3.94E-04	-	2.45E-04	-	5.91E-04	-	3.68E-04	-	7.89E-04	-	4.90E-04
108	C321 /II/17/18	-	1.35E-04	-	2.46E-05	-	6.75E-04	-	1.23E-04	-	1.35E-03	-	2.46E-04	-	2.03E-03	-	3.69E-04	-	2.70E-03	-	4.92E-04
109	C322 /II/17/18	-	1.43E-04	-	9.26E-06	-	7.16E-04	-	4.63E-05	-	1.43E-03	-	9.26E-05	-	2.15E-03	-	1.39E-04	-	2.86E-03	-	1.85E-04
110	C323 /II/17/18	3.42E-04	-	4.44E-05	2.49E-06	1.71E-03	-	2.22E-04	1.24E-05	3.42E-03	-	4.44E-04	2.49E-05	5.14E-03	-	6.66E-04	3.73E-05	6.85E-03	-	8.88E-04	4.97E-05
111	C324 /II/17/18	-	1.17E-04	-	2.56E-05	-	5.87E-04	-	1.28E-04	-	1.17E-03	-	2.56E-04	-	1.76E-03	-	3.84E-04	-	2.35E-03	-	5.13E-04

112	C35 /I/23/24	7.53E-05	1.59E-05	-	8.40E-06	3.76E-04	7.94E-05	-	4.20E-05	7.53E-04	1.59E-04	-	8.40E-05	1.13E-03	2.38E-04	-	1.26E-04	1.51E-03	3.18E-04	-	1.68E-04
113	C168 /I/22/23	8.44E-05	-	-	3.98E-06	4.22E-04	-	-	1.99E-05	8.44E-04	-	-	3.98E-05	1.27E-03		-	5.97E-05	1.69E-03		-	7.96E-05
114	C175 /I/22/23	8.38E-05	2.39E-06	-	5.75E-06	4.19E-04	1.20E-05	-	2.87E-05	8.38E-04	2.39E-05	-	5.75E-05	1.26E-03	3.59E-05	-	8.62E-05	1.68E-03	4.79E-05	-	1.15E-04
115	C100 /II/21/22	5.38E-05	1.58E-06	-	1.28E-05	2.69E-04	7.91E-06	-	6.42E-05	5.38E-04	1.58E-05	-	1.28E-04	8.06E-04	2.37E-05	-	1.93E-04	1.08E-03	3.16E-05	-	2.57E-04
116	C20 /II/21/22	7.71E-05	1.84E-06	3.53E-05	9.31E-06	3.86E-04	9.19E-06	1.77E-04	4.65E-05	7.71E-04	1.84E-05	3.53E-04	9.31E-05	1.16E-03	2.76E-05	5.30E-04	1.40E-04	1.54E-03	3.68E-05	7.07E-04	1.86E-04
117	C316 /II/17/18	-	7.25E-05	-	8.23E-06	-	3.63E-04	-	4.11E-05	-	7.25E-04	-	8.23E-05	-	1.09E-03	-	1.23E-04	-	1.45E-03	-	1.65E-04
118	C317 /II/17/18	-	9.63E-05	-	5.31E-06	-	4.81E-04	-	2.66E-05	-	9.63E-04	-	5.31E-05	-	1.44E-03	-	7.97E-05	-	1.93E-03	-	1.06E-04
119	C326 /II/17/18	2.88E-05	6.46E-05	4.70E-05	3.47E-05	1.44E-04	3.23E-04	2.35E-04	1.74E-04	2.88E-04	6.46E-04	4.70E-04	3.47E-04	4.32E-04	9.69E-04	7.05E-04	5.21E-04	5.76E-04	1.29E-03	9.40E-04	6.94E-04

Table S4. Hazard Quotient (HQ) for inhalation exposure to copper, lead, nickel, and cadmium contained in the cigarettes tested.

No.	Sample code	1 cigarette per day				5 cigarettes per day				10 cigarettes per day				15 cigarettes per day				20 cigarettes per day			
		**Cu**	**Pb**	**Ni**	**Cd**	**Cu**	**Pb**	**Ni**	**Cd**	**Cu**	**Pb**	**Ni**	**Cd**	**Cu**	**Pb**	**Ni**	**Cd**	**Cu**	**Pb**	**Ni**	**Cd**
1	C20 /I/23/24	1.93E-03	1.11E-01	-	7.09E-01	9.64E-03	5.55E-01	-	**3.55E+00**	1.93E-02	1.11E+00	-	**7.09E+00**	2.89E-02	**1.66E+00**	-	**1.06E+01**	3.86E-02	**2.22E+00**	-	**1.42E+01**
2	C21 /I/23/24	1.94E-03	3.73E-02	-	5.39E-01	9.68E-03	1.86E-01	-	**2.69E+00**	1.94E-02	3.73E-01	-	**5.39E+00**	2.90E-02	5.59E-01	-	**8.08E+00**	3.87E-02	7.46E-01	-	**1.08E+01**
3	C22 /I/23/24	1.57E-03	8.84E-02	-	6.71E-01	7.84E-03	4.42E-01	-	**3.36E+00**	1.57E-02	8.84E-01	-	**6.71E+00**	2.35E-02	**1.33E+00**	-	**1.01E+01**	3.14E-02	**1.77E+00**	-	**1.34E+01**
4	C23 /I/23/24	1.72E-03	8.08E-02	-	7.89E-01	8.62E-03	4.04E-01	-	**3.95E+00**	1.72E-02	8.08E-01	-	**7.89E+00**	2.59E-02	**1.21E+00**	-	**1.18E+01**	3.45E-02	**1.62E+00**	-	**1.58E+01**
5	C24 /I/23/24	1.69E-03	9.39E-03	-	6.33E-01	8.43E-03	4.70E-02	-	**3.16E+00**	1.69E-02	9.39E-02	-	**6.33E+00**	2.53E-02	1.41E-01	-	**9.49E+00**	3.37E-02	1.88E-01	-	**1.27E+01**
6	C25 /I/23/24	1.54E-03	7.38E-02	-	5.91E-01	7.71E-03	3.69E-01	-	**2.95E+00**	1.54E-02	7.38E-01	-	**5.91E+00**	2.31E-02	**1.11E+00**	-	**8.86E+00**	3.08E-02	**1.48E+00**	-	**1.18E+01**
7	C26 /I/23/24	1.68E-03	7.50E-02	-	**1.24E+00**	8.38E-03	3.75E-01	-	**6.21E+00**	1.68E-02	7.50E-01	-	**1.24E+01**	2.51E-02	**1.13E+00**	-	**1.86E+01**	3.35E-02	**1.50E+00**	-	**2.48E+01**
8	C27 /I/23/24	2.15E-03	4.37E-02	-	9.49E-01	1.08E-02	2.19E-01	-	**4.74E+00**	2.15E-02	4.37E-01	-	**9.49E+00**	3.23E-02	6.56E-01	-	**1.42E+01**	4.30E-02	8.75E-01	-	**1.90E+01**
9	C28 /I/23/24	1.74E-03	6.07E-02	-	6.36E-01	8.72E-03	3.04E-01	-	**3.18E+00**	1.74E-02	6.07E-01	-	**6.36E+00**	2.62E-02	9.11E-01	-	**9.55E+00**	3.49E-02	**1.21E+00**	-	**1.27E+01**
10	C29 /I/23/24	2.11E-03	3.64E-02	-	9.32E-01	1.06E-02	1.82E-01	-	**4.66E+00**	2.11E-02	3.64E-01	-	**9.32E+00**	3.17E-02	5.45E-01	-	**1.40E+01**	4.23E-02	7.27E-01	-	**1.86E+01**
11	C30 /I/23/24	2.02E-03	-	-	7.99E-01	1.01E-02	-	-	**4.00E+00**	2.02E-02	-	-	**7.99E+00**	3.03E-02	-	-	**1.20E+01**	4.03E-02	-	-	**1.60E+01**
12	C31 /I/23/24	1.63E-03	8.03E-02	-	6.07E-01	8.14E-03	4.02E-01	-	**3.04E+00**	1.63E-02	8.03E-01	-	**6.07E+00**	2.44E-02	**1.21E+00**	-	**9.11E+00**	3.26E-02	**1.61E+00**	-	**1.21E+01**
13	C32 /I/23/24	2.06E-03	8.08E-02	-	8.86E-01	1.03E-02	4.04E-01	-	**4.43E+00**	2.06E-02	8.08E-01	-	**8.86E+00**	3.09E-02	**1.21E+00**	-	**1.33E+01**	4.11E-02	**1.62E+00**	-	**1.77E+01**
14	C33 /I/23/24	1.44E-03	6.53E-02	-	6.85E-01	7.18E-03	3.27E-01	-	**3.42E+00**	1.44E-02	6.53E-01	-	**6.85E+00**	2.15E-02	9.80E-01	-	**1.03E+01**	2.87E-02	**1.31E+00**	-	**1.37E+01**
15	C34 /I/23/24	2.05E-03	4.56E-02	-	7.73E-01	1.03E-02	2.28E-01	-	**3.86E+00**	2.05E-02	4.56E-01	-	**7.73E+00**	3.08E-02	6.84E-01	-	**1.16E+01**	4.11E-02	9.12E-01	-	**1.55E+01**
16	C36 /I/23/24	2.34E-03	8.20E-02	-	8.31E-01	1.17E-02	4.10E-01	-	**4.16E+00**	2.34E-02	8.20E-01	-	**8.31E+00**	3.51E-02	**1.23E+00**	-	**1.25E+01**	4.68E-02	**1.64E+00**	-	**1.66E+01**
17	C37 /I/23/24	2.03E-03	1.61E-01	-	9.42E-01	1.02E-02	8.03E-01	-	**4.71E+00**	2.03E-02	1.61E+00	-	**9.42E+00**	3.05E-02	**2.41E+00**	-	**1.41E+01**	4.06E-02	**3.21E+00**	-	**1.88E+01**

18	C38 /I/23/24	1.61E-03	1.02E-01	-	8.10E-01	8.04E-03	5.12E-01	-	**4.05E+00**	1.61E-02	1.02E+00	-	**8.10E+00**	2.41E-02	**1.53E+00**	-	**1.21E+01**	3.21E-02	**2.05E+00**	-	**1.62E+01**
19	C39 /I/23/24	2.16E-03	5.78E-02	-	5.56E-01	1.08E-02	2.89E-01	-	**2.78E+00**	2.16E-02	5.78E-01	-	**5.56E+00**	3.24E-02	8.67E-01	-	**8.34E+00**	4.32E-02	**1.16E+00**	-	**1.11E+01**
20	C128 /I/22/23	2.18E-03	1.13E-02	2.25E-01	6.26E-01	1.09E-02	5.64E-02	**1.12E+00**	**3.13E+00**	2.18E-02	1.13E-01	**2.25E+00**	**6.26E+00**	3.27E-02	1.69E-01	**3.37E+00**	**9.39E+00**	4.36E-02	2.25E-01	**4.49E+00**	**1.25E+01**
21	C129 /I/22/23	2.34E-03	-	2.22E-01	3.94E-01	1.17E-02	-	**1.11E+00**	**1.97E+00**	2.34E-02	-	**2.22E+00**	**3.94E+00**	3.51E-02	-	**3.33E+00**	**5.91E+00**	4.69E-02	-	**4.44E+00**	**7.89E+00**
22	C130 /I/22/23	2.89E-03	1.97E-02	2.19E-01	4.31E-01	1.44E-02	9.87E-02	**1.09E+00**	**2.15E+00**	2.89E-02	1.97E-01	**2.19E+00**	**4.31E+00**	4.33E-02	2.96E-01	**3.28E+00**	**6.46E+00**	5.77E-02	3.95E-01	**4.38E+00**	**8.62E+00**
23	C131 /I/22/23	1.80E-03	9.05E-03	4.37E-01	3.27E-01	8.98E-03	4.52E-02	**2.19E+00**	**1.64E+00**	1.80E-02	9.05E-02	**4.37E+00**	**3.27E+00**	2.69E-02	1.36E-01	**6.56E+00**	**4.91E+00**	3.59E-02	1.81E-01	**8.75E+00**	**6.54E+00**
24	C132 /I/22/23	2.30E-03	2.17E-02	9.10E-01	**3.18E+00**	1.15E-02	1.09E-01	**4.55E+00**	**1.59E+01**	2.30E-02	2.17E-01	**9.10E+00**	**3.18E+01**	3.45E-02	3.26E-01	**1.36E+01**	**4.77E+01**	4.60E-02	4.35E-01	**1.82E+01**	**6.36E+01**
25	C133 /I/22/23	2.78E-03	-	7.64E-01	3.95E-01	1.39E-02	-	**3.82E+00**	**1.98E+00**	2.78E-02	-	**7.64E+00**	**3.95E+00**	4.17E-02	-	**1.15E+01**	**5.93E+00**	5.56E-02	-	**1.53E+01**	**7.90E+00**
26	C134 /I/22/23	2.41E-03	-	-	9.21E-01	1.21E-02	-	-	**4.61E+00**	2.41E-02	-	-	**9.21E+00**	3.62E-02	-	-	**1.38E+01**	4.82E-02	-	-	**1.84E+01**
27	C135 /I/22/23	2.05E-03	-	5.66E-01	5.44E-01	1.02E-02	-	**2.83E+00**	**2.72E+00**	2.05E-02	-	**5.66E+00**	**5.44E+00**	3.07E-02	-	**8.49E+00**	**8.16E+00**	4.10E-02	-	**1.13E+01**	**1.09E+01**
28	C136 /I/22/23	2.20E-03	-	**1.01E+00**	9.29E-01	1.10E-02	-	**5.03E+00**	**4.65E+00**	2.20E-02	-	**1.01E+01**	**9.29E+00**	3.30E-02	-	**1.51E+01**	**1.39E+01**	4.40E-02	-	**2.01E+01**	**1.86E+01**
29	C137 /I/22/23	2.03E-03	1.45E-02	**1.11E+00**	3.74E-01	1.01E-02	7.23E-02	**5.57E+00**	**1.87E+00**	2.03E-02	1.45E-01	**1.11E+01**	**3.74E+00**	3.04E-02	2.17E-01	**1.67E+01**	**5.61E+00**	4.05E-02	2.89E-01	**2.23E+01**	**7.48E+00**
30	C138 /I/22/23	2.29E-03	-	5.75E-01	4.63E-01	1.15E-02	-	**2.88E+00**	**2.31E+00**	2.29E-02	-	**5.75E+00**	**4.63E+00**	3.44E-02	-	**8.63E+00**	**6.94E+00**	4.58E-02	-	**1.15E+01**	**9.26E+00**
31	C169 /I/22/23	2.10E-03	1.96E-02	-	**1.18E+00**	1.05E-02	9.82E-02	-	**5.91E+00**	2.10E-02	1.96E-01	-	**1.18E+01**	3.15E-02	2.95E-01	-	**1.77E+01**	4.20E-02	3.93E-01	-	**2.36E+01**
32	C170 /I/22/23	2.43E-03	-	2.37E-01	4.88E-01	1.22E-02	-	**1.18E+00**	**2.44E+00**	2.43E-02	-	**2.37E+00**	**4.88E+00**	3.65E-02		**3.55E+00**	**7.33E+00**	4.86E-02	-	**4.74E+00**	**9.77E+00**
33	C171 /I/22/23	2.24E-03	-	1.89E-01	6.01E-01	1.12E-02	-	9.43E-01	**3.00E+00**	2.24E-02	-	1.89E+00	**6.01E+00**	3.36E-02		**2.83E+00**	**9.01E+00**	4.47E-02	-	**3.77E+00**	**1.20E+01**
34	C172 /I/22/23	2.45E-03	-	-	4.82E-01	1.22E-02	-	-	**2.41E+00**	2.45E-02	-	-	**4.82E+00**	3.67E-02		-	**7.23E+00**	4.90E-02	-	-	**9.64E+00**
35	C173 /I/22/23	2.39E-03	-	2.31E-01	5.51E-01	1.19E-02	-	**1.16E+00**	**2.75E+00**	2.39E-02	-	**2.31E+00**	**5.51E+00**	3.58E-02		**3.47E+00**	**8.26E+00**	4.78E-02	-	**4.62E+00**	**1.10E+01**
36	C174 /I/22/23	2.73E-03	-	2.38E-01	7.90E-01	1.36E-02	-	**1.19E+00**	**3.95E+00**	2.73E-02	-	**2.38E+00**	**7.90E+00**	4.09E-02		**3.58E+00**	**1.19E+01**	5.45E-02	-	**4.77E+00**	**1.58E+01**

37	C176 /I/22/23	1.79E-03	1.28E-02	2.31E-01	7.18E-01	8.94E-03	6.41E-02	**1.16E+00**	**3.59E+00**	1.79E-02	1.28E-01	**2.31E+00**	**7.18E+00**	2.68E-02	1.92E-01	**3.47E+00**	**1.08E+01**	3.58E-02	2.56E-01	**4.63E+00**	**1.44E+01**
38	C177 /I/22/23	2.18E-03	-	1.63E-01	7.22E-01	1.09E-02	-	8.14E-01	**3.61E+00**	2.18E-02	-	1.63E+00	**7.22E+00**	3.28E-02	-	**2.44E+00**	**1.08E+01**	4.37E-02	-	**3.26E+00**	**1.44E+01**
39	C178 /I/22/23	2.48E-03	1.71E-02	1.85E-01	8.92E-01	1.24E-02	8.55E-02	9.27E-01	**4.46E+00**	2.48E-02	1.71E-01	1.85E+00	**8.92E+00**	3.72E-02	2.57E-01	**2.78E+00**	**1.34E+01**	4.95E-02	3.42E-01	**3.71E+00**	**1.78E+01**
40	C179 /I/22/23	2.07E-03	-	-	6.43E-01	1.04E-02	-	-	**3.21E+00**	2.07E-02	-	-	**6.43E+00**	3.11E-02	-	-	**9.64E+00**	4.14E-02	-	-	**1.29E+01**
41	C180 /I/22/23	1.89E-03	-	2.81E-01	6.15E-01	9.46E-03	-	**1.41E+00**	**3.08E+00**	1.89E-02	-	**2.81E+00**	**6.15E+00**	2.84E-02	-	**4.22E+00**	**9.23E+00**	3.78E-02	-	**5.63E+00**	**1.23E+01**
42	C181 /I/22/23	2.14E-03	9.48E-03	2.42E-01	4.10E-01	1.07E-02	4.74E-02	**1.21E+00**	**2.05E+00**	2.14E-02	9.48E-02	**2.42E+00**	**4.10E+00**	3.22E-02	1.42E-01	**3.63E+00**	**6.15E+00**	4.29E-02	1.90E-01	**4.84E+00**	**8.19E+00**
43	C182 /I/22/23	2.40E-03	1.65E-02	2.09E-01	5.29E-01	1.20E-02	8.24E-02	**1.04E+00**	**2.65E+00**	2.40E-02	1.65E-01	**2.09E+00**	**5.29E+00**	3.60E-02	2.47E-01	**3.13E+00**	**7.94E+00**	4.80E-02	3.29E-01	**4.17E+00**	**1.06E+01**
44	C183 /I/22/23	2.83E-03	1.76E-02	-	6.14E-01	1.41E-02	8.82E-02	-	**3.07E+00**	2.83E-02	1.76E-01	-	**6.14E+00**	4.24E-02	2.64E-01	-	**9.22E+00**	5.65E-02	3.53E-01	-	**1.23E+01**
45	C184 /I/22/23	2.07E-03	2.44E-02	-	3.47E-01	1.04E-02	1.22E-01	-	**1.73E+00**	2.07E-02	2.44E-01	-	**3.47E+00**	3.11E-02	3.66E-01	-	**5.20E+00**	4.14E-02	4.88E-01	-	**6.94E+00**
46	C185 /I/22/23	2.50E-03	9.29E-03	-	2.66E-01	1.25E-02	4.64E-02	-	**1.33E+00**	2.50E-02	9.29E-02	-	**2.66E+00**	3.74E-02	1.39E-01	-	**3.99E+00**	4.99E-02	1.86E-01	-	**5.32E+00**
47	C186 /I/22/23	1.92E-03	-	2.11E-01	3.54E-01	9.59E-03	-	**1.06E+00**	**1.77E+00**	1.92E-02	-	**2.11E+00**	**3.54E+00**	2.88E-02	-	**3.17E+00**	**5.32E+00**	3.84E-02	-	**4.23E+00**	**7.09E+00**
48	C187 /I/22/23	2.97E-03	-	1.85E-01	4.69E-01	1.49E-02	-	9.23E-01	**2.34E+00**	2.97E-02	-	1.85E+00	**4.69E+00**	4.46E-02	-	**2.77E+00**	**7.03E+00**	5.95E-02	-	**3.69E+00**	**9.38E+00**
49	C188 /I/22/23	2.21E-03	3.56E-02	2.45E-01	3.01E-01	1.10E-02	1.78E-01	**1.23E+00**	**1.50E+00**	2.21E-02	3.56E-01	**2.45E+00**	**3.01E+00**	3.31E-02	5.34E-01	**3.68E+00**	**4.51E+00**	4.42E-02	7.11E-01	**4.91E+00**	**6.02E+00**
50	C64 /II/22/23	3.98E-03	-	2.19E-01	7.17E-01	1.99E-02	-	**1.10E+00**	**3.59E+00**	3.98E-02	-	**2.19E+00**	**7.17E+00**	5.97E-02	-	**3.29E+00**	**1.08E+01**	7.96E-02	-	**4.38E+00**	**1.43E+01**
51	C65 /II/22/23	2.81E-03	-	1.00E+00	6.50E-01	1.41E-02	-	**5.00E+00**	**3.25E+00**	2.81E-02	-	**1.00E+01**	**6.50E+00**	4.22E-02	-	**1.50E+01**	**9.74E+00**	5.63E-02	-	**2.00E+01**	**1.30E+01**
52	C66 /II/22/23	3.85E-03	-	-	7.39E-01	1.93E-02	-	-	**3.70E+00**	3.85E-02	-		**7.39E+00**	5.78E-02	-	-	**1.11E+01**	7.71E-02	-	-	**1.48E+01**
53	C67 /II/22/23	2.88E-03	-	2.69E-01	6.35E-01	1.44E-02	-	**1.34E+00**	**3.18E+00**	2.88E-02	-	**2.69E+00**	**6.35E+00**	4.32E-02	-	**4.03E+00**	**9.53E+00**	5.76E-02	-	**5.37E+00**	**1.27E+01**
54	C68 /II/22/23	2.42E-03	-	-	5.43E-01	1.21E-02	-	-	**2.71E+00**	2.42E-02	-	-	**5.43E+00**	3.64E-02	-	-	**8.14E+00**	4.85E-02	-	-	**1.09E+01**
55	C69 /II/22/23	2.50E-03	-	3.00E-01	5.24E-01	1.25E-02	-	**1.50E+00**	**2.62E+00**	2.50E-02	-	**3.00E+00**	**5.24E+00**	3.75E-02	-	**4.50E+00**	**7.86E+00**	5.01E-02	-	**6.00E+00**	**1.05E+01**

56	C70 /II/22/23	2.45E-03	-	-	6.03E-01	1.23E-02	-	-	**3.01E+00**	2.45E-02	-	-	**6.03E+00**	3.68E-02	-	-	**9.04E+00**	4.91E-02	-	-	**1.21E+01**
57	C128 /II/22/23	3.43E-03	**9.33E+01**	-	5.43E-01	1.71E-02	**4.67E+02**	-	**2.72E+00**	3.43E-02	**9.33E+02**	-	**5.43E+00**	5.14E-02	**1.40E+03**	-	**8.15E+00**	6.86E-02	**1.87E+03**	-	**1.09E+01**
58	C129 /II/22/23	2.89E-03	**4.83E+01**	-	5.97E-01	1.44E-02	**2.42E+02**	-	**2.99E+00**	2.89E-02	**4.83E+02**	-	**5.97E+00**	4.33E-02	**7.25E+02**	-	**8.96E+00**	5.78E-02	**9.67E+02**	-	**1.19E+01**
59	C130 /II/22/23	2.39E-03	**2.38E+01**	-	5.54E-01	1.20E-02	**1.19E+02**	-	**2.77E+00**	2.39E-02	**2.38E+02**	-	**5.54E+00**	3.59E-02	**3.57E+02**	-	**8.31E+00**	4.79E-02	**4.76E+02**	-	**1.11E+01**
60	C131 /II/22/23	2.57E-03	**6.78E+00**	-	8.24E-01	1.28E-02	**3.39E+01**	-	**4.12E+00**	2.57E-02	**6.78E+01**	-	**8.24E+00**	3.85E-02	**1.02E+02**	-	**1.24E+01**	5.14E-02	**1.36E+02**	-	**1.65E+01**
61	C133 /II/22/23	2.48E-03	**3.90E+00**	-	5.55E-01	1.24E-02	**1.95E+01**	-	**2.77E+00**	2.48E-02	**3.90E+01**	-	**5.55E+00**	3.71E-02	**5.85E+01**	-	**8.32E+00**	4.95E-02	**7.81E+01**	-	**1.11E+01**
62	C134 /II/22/23	3.20E-03	**7.89E+00**	-	6.25E-01	1.60E-02	**3.94E+01**	-	**3.13E+00**	3.20E-02	**7.89E+01**	-	**6.25E+00**	4.80E-02	**1.18E+02**	-	**9.38E+00**	6.39E-02	**1.58E+02**	-	**1.25E+01**
63	C135 /II/22/23	2.46E-03	**3.37E+00**	-	4.86E-01	1.23E-02	**1.68E+01**	-	**2.43E+00**	2.46E-02	**3.37E+01**	-	**4.86E+00**	3.69E-02	**5.05E+01**	-	**7.29E+00**	4.92E-02	**6.73E+01**	-	**9.71E+00**
64	C136 /II/22/23	2.56E-03	**2.09E+00**	5.71E-01	8.71E-01	1.28E-02	**1.05E+01**	**2.86E+00**	**4.36E+00**	2.56E-02	**2.09E+01**	**5.71E+00**	**8.71E+00**	3.84E-02	**3.14E+01**	**8.57E+00**	**1.31E+01**	5.12E-02	**4.19E+01**	**1.14E+01**	**1.74E+01**
65	C137 /II/22/23	2.51E-03	7.27E-01	4.39E-01	4.13E-01	1.26E-02	**3.63E+00**	**2.20E+00**	**2.06E+00**	2.51E-02	**7.27E+00**	**4.39E+00**	**4.13E+00**	3.77E-02	**1.09E+01**	**6.59E+00**	**6.19E+00**	5.03E-02	**1.45E+01**	**8.78E+00**	**8.25E+00**
66	C138 /II/22/23	2.73E-03	8.84E-01	6.67E-01	8.71E-01	1.36E-02	**4.42E+00**	**3.34E+00**	**4.35E+00**	2.73E-02	**8.84E+00**	**6.67E+00**	**8.71E+00**	4.09E-02	**1.33E+01**	**1.00E+01**	**1.31E+01**	5.46E-02	**1.77E+01**	**1.33E+01**	**1.74E+01**
67	C139 /II/22/23	3.03E-03	5.42E-01	-	5.11E-01	1.52E-02	**2.71E+00**	-	**2.56E+00**	3.03E-02	**5.42E+00**	-	**5.11E+00**	4.55E-02	**8.13E+00**	-	**7.67E+00**	6.07E-02	**1.08E+01**	-	**1.02E+01**
68	C140 /II/22/23	2.20E-03	4.21E-01	-	4.53E-01	1.10E-02	**2.10E+00**	-	**2.27E+00**	2.20E-02	**4.21E+00**	-	**4.53E+00**	3.31E-02	**6.31E+00**	-	**6.80E+00**	4.41E-02	**8.41E+00**	-	**9.06E+00**
69	C141 /II/22/23	2.34E-03	4.56E-01	-	4.44E-01	1.17E-02	**2.28E+00**	-	**2.22E+00**	2.34E-02	**4.56E+00**	-	**4.44E+00**	3.51E-02	**6.84E+00**	-	**6.65E+00**	4.68E-02	**9.12E+00**	-	**8.87E+00**
70	C142 /II/22/23	2.37E-03	**1.07E+00**	-	5.53E-01	1.18E-02	**5.36E+00**	-	**2.77E+00**	2.37E-02	**1.07E+01**	-	**5.53E+00**	3.55E-02	**1.61E+01**	-	**8.30E+00**	4.73E-02	**2.15E+01**	-	**1.11E+01**
71	C143 /II/22/23	3.10E-03	-	-	6.94E-01	1.55E-02	-	-	**3.47E+00**	3.10E-02	-	-	**6.94E+00**	4.65E-02	-	-	**1.04E+01**	6.20E-02	-	-	**1.39E+01**
72	C144 /II/22/23	2.52E-03	**1.37E+00**	-	5.50E-01	1.26E-02	**6.85E+00**	-	**2.75E+00**	2.52E-02	**1.37E+01**	-	**5.50E+00**	3.78E-02	**2.06E+01**	-	**8.25E+00**	5.04E-02	**2.74E+01**	-	**1.10E+01**
73	C145 /II/22/23	3.02E-03	3.81E-01	-	9.06E-01	1.51E-02	**1.91E+00**	-	**4.53E+00**	3.02E-02	**3.81E+00**	-	**9.06E+00**	4.52E-02	**5.72E+00**	-	**1.36E+01**	6.03E-02	**7.62E+00**	-	**1.81E+01**
74	C146 /II/22/23	3.82E-03	8.27E-01	1.59E-01	7.79E-01	1.91E-02	**4.13E+00**	7.95E-01	**3.89E+00**	3.82E-02	**8.27E+00**	1.59E+00	**7.79E+00**	5.72E-02	**1.24E+01**	**2.39E+00**	**1.17E+01**	7.63E-02	**1.65E+01**	**3.18E+00**	**1.56E+01**

75	C147 /II/22/23	3.09E-03	2.71E-01	-	5.24E-01	1.55E-02	**1.35E+00**	-	**2.62E+00**	3.09E-02	**2.71E+00**	-	**5.24E+00**	4.64E-02	**4.06E+00**	-	**7.86E+00**	6.18E-02	**5.42E+00**	-	**1.05E+01**
76	C148 /II/22/23	3.23E-03	2.32E-01	-	6.44E-01	1.62E-02	**1.16E+00**	-	**3.22E+00**	3.23E-02	**2.32E+00**	-	**6.44E+00**	4.85E-02	**3.48E+00**	-	**9.66E+00**	6.46E-02	**4.65E+00**	-	**1.29E+01**
77	C149 /II/22/23	3.09E-03	-	-	7.49E-01	1.54E-02	-	-	**3.75E+00**	3.09E-02	-	-	**7.49E+00**	4.63E-02	-	-	**1.12E+01**	6.17E-02	-	-	**1.50E+01**
78	C15 /II/22/23	2.55E-03	5.85E-01	-	7.56E-01	1.28E-02	**2.93E+00**	-	**3.78E+00**	2.55E-02	**5.85E+00**	-	**7.56E+00**	3.83E-02	**8.78E+00**	-	**1.13E+01**	5.10E-02	**1.17E+01**	-	**1.51E+01**
79	C86 /II/21/22	3.00E-03	2.09E-02	-	7.94E-01	1.50E-02	1.05E-01	-	**3.97E+00**	3.00E-02	2.09E-01	-	**7.94E+00**	4.50E-02	3.14E-01	-	**1.19E+01**	6.00E-02	4.18E-01	-	**1.59E+01**
80	C87 /II/21/22	2.44E-03	-	-	5.64E-01	1.22E-02	-	-	**2.82E+00**	2.44E-02	-	-	**5.64E+00**	3.67E-02	-	-	**8.47E+00**	4.89E-02	-	-	**1.13E+01**
81	C88 /II/21/22	2.57E-03	1.30E-02	-	7.80E-01	1.29E-02	6.52E-02	-	**3.90E+00**	2.57E-02	1.30E-01	-	**7.80E+00**	3.86E-02	1.96E-01	-	**1.17E+01**	5.14E-02	2.61E-01	-	**1.56E+01**
82	C89 /II/21/22	2.52E-03	1.43E-02	1.67E-01	4.08E-01	1.26E-02	7.14E-02	8.35E-01	**2.04E+00**	2.52E-02	1.43E-01	1.67E+00	**4.08E+00**	3.78E-02	2.14E-01	**2.51E+00**	**6.12E+00**	5.04E-02	2.86E-01	**3.34E+00**	**8.16E+00**
83	C90 /II/21/22	2.50E-03	1.42E-02	2.47E-01	8.18E-01	1.25E-02	7.11E-02	**1.23E+00**	**4.09E+00**	2.50E-02	1.42E-01	**2.47E+00**	**8.18E+00**	3.74E-02	2.13E-01	**3.70E+00**	**1.23E+01**	4.99E-02	2.84E-01	**4.93E+00**	**1.64E+01**
84	C91 /II/21/22	2.57E-03	-	-	7.50E-01	1.29E-02	-	-	**3.75E+00**	2.57E-02	-	-	**7.50E+00**	3.86E-02	-		**1.12E+01**	5.15E-02	-	-	**1.50E+01**
85	C92 /II/21/22	2.25E-03	1.67E-02	-	7.65E-01	1.12E-02	8.36E-02	-	**3.83E+00**	2.25E-02	1.67E-01	-	**7.65E+00**	3.37E-02	2.51E-01		**1.15E+01**	4.49E-02	3.35E-01	-	**1.53E+01**
86	C93 /II/21/22	2.18E-03	1.05E-02	-	4.14E-01	1.09E-02	5.27E-02	-	**2.07E+00**	2.18E-02	1.05E-01	-	**4.14E+00**	3.26E-02	1.58E-01		**6.21E+00**	4.35E-02	2.11E-01	-	**8.28E+00**
87	C94 /II/21/22	2.00E-03	1.23E-02	1.69E-01	3.88E-01	1.00E-02	6.13E-02	8.43E-01	**1.94E+00**	2.00E-02	1.23E-01	1.69E+00	**3.88E+00**	3.00E-02	1.84E-01	**2.53E+00**	**5.82E+00**	4.00E-02	2.45E-01	**3.37E+00**	**7.76E+00**
88	C95 /II/21/22	2.19E-03	1.10E-02	-	3.31E-01	1.10E-02	5.52E-02	-	**1.65E+00**	2.19E-02	1.10E-01	-	**3.31E+00**	3.29E-02	1.66E-01		**4.96E+00**	4.39E-02	2.21E-01	-	**6.62E+00**
89	C96 /II/21/22	2.34E-03	-	-	4.96E-01	1.17E-02	-	-	**2.48E+00**	2.34E-02	-	-	**4.96E+00**	3.51E-02			**7.44E+00**	4.68E-02	-	-	**9.92E+00**
90	C97 /II/21/22	2.48E-03	-	1.77E-01	5.36E-01	1.24E-02	-	8.87E-01	**2.68E+00**	2.48E-02	-	1.77E+00	**5.36E+00**	3.73E-02		**2.66E+00**	**8.04E+00**	4.97E-02	-	**3.55E+00**	**1.07E+01**
91	C98 /II/21/22	4.79E-03	-	-	5.12E-01	2.39E-02	-	-	**2.56E+00**	4.79E-02	-	-	**5.12E+00**	7.18E-02			**7.69E+00**	9.58E-02	-	-	**1.02E+01**
92	C99 /II/21/22	3.63E-03	1.44E-02	1.66E-01	8.35E-01	1.81E-02	7.20E-02	8.29E-01	**4.17E+00**	3.63E-02	1.44E-01	1.66E+00	**8.35E+00**	5.44E-02	2.16E-01	**2.49E+00**	**1.25E+01**	7.25E-02	2.88E-01	**3.32E+00**	**1.67E+01**
93	C101 /II/21/22	2.88E-03	1.59E-02	1.86E-01	8.47E-01	1.44E-02	7.93E-02	9.30E-01	**4.24E+00**	2.88E-02	1.59E-01	1.86E+00	**8.47E+00**	4.32E-02	2.38E-01	**2.79E+00**	**1.27E+01**	5.77E-02	3.17E-01	**3.72E+00**	**1.69E+01**

94	C16 /II/21/22	2.11E-03	-	2.13E-01	6.21E-01	1.05E-02	-	**1.06E+00**	**3.11E+00**	2.11E-02	-	**2.13E+00**	**6.21E+00**	3.16E-02	-	**3.19E+00**	**9.32E+00**	4.21E-02	-	**4.26E+00**	**1.24E+01**
95	C17 /II/21/22	2.04E-03	2.22E-02	-	**1.09E+00**	1.02E-02	1.11E-01	-	**5.43E+00**	2.04E-02	2.22E-01	-	**1.09E+01**	3.05E-02	3.33E-01	-	**1.63E+01**	4.07E-02	4.45E-01	-	**2.17E+01**
96	C18 /II/21/22	2.03E-03	8.93E-03	**1.47E+00**	8.09E-01	1.01E-02	4.46E-02	**7.35E+00**	**4.04E+00**	2.03E-02	8.93E-02	**1.47E+01**	**8.09E+00**	3.04E-02	1.34E-01	**2.21E+01**	**1.21E+01**	4.05E-02	1.79E-01	**2.94E+01**	**1.62E+01**
97	C21 /II/21/22	1.26E-03	-	2.78E-01	**1.03E+00**	6.30E-03	-	**1.39E+00**	**5.15E+00**	1.26E-02	-	**2.78E+00**	**1.03E+01**	1.89E-02	-	**4.18E+00**	**1.54E+01**	2.52E-02	-	**5.57E+00**	**2.06E+01**
98	C39 /II/21/22	2.52E-03	-	3.03E-01	7.21E-01	1.26E-02	-	**1.52E+00**	**3.60E+00**	2.52E-02	-	**3.03E+00**	**7.21E+00**	3.77E-02	-	**4.55E+00**	**1.08E+01**	5.03E-02	-	**6.06E+00**	**1.44E+01**
99	C40 /II/21/22	2.21E-03	-	2.73E-01	9.09E-01	1.11E-02	-	**1.37E+00**	**4.54E+00**	2.21E-02	-	**2.73E+00**	**9.09E+00**	3.32E-02	-	**4.10E+00**	**1.36E+01**	4.43E-02	-	**5.47E+00**	**1.82E+01**
100	C41 /II/21/22	2.98E-03	1.89E-02	2.44E-01	7.30E-01	1.49E-02	9.43E-02	**1.22E+00**	**3.65E+00**	2.98E-02	1.89E-01	**2.44E+00**	**7.30E+00**	4.48E-02	2.83E-01	**3.66E+00**	**1.10E+01**	5.97E-02	3.77E-01	**4.88E+00**	**1.46E+01**
101	C43 /II/21/22	4.94E-03	9.55E-03	6.11E-01	9.35E-01	2.47E-02	4.78E-02	**3.06E+00**	**4.68E+00**	4.94E-02	9.55E-02	**6.11E+00**	**9.35E+00**	7.41E-02	1.43E-01	**9.17E+00**	**1.40E+01**	9.88E-02	1.91E-01	**1.22E+01**	**1.87E+01**
102	C44 /II/21/22	1.98E-02	-	2.37E-01	9.95E-01	9.92E-02	-	**1.19E+00**	**4.98E+00**	1.98E-01	-	**2.37E+00**	**9.95E+00**	2.98E-01		**3.56E+00**	**1.49E+01**	3.97E-01	-	**4.74E+00**	**1.99E+01**
103	C46 /II/21/22	3.14E-03	-	4.02E-01	8.68E-01	1.57E-02	-	**2.01E+00**	**4.34E+00**	3.14E-02	-	**4.02E+00**	**8.68E+00**	4.71E-02		**6.03E+00**	**1.30E+01**	6.28E-02	-	**8.03E+00**	**1.74E+01**
104	C314 /II/17/18	-	**7.51E+00**	-	**7.35E+00**	-	**3.75E+01**	-	**3.67E+01**	-	**7.51E+01**	-	**7.35E+01**	-	**1.13E+02**	-	**1.10E+02**	-	**1.50E+02**	-	**1.47E+02**
105	C315 /II/17/18	-	8.20E-01	-	**4.29E+00**	-	**4.10E+00**	-	**2.15E+01**	-	**8.20E+00**	-	**4.29E+01**	-	**1.23E+01**	-	**6.44E+01**	-	**1.64E+01**	-	**8.59E+01**
106	C319 /II/17/18	-	4.62E-01	-	4.80E-01	-	**2.31E+00**	-	**2.40E+00**	-	**4.62E+00**	-	**4.80E+00**	-	**6.93E+00**	-	**7.20E+00**	-	**9.23E+00**	-	**9.60E+00**
107	C320 /II/17/18	-	2.63E-01	-	**2.45E+00**	-	**1.31E+00**	-	**1.23E+01**	-	**2.63E+00**	-	**2.45E+01**	-	**3.94E+00**	-	**3.68E+01**	-	**5.26E+00**	-	**4.90E+01**
108	C321 /II/17/18	-	9.00E-01	-	**2.46E+00**	-	**4.50E+00**	-	**1.23E+01**	-	**9.00E+00**	-	**2.46E+01**	-	**1.35E+01**	-	**3.69E+01**	-	**1.80E+01**	-	**4.92E+01**
109	C322 /II/17/18	-	9.55E-01	-	9.26E-01	-	**4.77E+00**	-	**4.63E+00**	-	**9.55E+00**	-	**9.26E+00**	-	**1.43E+01**	-	**1.39E+01**	-	**1.91E+01**	-	**1.85E+01**
110	C323 /II/17/18	8.56E-03	-	4.93E-01	2.49E-01	4.28E-02	-	**2.47E+00**	**1.24E+00**	8.56E-02	-	**4.93E+00**	**2.49E+00**	1.28E-01	-	**7.40E+00**	**3.73E+00**	1.71E-01	-	**9.87E+00**	**4.97E+00**
111	C324 /II/17/18	-	7.82E-01	-	**2.56E+00**	-	**3.91E+00**	-	**1.28E+01**	-	**7.82E+00**	-	**2.56E+01**	-	**1.17E+01**	-	**3.84E+01**	-	**1.56E+01**	-	**5.13E+01**

112	C35 /I/23/24	1.88E-03	1.06E-01	-	8.40E-01	9.41E-03	5.29E-01	-	**4.20E+00**	1.88E-02	1.06E+00	-	**8.40E+00**	2.82E-02	**1.59E+00**	-	**1.26E+01**	3.76E-02	**2.12E+00**	-	**1.68E+01**
113	C168 /I/22/23	2.11E-03	-	-	3.98E-01	1.06E-02	-	-	**1.99E+00**	2.11E-02		-	**3.98E+00**	3.17E-02	-	-	**5.97E+00**	4.22E-02	-	-	**7.96E+00**
114	C175 /I/22/23	2.09E-03	1.60E-02	-	5.75E-01	1.05E-02	7.98E-02	-	**2.87E+00**	2.09E-02	1.60E-01	-	**5.75E+00**	3.14E-02	2.39E-01	-	**8.62E+00**	4.19E-02	3.19E-01	-	**1.15E+01**
115	C100 /II/21/22	1.34E-03	1.05E-02	-	**1.28E+00**	6.72E-03	5.27E-02	-	**6.42E+00**	1.34E-02	1.05E-01	-	**1.28E+01**	2.02E-02	1.58E-01	-	**1.93E+01**	2.69E-02	2.11E-01	-	**2.57E+01**
116	C20 /II/21/22	1.93E-03	1.23E-02	3.93E-01	9.31E-01	9.64E-03	6.13E-02	**1.96E+00**	**4.65E+00**	1.93E-02	1.23E-01	**3.93E+00**	**9.31E+00**	2.89E-02	1.84E-01	**5.89E+00**	**1.40E+01**	3.86E-02	2.45E-01	**7.85E+00**	**1.86E+01**
117	C316 /II/17/18	-	4.83E-01	-	8.23E-01	-	**2.42E+00**	-	**4.11E+00**	-	**4.83E+00**	-	**8.23E+00**	-	**7.25E+00**	-	**1.23E+01**	-	**9.67E+00**	-	**1.65E+01**
118	C317 /II/17/18	-	6.42E-01	-	5.31E-01	-	**3.21E+00**	-	**2.66E+00**	-	**6.42E+00**	-	**5.31E+00**	-	**9.63E+00**	-	**7.97E+00**	-	**1.28E+01**	-	**1.06E+01**
119	C326 /II/17/18	7.20E-04	4.31E-01	5.22E-01	**3.47E+00**	3.60E-03	**2.15E+00**	**2.61E+00**	**1.74E+01**	7.20E-03	**4.31E+00**	**5.22E+00**	**3.47E+01**	1.08E-02	**6.46E+00**	**7.83E+00**	**5.21E+01**	1.44E-02	**8.62E+00**	**1.04E+01**	**6.94E+01**

Table S5. Average Daily Dose (ADD) [mg/kg] for ingestion exposure to copper, lead, nickel, and cadmium contained in the cigarettes tested.

No.	Sample code	1 cigarette per day				5 cigarettes per day				10 cigarettes per day				15 cigarettes per day				20 cigarettes per day			
		Cu	Pb	Ni	Cd	Cu	Pb	Ni	Cd	Cu	Pb	Ni	Cd	Cu	Pb	Ni	Cd	Cu	Pb	Ni	Cd
1	C20 /I/23/24	7.58E-06	1.64E-06	-	6.97E-07	3.79E-05	8.19E-06	-	3.49E-06	7.58E-05	1.64E-05	-	6.97E-06	1.14E-04	2.46E-05	-	1.05E-05	1.52E-04	3.27E-05	-	1.39E-05
2	C21 /I/23/24	7.61E-06	5.50E-07	-	5.30E-07	3.81E-05	2.75E-06	-	2.65E-06	7.61E-05	5.50E-06	-	5.30E-06	1.14E-04	8.25E-06	-	7.95E-06	1.52E-04	1.10E-05	-	1.06E-05
3	C22 /I/23/24	6.17E-06	1.30E-06	-	6.60E-07	3.08E-05	6.52E-06	-	3.30E-06	6.17E-05	1.30E-05	-	6.60E-06	9.25E-05	1.96E-05	-	9.90E-06	1.23E-04	2.61E-05	-	1.32E-05
4	C23 /I/23/24	6.78E-06	1.19E-06	-	7.76E-07	3.39E-05	5.96E-06	-	3.88E-06	6.78E-05	1.19E-05	-	7.76E-06	1.02E-04	1.79E-05	-	1.16E-05	1.36E-04	2.38E-05	-	1.55E-05
5	C24 /I/23/24	6.63E-06	1.39E-07	-	6.22E-07	3.31E-05	6.93E-07	-	3.11E-06	6.63E-05	1.39E-06	-	6.22E-06	9.94E-05	2.08E-06	-	9.33E-06	1.33E-04	2.77E-06	-	1.24E-05
6	C25 /I/23/24	6.07E-06	1.09E-06	-	5.81E-07	3.03E-05	5.44E-06	-	2.90E-06	6.07E-05	1.09E-05	-	5.81E-06	9.10E-05	1.63E-05	-	8.71E-06	1.21E-04	2.18E-05	-	1.16E-05
7	C26 /I/23/24	6.59E-06	1.11E-06	-	1.22E-06	3.30E-05	5.53E-06	-	6.10E-06	6.59E-05	1.11E-05	-	1.22E-05	9.89E-05	1.66E-05	-	1.83E-05	1.32E-04	2.21E-05	-	2.44E-05
8	C27 /I/23/24	8.46E-06	6.45E-07	-	9.33E-07	4.23E-05	3.23E-06	-	4.66E-06	8.46E-05	6.45E-06	-	9.33E-06	1.27E-04	9.68E-06	-	1.40E-05	1.69E-04	1.29E-05	-	1.87E-05
9	C28 /I/23/24	6.86E-06	8.96E-07	-	6.26E-07	3.43E-05	4.48E-06	-	3.13E-06	6.86E-05	8.96E-06	-	6.26E-06	1.03E-04	1.34E-05	-	9.39E-06	1.37E-04	1.79E-05	-	1.25E-05
10	C29 /I/23/24	8.31E-06	5.36E-07	-	9.17E-07	4.16E-05	2.68E-06	-	4.58E-06	8.31E-05	5.36E-06	-	9.17E-06	1.25E-04	8.04E-06	-	1.37E-05	1.66E-04	1.07E-05	-	1.83E-05
11	C30 /I/23/24	7.94E-06	-	-	7.86E-07	3.97E-05	-	-	3.93E-06	7.94E-05	-	-	7.86E-06	1.19E-04	-	-	1.18E-05	1.59E-04	-	-	1.57E-05
12	C31 /I/23/24	6.40E-06	1.18E-06	-	5.97E-07	3.20E-05	5.92E-06	-	2.98E-06	6.40E-05	1.18E-05	-	5.97E-06	9.60E-05	1.78E-05	-	8.95E-06	1.28E-04	2.37E-05	-	1.19E-05
13	C32 /I/23/24	8.09E-06	1.19E-06	-	8.71E-07	4.05E-05	5.96E-06	-	4.36E-06	8.09E-05	1.19E-05	-	8.71E-06	1.21E-04	1.79E-05	-	1.31E-05	1.62E-04	2.38E-05	-	1.74E-05
14	C33 /I/23/24	5.65E-06	9.64E-07	-	6.73E-07	2.82E-05	4.82E-06	-	3.37E-06	5.65E-05	9.64E-06	-	6.73E-06	8.47E-05	1.45E-05	-	1.01E-05	1.13E-04	1.93E-05	-	1.35E-05
15	C34 /I/23/24	8.08E-06	6.73E-07	-	7.60E-07	4.04E-05	3.36E-06	-	3.80E-06	8.08E-05	6.73E-06	-	7.60E-06	1.21E-04	1.01E-05	-	1.14E-05	1.62E-04	1.35E-05	-	1.52E-05
16	C36 /I/23/24	9.20E-06	1.21E-06	-	8.17E-07	4.60E-05	6.05E-06	-	4.09E-06	9.20E-05	1.21E-05	-	8.17E-06	1.38E-04	1.81E-05	-	1.23E-05	1.84E-04	2.42E-05	-	1.63E-05
17	C37 /I/23/24	7.99E-06	2.37E-06	-	9.26E-07	3.99E-05	1.18E-05	-	4.63E-06	7.99E-05	2.37E-05	-	9.26E-06	1.20E-04	3.55E-05	-	1.39E-05	1.60E-04	4.74E-05	-	1.85E-05

18	C38 /I/23/24	6.32E-06	1.51E-06	-	7.96E-07	3.16E-05	7.55E-06	-	3.98E-06	6.32E-05	1.51E-05	-	7.96E-06	9.48E-05	2.26E-05	-	1.19E-05	1.26E-04	3.02E-05	-	1.59E-05
19	C39 /I/23/24	8.50E-06	8.52E-07	-	5.47E-07	4.25E-05	4.26E-06	-	2.73E-06	8.50E-05	8.52E-06	-	5.47E-06	1.27E-04	1.28E-05	-	8.20E-06	1.70E-04	1.70E-05	-	1.09E-05
20	C128 /I/22/23	8.58E-06	1.66E-07	1.99E-06	6.15E-07	4.29E-05	8.31E-07	9.94E-06	3.08E-06	8.58E-05	1.66E-06	1.99E-05	6.15E-06	1.29E-04	2.49E-06	2.98E-05	9.23E-06	1.72E-04	3.32E-06	3.98E-05	1.23E-05
21	C129 /I/22/23	9.21E-06	-	1.96E-06	3.88E-07	4.61E-05	-	9.82E-06	1.94E-06	9.21E-05	-	1.96E-05	3.88E-06	1.38E-04	-	2.95E-05	5.82E-06	1.84E-04	-	3.93E-05	7.75E-06
22	C130 /I/22/23	1.14E-05	2.91E-07	1.94E-06	4.24E-07	5.68E-05	1.46E-06	9.68E-06	2.12E-06	1.14E-04	2.91E-06	1.94E-05	4.24E-06	1.70E-04	4.37E-06	2.91E-05	6.36E-06	2.27E-04	5.82E-06	3.87E-05	8.47E-06
23	C131 /I/22/23	7.06E-06	1.33E-07	3.87E-06	3.22E-07	3.53E-05	6.67E-07	1.94E-05	1.61E-06	7.06E-05	1.33E-06	3.87E-05	3.22E-06	1.06E-04	2.00E-06	5.81E-05	4.83E-06	1.41E-04	2.67E-06	7.74E-05	6.43E-06
24	C132 /I/22/23	9.05E-06	3.21E-07	8.05E-06	3.13E-06	4.53E-05	1.60E-06	4.02E-05	1.56E-05	9.05E-05	3.21E-06	8.05E-05	3.13E-05	1.36E-04	4.81E-06	1.21E-04	4.69E-05	1.81E-04	6.41E-06	1.61E-04	6.25E-05
25	C133 /I/22/23	1.09E-05	-	6.76E-06	3.88E-07	5.47E-05	-	3.38E-05	1.94E-06	1.09E-04	-	6.76E-05	3.88E-06	1.64E-04	-	1.01E-04	5.83E-06	2.19E-04	-	1.35E-04	7.77E-06
26	C134 /I/22/23	9.48E-06	-	-	9.06E-07	4.74E-05	-	-	4.53E-06	9.48E-05	-	-	9.06E-06	1.42E-04	-	-	1.36E-05	1.90E-04	-	-	1.81E-05
27	C135 /I/22/23	8.06E-06	-	5.01E-06	5.35E-07	4.03E-05	-	2.50E-05	2.67E-06	8.06E-05	-	5.01E-05	5.35E-06	1.21E-04	-	7.51E-05	8.02E-06	1.61E-04	-	1.00E-04	1.07E-05
28	C136 /I/22/23	8.66E-06	-	8.90E-06	9.14E-07	4.33E-05	-	4.45E-05	4.57E-06	8.66E-05	-	8.90E-05	9.14E-06	1.30E-04	-	1.33E-04	1.37E-05	1.73E-04	-	1.78E-04	1.83E-05
29	C137 /I/22/23	7.97E-06	2.13E-07	9.87E-06	3.68E-07	3.98E-05	1.07E-06	4.93E-05	1.84E-06	7.97E-05	2.13E-06	9.87E-05	3.68E-06	1.19E-04	3.20E-06	1.48E-04	5.52E-06	1.59E-04	4.27E-06	1.97E-04	7.36E-06
30	C138 /I/22/23	9.01E-06	-	5.09E-06	4.55E-07	4.51E-05	-	2.55E-05	2.28E-06	9.01E-05	-	5.09E-05	4.55E-06	1.35E-04	-	7.64E-05	6.83E-06	1.80E-04	-	1.02E-04	9.11E-06
31	C169 /I/22/23	8.26E-06	2.90E-07	-	1.16E-06	4.13E-05	1.45E-06	-	5.81E-06	8.26E-05	2.90E-06	-	1.16E-05	1.24E-04	4.35E-06	-	1.74E-05	1.65E-04	5.80E-06	-	2.32E-05
32	C170 /I/22/23	9.56E-06	-	2.10E-06	4.80E-07	4.78E-05	-	1.05E-05	2.40E-06	9.56E-05	-	2.10E-05	4.80E-06	1.43E-04	-	3.15E-05	7.20E-06	1.91E-04	-	4.19E-05	9.61E-06
33	C171 /I/22/23	8.80E-06	-	1.67E-06	5.91E-07	4.40E-05	-	8.35E-06	2.95E-06	8.80E-05	-	1.67E-05	5.91E-06	1.32E-04	-	2.50E-05	8.86E-06	1.76E-04	-	3.34E-05	1.18E-05
34	C172 /I/22/23	9.63E-06	-	-	4.74E-07	4.81E-05	-	-	2.37E-06	9.63E-05	-	-	4.74E-06	1.44E-04	-	-	7.11E-06	1.93E-04	-	-	9.48E-06
35	C173 /I/22/23	9.40E-06	-	2.05E-06	5.42E-07	4.70E-05	-	1.02E-05	2.71E-06	9.40E-05	-	2.05E-05	5.42E-06	1.41E-04	-	3.07E-05	8.13E-06	1.88E-04	-	4.09E-05	1.08E-05
36	C174 /I/22/23	1.07E-05	-	2.11E-06	7.77E-07	5.36E-05	-	1.06E-05	3.89E-06	1.07E-04	-	2.11E-05	7.77E-06	1.61E-04	-	3.17E-05	1.17E-05	2.14E-04	-	4.22E-05	1.55E-05

37	C176 /I/22/23	7.04E-06	1.89E-07	2.05E-06	7.06E-07	3.52E-05	9.46E-07	1.02E-05	3.53E-06	7.04E-05	1.89E-06	2.05E-05	7.06E-06	1.06E-04	2.84E-06	3.07E-05	1.06E-05	1.41E-04	3.78E-06	4.10E-05	1.41E-05
38	C177 /I/22/23	8.59E-06	-	1.44E-06	7.10E-07	4.30E-05	-	7.21E-06	3.55E-06	8.59E-05	-	1.44E-05	7.10E-06	1.29E-04	-	2.16E-05	1.07E-05	1.72E-04	-	2.88E-05	1.42E-05
39	C178 /I/22/23	9.74E-06	2.52E-07	1.64E-06	8.77E-07	4.87E-05	1.26E-06	8.20E-06	4.39E-06	9.74E-05	2.52E-06	1.64E-05	8.77E-06	1.46E-04	3.78E-06	2.46E-05	1.32E-05	1.95E-04	5.05E-06	3.28E-05	1.75E-05
40	C179 /I/22/23	8.15E-06	-	-	6.32E-07	4.07E-05	-	-	3.16E-06	8.15E-05	-	-	6.32E-06	1.22E-04	-	-	9.48E-06	1.63E-04	-	-	1.26E-05
41	C180 /I/22/23	7.44E-06	-	2.49E-06	6.05E-07	3.72E-05	-	1.25E-05	3.02E-06	7.44E-05	-	2.49E-05	6.05E-06	1.12E-04	-	3.74E-05	9.07E-06	1.49E-04	-	4.98E-05	1.21E-05
42	C181 /I/22/23	8.43E-06	1.40E-07	2.14E-06	4.03E-07	4.22E-05	6.99E-07	1.07E-05	2.01E-06	8.43E-05	1.40E-06	2.14E-05	4.03E-06	1.26E-04	2.10E-06	3.21E-05	6.04E-06	1.69E-04	2.80E-06	4.28E-05	8.06E-06
43	C182 /I/22/23	9.44E-06	2.43E-07	1.85E-06	5.21E-07	4.72E-05	1.21E-06	9.23E-06	2.60E-06	9.44E-05	2.43E-06	1.85E-05	5.21E-06	1.42E-04	3.64E-06	2.77E-05	7.81E-06	1.89E-04	4.86E-06	3.69E-05	1.04E-05
44	C183 /I/22/23	1.11E-05	2.60E-07	-	6.04E-07	5.56E-05	1.30E-06	-	3.02E-06	1.11E-04	2.60E-06	-	6.04E-06	1.67E-04	3.90E-06	-	9.06E-06	2.22E-04	5.20E-06	-	1.21E-05
45	C184 /I/22/23	8.15E-06	3.60E-07	-	3.41E-07	4.08E-05	1.80E-06	-	1.71E-06	8.15E-05	3.60E-06	-	3.41E-06	1.22E-04	5.40E-06	-	5.12E-06	1.63E-04	7.20E-06	-	6.82E-06
46	C185 /I/22/23	9.82E-06	1.37E-07	-	2.61E-07	4.91E-05	6.85E-07	-	1.31E-06	9.82E-05	1.37E-06	-	2.61E-06	1.47E-04	2.05E-06	-	3.92E-06	1.96E-04	2.74E-06	-	5.23E-06
47	C186 /I/22/23	7.55E-06	-	1.87E-06	3.48E-07	3.77E-05	-	9.35E-06	1.74E-06	7.55E-05	-	1.87E-05	3.48E-06	1.13E-04	-	2.81E-05	5.23E-06	1.51E-04	-	3.74E-05	6.97E-06
48	C187 /I/22/23	1.17E-05	-	1.63E-06	4.61E-07	5.85E-05	-	8.17E-06	2.31E-06	1.17E-04	-	1.63E-05	4.61E-06	1.75E-04	-	2.45E-05	6.92E-06	2.34E-04	-	3.27E-05	9.22E-06
49	C188 /I/22/23	8.69E-06	5.25E-07	2.17E-06	2.96E-07	4.34E-05	2.62E-06	1.09E-05	1.48E-06	8.69E-05	5.25E-06	2.17E-05	2.96E-06	1.30E-04	7.87E-06	3.26E-05	4.44E-06	1.74E-04	1.05E-05	4.35E-05	5.92E-06
50	C64 /II/22/23	1.57E-05	-	1.94E-06	7.05E-07	7.83E-05	-	9.70E-06	3.53E-06	1.57E-04	-	1.94E-05	7.05E-06	2.35E-04	-	2.91E-05	1.06E-05	3.13E-04	-	3.88E-05	1.41E-05
51	C65 /II/22/23	1.11E-05	-	8.85E-06	6.39E-07	5.53E-05	-	4.42E-05	3.19E-06	1.11E-04	-	8.85E-05	6.39E-06	1.66E-04	-	1.33E-04	9.58E-06	2.21E-04	-	1.77E-04	1.28E-05
52	C66 /II/22/23	1.52E-05	-	-	7.27E-07	7.58E-05	-	-	3.63E-06	1.52E-04	-	-	7.27E-06	2.27E-04	-	-	1.09E-05	3.03E-04	-	-	1.45E-05
53	C67 /II/22/23	1.13E-05	-	2.38E-06	6.25E-07	5.66E-05	-	1.19E-05	3.12E-06	1.13E-04	-	2.38E-05	6.25E-06	1.70E-04	-	3.57E-05	9.37E-06	2.27E-04	-	4.75E-05	1.25E-05
54	C68 /II/22/23	9.54E-06	-	-	5.34E-07	4.77E-05	-	-	2.67E-06	9.54E-05	-	-	5.34E-06	1.43E-04	-	-	8.01E-06	1.91E-04	-	-	1.07E-05
55	C69 /II/22/23	9.84E-06	-	2.65E-06	5.16E-07	4.92E-05	-	1.33E-05	2.58E-06	9.84E-05	-	2.65E-05	5.16E-06	1.48E-04	-	3.98E-05	7.73E-06	1.97E-04	-	5.31E-05	1.03E-05

56	C70 /II/22/23	9.65E-06	-	-	5.93E-07	4.82E-05	-	-	2.96E-06	9.65E-05	-	-	5.93E-06	1.45E-04	-	-	8.89E-06	1.93E-04	-	-	1.19E-05
57	C128 /II/22/23	1.35E-05	1.38E-03	-	5.34E-07	6.74E-05	6.88E-03	-	2.67E-06	1.35E-04	1.38E-02	-	5.34E-06	2.02E-04	2.06E-02	-	8.01E-06	2.70E-04	2.75E-02	-	1.07E-05
58	C129 /II/22/23	1.14E-05	7.13E-04	-	5.87E-07	5.68E-05	3.56E-03	-	2.94E-06	1.14E-04	7.13E-03	-	5.87E-06	1.70E-04	1.07E-02	-	8.81E-06	2.27E-04	1.43E-02	-	1.17E-05
59	C130 /II/22/23	9.42E-06	3.51E-04	-	5.45E-07	4.71E-05	1.76E-03	-	2.72E-06	9.42E-05	3.51E-03	-	5.45E-06	1.41E-04	5.27E-03	-	8.17E-06	1.88E-04	7.02E-03	-	1.09E-05
60	C131 /II/22/23	1.01E-05	1.00E-04	-	8.10E-07	5.05E-05	5.00E-04	-	4.05E-06	1.01E-04	1.00E-03	-	8.10E-06	1.52E-04	1.50E-03	-	1.22E-05	2.02E-04	2.00E-03	-	1.62E-05
61	C133 /II/22/23	9.74E-06	5.76E-05	-	5.45E-07	4.87E-05	2.88E-04	-	2.73E-06	9.74E-05	5.76E-04	-	5.45E-06	1.46E-04	8.64E-04	-	8.18E-06	1.95E-04	1.15E-03	-	1.09E-05
62	C134 /II/22/23	1.26E-05	1.16E-04	-	6.15E-07	6.29E-05	5.82E-04	-	3.07E-06	1.26E-04	1.16E-03	-	6.15E-06	1.89E-04	1.75E-03	-	9.22E-06	2.52E-04	2.33E-03	-	1.23E-05
63	C135 /II/22/23	9.68E-06	4.97E-05	-	4.78E-07	4.84E-05	2.48E-04	-	2.39E-06	9.68E-05	4.97E-04	-	4.78E-06	1.45E-04	7.45E-04	-	7.16E-06	1.94E-04	9.93E-04	-	9.55E-06
64	C136 /II/22/23	1.01E-05	3.09E-05	5.06E-06	8.57E-07	5.03E-05	1.54E-04	2.53E-05	4.28E-06	1.01E-04	3.09E-04	5.06E-05	8.57E-06	1.51E-04	4.63E-04	7.58E-05	1.29E-05	2.01E-04	6.18E-04	1.01E-04	1.71E-05
65	C137 /II/22/23	9.89E-06	1.07E-05	3.89E-06	4.06E-07	4.94E-05	5.36E-05	1.94E-05	2.03E-06	9.89E-05	1.07E-04	3.89E-05	4.06E-06	1.48E-04	1.61E-04	5.83E-05	6.09E-06	1.98E-04	2.14E-04	7.77E-05	8.11E-06
66	C138 /II/22/23	1.07E-05	1.30E-05	5.90E-06	8.56E-07	5.37E-05	6.52E-05	2.95E-05	4.28E-06	1.07E-04	1.30E-04	5.90E-05	8.56E-06	1.61E-04	1.96E-04	8.86E-05	1.28E-05	2.15E-04	2.61E-04	1.18E-04	1.71E-05
67	C139 /II/22/23	1.19E-05	7.99E-06	-	5.03E-07	5.97E-05	4.00E-05	-	2.51E-06	1.19E-04	7.99E-05	-	5.03E-06	1.79E-04	1.20E-04	-	7.54E-06	2.39E-04	1.60E-04	-	1.01E-05
68	C140 /II/22/23	8.67E-06	6.20E-06	-	4.46E-07	4.34E-05	3.10E-05	-	2.23E-06	8.67E-05	6.20E-05	-	4.46E-06	1.30E-04	9.31E-05	-	6.68E-06	1.73E-04	1.24E-04	-	8.91E-06
69	C141 /II/22/23	9.20E-06	6.73E-06	-	4.36E-07	4.60E-05	3.36E-05	-	2.18E-06	9.20E-05	6.73E-05	-	4.36E-06	1.38E-04	1.01E-04	-	6.54E-06	1.84E-04	1.35E-04	-	8.72E-06
70	C142 /II/22/23	9.31E-06	1.58E-05	-	5.44E-07	4.66E-05	7.91E-05	-	2.72E-06	9.31E-05	1.58E-04	-	5.44E-06	1.40E-04	2.37E-04	-	8.16E-06	1.86E-04	3.16E-04	-	1.09E-05
71	C143 /II/22/23	1.22E-05	-	-	6.83E-07	6.10E-05	-	-	3.41E-06	1.22E-04	-	-	6.83E-06	1.83E-04	-	-	1.02E-05	2.44E-04	-	-	1.37E-05
72	C144 /II/22/23	9.92E-06	2.02E-05	-	5.41E-07	4.96E-05	1.01E-04	-	2.70E-06	9.92E-05	2.02E-04	-	5.41E-06	1.49E-04	3.03E-04	-	8.11E-06	1.98E-04	4.04E-04	-	1.08E-05
73	C145 /II/22/23	1.19E-05	5.62E-06	-	8.91E-07	5.93E-05	2.81E-05	-	4.45E-06	1.19E-04	5.62E-05	-	8.91E-06	1.78E-04	8.43E-05	-	1.34E-05	2.37E-04	1.12E-04	-	1.78E-05
74	C146 /II/22/23	1.50E-05	1.22E-05	1.41E-06	7.66E-07	7.51E-05	6.10E-05	7.04E-06	3.83E-06	1.50E-04	1.22E-04	1.41E-05	7.66E-06	2.25E-04	1.83E-04	2.11E-05	1.15E-05	3.00E-04	2.44E-04	2.81E-05	1.53E-05

75	C147 /II/22/23	1.22E-05	4.00E-06	-	5.15E-07	6.08E-05	2.00E-05	-	2.58E-06	1.22E-04	4.00E-05	-	5.15E-06	1.82E-04	5.99E-05	-	7.73E-06	2.43E-04	7.99E-05	-	1.03E-05
76	C148 /II/22/23	1.27E-05	3.43E-06	-	6.34E-07	6.35E-05	1.71E-05	-	3.17E-06	1.27E-04	3.43E-05	-	6.34E-06	1.91E-04	5.14E-05	-	9.50E-06	2.54E-04	6.85E-05	-	1.27E-05
77	C149 /II/22/23	1.21E-05	-	-	7.37E-07	6.07E-05	-	-	3.68E-06	1.21E-04	-	-	7.37E-06	1.82E-04	-	-	1.10E-05	2.43E-04	-	-	1.47E-05
78	C15 /II/22/23	1.00E-05	8.63E-06	-	7.43E-07	5.02E-05	4.32E-05	-	3.72E-06	1.00E-04	8.63E-05	-	7.43E-06	1.51E-04	1.29E-04	-	1.12E-05	2.01E-04	1.73E-04	-	1.49E-05
79	C86 /II/21/22	1.18E-05	3.08E-07	-	7.80E-07	5.90E-05	1.54E-06	-	3.90E-06	1.18E-04	3.08E-06	-	7.80E-06	1.77E-04	4.63E-06	-	1.17E-05	2.36E-04	6.17E-06	-	1.56E-05
80	C87 /II/21/22	9.62E-06	-	-	5.55E-07	4.81E-05	-	-	2.78E-06	9.62E-05	-	-	5.55E-06	1.44E-04	-	-	8.33E-06	1.92E-04	-	-	1.11E-05
81	C88 /II/21/22	1.01E-05	1.92E-07	-	7.67E-07	5.06E-05	9.62E-07	-	3.83E-06	1.01E-04	1.92E-06	-	7.67E-06	1.52E-04	2.89E-06	-	1.15E-05	2.02E-04	3.85E-06	-	1.53E-05
82	C89 /II/21/22	9.91E-06	2.11E-07	1.48E-06	4.01E-07	4.96E-05	1.05E-06	7.39E-06	2.01E-06	9.91E-05	2.11E-06	1.48E-05	4.01E-06	1.49E-04	3.16E-06	2.22E-05	6.02E-06	1.98E-04	4.21E-06	2.96E-05	8.03E-06
83	C90 /II/21/22	9.82E-06	2.10E-07	2.18E-06	8.05E-07	4.91E-05	1.05E-06	1.09E-05	4.02E-06	9.82E-05	2.10E-06	2.18E-05	8.05E-06	1.47E-04	3.15E-06	3.27E-05	1.21E-05	1.96E-04	4.20E-06	4.37E-05	1.61E-05
84	C91 /II/21/22	1.01E-05	-	-	7.37E-07	5.06E-05	-	-	3.69E-06	1.01E-04	-	-	7.37E-06	1.52E-04	-	-	1.11E-05	2.03E-04	-	-	1.47E-05
85	C92 /II/21/22	8.84E-06	2.47E-07	-	7.53E-07	4.42E-05	1.23E-06	-	3.76E-06	8.84E-05	2.47E-06	-	7.53E-06	1.33E-04	3.70E-06	-	1.13E-05	1.77E-04	4.93E-06	-	1.51E-05
86	C93 /II/21/22	8.56E-06	1.55E-07	-	4.07E-07	4.28E-05	7.77E-07	-	2.04E-06	8.56E-05	1.55E-06	-	4.07E-06	1.28E-04	2.33E-06	-	6.11E-06	1.71E-04	3.11E-06	-	8.15E-06
87	C94 /II/21/22	7.87E-06	1.81E-07	1.49E-06	3.81E-07	3.93E-05	9.05E-07	7.46E-06	1.91E-06	7.87E-05	1.81E-06	1.49E-05	3.81E-06	1.18E-04	2.71E-06	2.24E-05	5.72E-06	1.57E-04	3.62E-06	2.99E-05	7.63E-06
88	C95 /II/21/22	8.63E-06	1.63E-07	-	3.25E-07	4.32E-05	8.15E-07	-	1.63E-06	8.63E-05	1.63E-06	-	3.25E-06	1.30E-04	2.44E-06	-	4.88E-06	1.73E-04	3.26E-06	-	6.51E-06
89	C96 /II/21/22	9.20E-06	-	-	4.88E-07	4.60E-05	-	-	2.44E-06	9.20E-05	-	-	4.88E-06	1.38E-04	-	-	7.31E-06	1.84E-04	-	-	9.75E-06
90	C97 /II/21/22	9.77E-06	-	1.57E-06	5.27E-07	4.88E-05	-	7.85E-06	2.64E-06	9.77E-05	-	1.57E-05	5.27E-06	1.47E-04	-	2.35E-05	7.91E-06	1.95E-04	-	3.14E-05	1.05E-05
91	C98 /II/21/22	1.88E-05	-	-	5.04E-07	9.42E-05	-	-	2.52E-06	1.88E-04	-	-	5.04E-06	2.83E-04	-	-	7.56E-06	3.77E-04	-	-	1.01E-05
92	C99 /II/21/22	1.43E-05	2.12E-07	1.47E-06	8.21E-07	7.13E-05	1.06E-06	7.33E-06	4.10E-06	1.43E-04	2.12E-06	1.47E-05	8.21E-06	2.14E-04	3.18E-06	2.20E-05	1.23E-05	2.85E-04	4.25E-06	2.93E-05	1.64E-05
93	C101 /II/21/22	1.13E-05	2.34E-07	1.65E-06	8.33E-07	5.67E-05	1.17E-06	8.23E-06	4.17E-06	1.13E-04	2.34E-06	1.65E-05	8.33E-06	1.70E-04	3.51E-06	2.47E-05	1.25E-05	2.27E-04	4.68E-06	3.29E-05	1.67E-05

94	C16 /II/21/22	8.28E-06	-	1.88E-06	6.11E-07	4.14E-05	-	9.42E-06	3.06E-06	8.28E-05	-	1.88E-05	6.11E-06	1.24E-04	-	2.83E-05	9.17E-06	1.66E-04	-	3.77E-05	1.22E-05
95	C17 /II/21/22	8.01E-06	3.28E-07	-	1.07E-06	4.00E-05	1.64E-06	-	5.34E-06	8.01E-05	3.28E-06	-	1.07E-05	1.20E-04	4.92E-06	-	1.60E-05	1.60E-04	6.56E-06	-	2.13E-05
96	C18 /II/21/22	7.97E-06	1.32E-07	1.30E-05	7.95E-07	3.98E-05	6.58E-07	6.51E-05	3.98E-06	7.97E-05	1.32E-06	1.30E-04	7.95E-06	1.20E-04	1.98E-06	1.95E-04	1.19E-05	1.59E-04	2.63E-06	2.60E-04	1.59E-05
97	C21 /II/21/22	4.95E-06	-	2.46E-06	1.01E-06	2.48E-05	-	1.23E-05	5.06E-06	4.95E-05	-	2.46E-05	1.01E-05	7.43E-05	-	3.70E-05	1.52E-05	9.91E-05	-	4.93E-05	2.02E-05
98	C39 /II/21/22	9.90E-06	-	2.68E-06	7.09E-07	4.95E-05	-	1.34E-05	3.54E-06	9.90E-05	-	2.68E-05	7.09E-06	1.48E-04	-	4.03E-05	1.06E-05	1.98E-04	-	5.37E-05	1.42E-05
99	C40 /II/21/22	8.71E-06	-	2.42E-06	8.93E-07	4.36E-05	-	1.21E-05	4.47E-06	8.71E-05	-	2.42E-05	8.93E-06	1.31E-04	-	3.63E-05	1.34E-05	1.74E-04	-	4.84E-05	1.79E-05
100	C41 /II/21/22	1.17E-05	2.78E-07	2.16E-06	7.18E-07	5.87E-05	1.39E-06	1.08E-05	3.59E-06	1.17E-04	2.78E-06	2.16E-05	7.18E-06	1.76E-04	4.17E-06	3.24E-05	1.08E-05	2.35E-04	5.56E-06	4.32E-05	1.44E-05
101	C43 /II/21/22	1.94E-05	1.41E-07	5.41E-06	9.20E-07	9.72E-05	7.05E-07	2.70E-05	4.60E-06	1.94E-04	1.41E-06	5.41E-05	9.20E-06	2.92E-04	2.11E-06	8.11E-05	1.38E-05	3.89E-04	2.82E-06	1.08E-04	1.84E-05
102	C44 /II/21/22	7.80E-05	-	2.10E-06	9.78E-07	3.90E-04	-	1.05E-05	4.89E-06	7.80E-04	-	2.10E-05	9.78E-06	1.17E-03	-	3.15E-05	1.47E-05	1.56E-03	-	4.20E-05	1.96E-05
103	C46 /II/21/22	1.23E-05	-	3.55E-06	8.54E-07	6.17E-05	-	1.78E-05	4.27E-06	1.23E-04	-	3.55E-05	8.54E-06	1.85E-04	-	5.33E-05	1.28E-05	2.47E-04	-	7.11E-05	1.71E-05
104	C314 /II/17/18	-	1.11E-04	-	7.22E-06	-	5.54E-04	-	3.61E-05	-	1.11E-03	-	7.22E-05	-	1.66E-03	-	1.08E-04	-	2.22E-03	-	1.44E-04
105	C315 /II/17/18	-	1.21E-05	-	4.22E-06	-	6.05E-05	-	2.11E-05	-	1.21E-04	-	4.22E-05	-	1.81E-04	-	6.33E-05	-	2.42E-04	-	8.45E-05
106	C319 /II/17/18	-	6.81E-06	-	4.72E-07	-	3.41E-05	-	2.36E-06	-	6.81E-05	-	4.72E-06	-	1.02E-04	-	7.08E-06	-	1.36E-04	-	9.44E-06
107	C320 /II/17/18	-	3.88E-06	-	2.41E-06	-	1.94E-05	-	1.21E-05	-	3.88E-05	-	2.41E-05	-	5.82E-05	-	3.62E-05	-	7.75E-05	-	4.82E-05
108	C321 /II/17/18	-	1.33E-05	-	2.42E-06	-	6.64E-05	-	1.21E-05	-	1.33E-04	-	2.42E-05	-	1.99E-04	-	3.63E-05	-	2.66E-04	-	4.84E-05
109	C322 /II/17/18	-	1.41E-05	-	9.10E-07	-	7.04E-05	-	4.55E-06	-	1.41E-04	-	9.10E-06	-	2.11E-04	-	1.37E-05	-	2.82E-04	-	1.82E-05
110	C323 /II/17/18	3.37E-05	-	4.37E-06	2.44E-07	1.68E-04	-	2.18E-05	1.22E-06	3.37E-04	-	4.37E-05	2.44E-06	5.05E-04	-	6.55E-05	3.67E-06	6.73E-04	-	8.73E-05	4.89E-06
111	C324 /II/17/18	-	1.15E-05	-	2.52E-06	-	5.77E-05	-	1.26E-05	-	1.15E-04	-	2.52E-05	-	1.73E-04	-	3.78E-05	-	2.31E-04	-	5.04E-05

112	C35 /I/23/24	7.40E-06	1.56E-06	-	8.26E-07	3.70E-05	7.81E-06	-	4.13E-06	7.40E-05	1.56E-05	-	8.26E-06	1.11E-04	2.34E-05	-	1.24E-05	1.48E-04	3.12E-05	-	1.65E-05
113	C168 /I/22/23	8.30E-06	-	-	3.91E-07	4.15E-05	-	-	1.96E-06	8.30E-05	-	-	3.91E-06	1.25E-04	-	-	5.87E-06	1.66E-04	-	-	7.83E-06
114	C175 /I/22/23	8.24E-06	2.35E-07	-	5.65E-07	4.12E-05	1.18E-06	-	2.82E-06	8.24E-05	2.35E-06	-	5.65E-06	1.24E-04	3.53E-06	-	8.47E-06	1.65E-04	4.71E-06	-	1.13E-05
115	C100 /II/21/22	5.29E-06	1.56E-07	-	1.26E-06	2.64E-05	7.78E-07	-	6.31E-06	5.29E-05	1.56E-06	-	1.26E-05	7.93E-05	2.33E-06	-	1.89E-05	1.06E-04	3.11E-06	-	2.53E-05
116	C20 /II/21/22	7.58E-06	1.81E-07	3.47E-06	9.15E-07	3.79E-05	9.03E-07	1.74E-05	4.58E-06	7.58E-05	1.81E-06	3.47E-05	9.15E-06	1.14E-04	2.71E-06	5.21E-05	1.37E-05	1.52E-04	3.61E-06	6.95E-05	1.83E-05
117	C316 /II/17/18	-	7.13E-06	-	8.09E-07	-	3.57E-05	-	4.05E-06	-	7.13E-05	-	8.09E-06	-	1.07E-04	-	1.21E-05	-	1.43E-04	-	1.62E-05
118	C317 /II/17/18	-	9.47E-06	-	5.23E-07	-	4.73E-05	-	2.61E-06	-	9.47E-05	-	5.23E-06	-	1.42E-04	-	7.84E-06	-	1.89E-04	-	1.05E-05
119	C326 /II/17/18	2.83E-06	6.36E-06	4.62E-06	3.41E-06	1.42E-05	3.18E-05	2.31E-05	1.71E-05	2.83E-05	6.36E-05	4.62E-05	3.41E-05	4.25E-05	9.53E-05	6.93E-05	5.12E-05	5.67E-05	1.27E-04	9.24E-05	6.83E-05

Table S6. Hazard Quotient (HQ) for ingestion exposure to copper, lead, nickel, and cadmium contained in the cigarettes tested.

No.	Sample code	1 cigarette per day				5 cigarettes per day				10 cigarettes per day				15 cigarettes per day				20 cigarettes per day			
		Cu	Pb	Ni	Cd	Cu	Pb	Ni	Cd	Cu	Pb	Ni	Cd	Cu	Pb	Ni	Cd	Cu	Pb	Ni	Cd
1	C20 /I/23/24	1.90E-04	4.09E-04	-	6.97E-04	9.48E-04	2.05E-03	-	3.49E-03	1.90E-03	4.09E-03	-	6.97E-03	2.84E-03	6.14E-03	-	1.05E-02	3.79E-03	8.19E-03	-	1.39E-02
2	C21 /I/23/24	1.90E-04	1.38E-04	-	5.30E-04	9.51E-04	6.88E-04	-	2.65E-03	1.90E-03	1.38E-03	-	5.30E-03	2.85E-03	2.06E-03	-	7.95E-03	3.81E-03	2.75E-03	-	1.06E-02
3	C22 /I/23/24	1.54E-04	3.26E-04	-	6.60E-04	7.71E-04	1.63E-03	-	3.30E-03	1.54E-03	3.26E-03	-	6.60E-03	2.31E-03	4.89E-03	-	9.90E-03	3.08E-03	6.52E-03	-	1.32E-02
4	C23 /I/23/24	1.70E-04	2.98E-04	-	7.76E-04	8.48E-04	1.49E-03	-	3.88E-03	1.70E-03	2.98E-03	-	7.76E-03	2.54E-03	4.47E-03	-	1.16E-02	3.39E-03	5.96E-03	-	1.55E-02
5	C24 /I/23/24	1.66E-04	3.46E-05	-	6.22E-04	8.29E-04	1.73E-04	-	3.11E-03	1.66E-03	3.46E-04	-	6.22E-03	2.49E-03	5.19E-04	-	9.33E-03	3.31E-03	6.93E-04	-	1.24E-02
6	C25 /I/23/24	1.52E-04	2.72E-04	-	5.81E-04	7.58E-04	1.36E-03	-	2.90E-03	1.52E-03	2.72E-03	-	5.81E-03	2.28E-03	4.08E-03	-	8.71E-03	3.03E-03	5.44E-03	-	1.16E-02
7	C26 /I/23/24	1.65E-04	2.77E-04	-	1.22E-03	8.24E-04	1.38E-03	-	6.10E-03	1.65E-03	2.77E-03	-	1.22E-02	2.47E-03	4.15E-03	-	1.83E-02	3.30E-03	5.53E-03	-	2.44E-02
8	C27 /I/23/24	2.12E-04	1.61E-04	-	9.33E-04	1.06E-03	8.06E-04	-	4.66E-03	2.12E-03	1.61E-03	-	9.33E-03	3.17E-03	2.42E-03	-	1.40E-02	4.23E-03	3.23E-03	-	1.87E-02
9	C28 /I/23/24	1.72E-04	2.24E-04	-	6.26E-04	8.58E-04	1.12E-03	-	3.13E-03	1.72E-03	2.24E-03	-	6.26E-03	2.57E-03	3.36E-03	-	9.39E-03	3.43E-03	4.48E-03	-	1.25E-02
10	C29 /I/23/24	2.08E-04	1.34E-04	-	9.17E-04	1.04E-03	6.70E-04	-	4.58E-03	2.08E-03	1.34E-03	-	9.17E-03	3.12E-03	2.01E-03	-	1.37E-02	4.16E-03	2.68E-03	-	1.83E-02
11	C30 /I/23/24	1.98E-04	-	-	7.86E-04	9.92E-04	-	-	3.93E-03	1.98E-03	-	-	7.86E-03	2.98E-03	-	-	1.18E-02	3.97E-03	-	-	1.57E-02
12	C31 /I/23/24	1.60E-04	2.96E-04	-	5.97E-04	8.00E-04	1.48E-03	-	2.98E-03	1.60E-03	2.96E-03	-	5.97E-03	2.40E-03	4.44E-03	-	8.95E-03	3.20E-03	5.92E-03	-	1.19E-02
13	C32 /I/23/24	2.02E-04	2.98E-04	-	8.71E-04	1.01E-03	1.49E-03	-	4.36E-03	2.02E-03	2.98E-03	-	8.71E-03	3.03E-03	4.47E-03	-	1.31E-02	4.05E-03	5.96E-03	-	1.74E-02
14	C33 /I/23/24	1.41E-04	2.41E-04	-	6.73E-04	7.06E-04	1.20E-03	-	3.37E-03	1.41E-03	2.41E-03	-	6.73E-03	2.12E-03	3.61E-03	-	1.01E-02	2.82E-03	4.82E-03	-	1.35E-02
15	C34 /I/23/24	2.02E-04	1.68E-04	-	7.60E-04	1.01E-03	8.41E-04	-	3.80E-03	2.02E-03	1.68E-03	-	7.60E-03	3.03E-03	2.52E-03	-	1.14E-02	4.04E-03	3.36E-03	-	1.52E-02
16	C36 /I/23/24	2.30E-04	3.02E-04	-	8.17E-04	1.15E-03	1.51E-03	-	4.09E-03	2.30E-03	3.02E-03	-	8.17E-03	3.45E-03	4.54E-03	-	1.23E-02	4.60E-03	6.05E-03	-	1.63E-02
17	C37 /I/23/24	2.00E-04	5.92E-04	-	9.26E-04	9.98E-04	2.96E-03	-	4.63E-03	2.00E-03	5.92E-03	-	9.26E-03	3.00E-03	8.88E-03	-	1.39E-02	3.99E-03	1.18E-02	-	1.85E-02

18	C38 /I/23/24	1.58E-04	3.77E-04	-	7.96E-04	7.90E-04	1.89E-03	-	3.98E-03	1.58E-03	3.77E-03	-	7.96E-03	2.37E-03	5.66E-03	-	1.19E-02	3.16E-03	7.55E-03	-	1.59E-02
19	C39 /I/23/24	2.12E-04	2.13E-04	-	5.47E-04	1.06E-03	1.07E-03	-	2.73E-03	2.12E-03	2.13E-03	-	5.47E-03	3.19E-03	3.20E-03	-	8.20E-03	4.25E-03	4.26E-03	-	1.09E-02
20	C128 /I/22/23	2.14E-04	4.16E-05	9.94E-05	6.15E-04	1.07E-03	2.08E-04	4.97E-04	3.08E-03	2.14E-03	4.16E-04	4.97E-04	6.15E-03	3.22E-03	6.23E-04	2.49E-02	9.23E-03	4.29E-03	8.31E-04	1.99E-03	1.23E-02
21	C129 /I/22/23	2.30E-04	-	9.82E-05	3.88E-04	1.15E-03	-	4.91E-04	1.94E-03	2.30E-03	-	4.91E-04	3.88E-03	3.46E-03	-	2.46E-02	5.82E-03	4.61E-03	-	1.96E-03	7.75E-03
22	C130 /I/22/23	2.84E-04	7.28E-05	9.68E-05	4.24E-04	1.42E-03	3.64E-04	4.84E-04	2.12E-03	2.84E-03	7.28E-04	4.84E-04	4.24E-03	4.26E-03	1.09E-03	2.42E-02	6.36E-03	5.68E-03	1.46E-03	1.94E-03	8.47E-03
23	C131 /I/22/23	1.77E-04	3.34E-05	1.94E-04	3.22E-04	8.83E-04	1.67E-04	9.68E-04	1.61E-03	1.77E-03	3.34E-04	9.68E-04	3.22E-03	2.65E-03	5.00E-04	4.84E-02	4.83E-03	3.53E-03	6.67E-04	3.87E-03	6.43E-03
24	C132 /I/22/23	2.26E-04	8.02E-05	4.02E-04	3.13E-03	1.13E-03	4.01E-04	2.01E-03	1.56E-02	2.26E-03	8.02E-04	2.01E-03	3.13E-02	3.40E-03	1.20E-03	1.01E-01	4.69E-02	4.53E-03	1.60E-03	8.05E-03	6.25E-02
25	C133 /I/22/23	2.73E-04	-	3.38E-04	3.88E-04	1.37E-03	-	1.69E-03	1.94E-03	2.73E-03	-	1.69E-03	3.88E-03	4.10E-03	-	8.45E-02	5.83E-03	5.47E-03	-	6.76E-03	7.77E-03
26	C134 /I/22/23	2.37E-04	-	-	9.06E-04	1.19E-03	-	-	4.53E-03	2.37E-03	-	-	9.06E-03	3.56E-03	-	-	1.36E-02	4.74E-03	-	-	1.81E-02
27	C135 /I/22/23	2.01E-04	-	2.50E-04	5.35E-04	1.01E-03	-	1.25E-03	2.67E-03	2.01E-03	-	1.25E-03	5.35E-03	3.02E-03	-	6.26E-02	8.02E-03	4.03E-03	-	5.01E-03	1.07E-02
28	C136 /I/22/23	2.16E-04	-	4.45E-04	9.14E-04	1.08E-03	-	2.22E-03	4.57E-03	2.16E-03	-	2.22E-03	9.14E-03	3.25E-03	-	1.11E-01	1.37E-02	4.33E-03	-	8.90E-03	1.83E-02
29	C137 /I/22/23	1.99E-04	5.33E-05	4.93E-04	3.68E-04	9.96E-04	2.67E-04	2.47E-03	1.84E-03	1.99E-03	5.33E-04	2.47E-03	3.68E-03	2.99E-03	8.00E-04	1.23E-01	5.52E-03	3.98E-03	1.07E-03	9.87E-03	7.36E-03
30	C138 /I/22/23	2.25E-04	-	2.55E-04	4.55E-04	1.13E-03	-	1.27E-03	2.28E-03	2.25E-03	-	1.27E-03	4.55E-03	3.38E-03	-	6.36E-02	6.83E-03	4.51E-03	-	5.09E-03	9.11E-03
31	C169 /I/22/23	2.07E-04	7.24E-05	-	1.16E-03	1.03E-03	3.62E-04	-	5.81E-03	2.07E-03	7.24E-04	-	1.16E-02	3.10E-03	1.09E-03	-	1.74E-02	4.13E-03	1.45E-03	-	2.32E-02
32	C170 /I/22/23	2.39E-04	-	1.05E-04	4.80E-04	1.19E-03	-	5.24E-04	2.40E-03	2.39E-03	-	5.24E-04	4.80E-03	3.58E-03	-	2.62E-02	7.20E-03	4.78E-03	-	2.10E-03	9.61E-03
33	C171 /I/22/23	2.20E-04	-	8.35E-05	5.91E-04	1.10E-03	-	4.17E-04	2.95E-03	2.20E-03	-	4.17E-04	5.91E-03	3.30E-03	-	2.09E-02	8.86E-03	4.40E-03	-	1.67E-03	1.18E-02
34	C172 /I/22/23	2.41E-04	-	-	4.74E-04	1.20E-03	-	-	2.37E-03	2.41E-03	-	-	4.74E-03	3.61E-03	-	-	7.11E-03	4.81E-03	-	-	9.48E-03
35	C173 /I/22/23	2.35E-04	-	1.02E-04	5.42E-04	1.17E-03	-	5.11E-04	2.71E-03	2.35E-03	-	5.11E-04	5.42E-03	3.52E-03	-	2.56E-02	8.13E-03	4.70E-03	-	2.05E-03	1.08E-02
36	C174 /I/22/23	2.68E-04	-	1.06E-04	7.77E-04	1.34E-03	-	5.28E-04	3.89E-03	2.68E-03	-	5.28E-04	7.77E-03	4.02E-03	-	2.64E-02	1.17E-02	5.36E-03	-	2.11E-03	1.55E-02

37	C176 /I/22/23	1.76E-04	4.73E-05	1.02E-04	7.06E-04	8.80E-04	2.36E-04	5.12E-04	3.53E-03	1.76E-03	4.73E-04	5.12E-04	7.06E-03	2.64E-03	7.09E-04	2.56E-02	1.06E-02	3.52E-03	9.46E-04	2.05E-03	1.41E-02
38	C177 /I/22/23	2.15E-04	-	7.21E-05	7.10E-04	1.07E-03	-	3.60E-04	3.55E-03	2.15E-03	-	3.60E-04	7.10E-03	3.22E-03	-	1.80E-02	1.07E-02	4.30E-03	-	1.44E-03	1.42E-02
39	C178 /I/22/23	2.44E-04	6.31E-05	8.20E-05	8.77E-04	1.22E-03	3.15E-04	4.10E-04	4.39E-03	2.44E-03	6.31E-04	4.10E-04	8.77E-03	3.65E-03	9.46E-04	2.05E-02	1.32E-02	4.87E-03	1.26E-03	1.64E-03	1.75E-02
40	C179 /I/22/23	2.04E-04	-	-	6.32E-04	1.02E-03	-	-	3.16E-03	2.04E-03	-	-	6.32E-03	3.06E-03	-	-	9.48E-03	4.07E-03	-	-	1.26E-02
41	C180 /I/22/23	1.86E-04	-	1.25E-04	6.05E-04	9.30E-04	-	6.23E-04	3.02E-03	1.86E-03	-	6.23E-04	6.05E-03	2.79E-03	-	3.11E-02	9.07E-03	3.72E-03	-	2.49E-03	1.21E-02
42	C181 /I/22/23	2.11E-04	3.50E-05	1.07E-04	4.03E-04	1.05E-03	1.75E-04	5.35E-04	2.01E-03	2.11E-03	3.50E-04	5.35E-04	4.03E-03	3.16E-03	5.24E-04	2.68E-02	6.04E-03	4.22E-03	6.99E-04	2.14E-03	8.06E-03
43	C182 /I/22/23	2.36E-04	6.07E-05	9.23E-05	5.21E-04	1.18E-03	3.04E-04	4.62E-04	2.60E-03	2.36E-03	6.07E-04	4.62E-04	5.21E-03	3.54E-03	9.11E-04	2.31E-02	7.81E-03	4.72E-03	1.21E-03	1.85E-03	1.04E-02
44	C183 /I/22/23	2.78E-04	6.50E-05	-	6.04E-04	1.39E-03	3.25E-04	-	3.02E-03	2.78E-03	6.50E-04	-	6.04E-03	4.17E-03	9.75E-04	-	9.06E-03	5.56E-03	1.30E-03	-	1.21E-02
45	C184 /I/22/23	2.04E-04	9.00E-05	-	3.41E-04	1.02E-03	4.50E-04	-	1.71E-03	2.04E-03	9.00E-04	-	3.41E-03	3.06E-03	1.35E-03	-	5.12E-03	4.08E-03	1.80E-03	-	6.82E-03
46	C185 /I/22/23	2.45E-04	3.42E-05	-	2.61E-04	1.23E-03	1.71E-04	-	1.31E-03	2.45E-03	3.42E-04	-	2.61E-03	3.68E-03	5.14E-04	-	3.92E-03	4.91E-03	6.85E-04	-	5.23E-03
47	C186 /I/22/23	1.89E-04	-	9.35E-05	3.48E-04	9.43E-04	-	4.68E-04	1.74E-03	1.89E-03	-	4.68E-04	3.48E-03	2.83E-03	-	2.34E-02	5.23E-03	3.77E-03	-	1.87E-03	6.97E-03
48	C187 /I/22/23	2.92E-04	-	8.17E-05	4.61E-04	1.46E-03	-	4.08E-04	2.31E-03	2.92E-03	-	4.08E-04	4.61E-03	4.39E-03	-	2.04E-02	6.92E-03	5.85E-03	-	1.63E-03	9.22E-03
49	C188 /I/22/23	2.17E-04	1.31E-04	1.09E-04	2.96E-04	1.09E-03	6.56E-04	5.43E-04	1.48E-03	2.17E-03	1.31E-03	5.43E-04	2.96E-03	3.26E-03	1.97E-03	2.72E-02	4.44E-03	4.34E-03	2.62E-03	2.17E-03	5.92E-03
50	C64 /II/22/23	3.92E-04	-	9.70E-05	7.05E-04	1.96E-03	-	4.85E-04	3.53E-03	3.92E-03	-	4.85E-04	7.05E-03	5.87E-03	-	2.42E-02	1.06E-02	7.83E-03	-	1.94E-03	1.41E-02
51	C65 /II/22/23	2.77E-04	-	4.42E-04	6.39E-04	1.38E-03	-	2.21E-03	3.19E-03	2.77E-03	-	2.21E-03	6.39E-03	4.15E-03	-	1.11E-01	9.58E-03	5.53E-03	-	8.85E-03	1.28E-02
52	C66 /II/22/23	3.79E-04	-	-	7.27E-04	1.89E-03	-	-	3.63E-03	3.79E-03	-	-	7.27E-03	5.68E-03	-	-	1.09E-02	7.58E-03	-	-	1.45E-02
53	C67 /II/22/23	2.83E-04	-	1.19E-04	6.25E-04	1.42E-03	-	5.94E-04	3.12E-03	2.83E-03	-	5.94E-04	6.25E-03	4.25E-03	-	2.97E-02	9.37E-03	5.66E-03	-	2.38E-03	1.25E-02
54	C68 /II/22/23	2.38E-04	-	-	5.34E-04	1.19E-03	-	-	2.67E-03	2.38E-03	-	-	5.34E-03	3.58E-03	-	-	8.01E-03	4.77E-03	-	-	1.07E-02
55	C69 /II/22/23	2.46E-04	-	1.33E-04	5.16E-04	1.23E-03	-	6.64E-04	2.58E-03	2.46E-03	-	6.64E-04	5.16E-03	3.69E-03	-	3.32E-02	7.73E-03	4.92E-03	-	2.65E-03	1.03E-02

56	C70 /II/22/23	2.41E-04	-	-	5.93E-04	1.21E-03	-	-	2.96E-03	2.41E-03	-	-	5.93E-03	3.62E-03	-	-	8.89E-03	4.82E-03	-	-	1.19E-02
57	C128 /II/22/23	3.37E-04	3.44E-01	-	5.34E-04	1.69E-03	**1.72E+00**	-	2.67E-03	3.37E-03	**3.44E+00**	-	5.34E-03	5.06E-03	**5.16E+00**	-	8.01E-03	6.74E-03	**6.88E+00**	-	1.07E-02
58	C129 /II/22/23	2.84E-04	1.78E-01	-	5.87E-04	1.42E-03	8.91E-01	-	2.94E-03	2.84E-03	**1.78E+00**	-	5.87E-03	4.26E-03	**2.67E+00**	-	8.81E-03	5.68E-03	**3.56E+00**	-	1.17E-02
59	C130 /II/22/23	2.35E-04	8.78E-02	-	5.45E-04	1.18E-03	4.39E-01	-	2.72E-03	2.35E-03	8.78E-01	-	5.45E-03	3.53E-03	**1.32E+00**	-	8.17E-03	4.71E-03	**1.76E+00**	-	1.09E-02
60	C131 /II/22/23	2.53E-04	2.50E-02	-	8.10E-04	1.26E-03	1.25E-01	-	4.05E-03	2.53E-03	2.50E-01	-	8.10E-03	3.79E-03	3.75E-01	-	1.22E-02	5.05E-03	5.00E-01	-	1.62E-02
61	C133 /II/22/23	2.43E-04	1.44E-02	-	5.45E-04	1.22E-03	7.20E-02	-	2.73E-03	2.43E-03	1.44E-01	-	5.45E-03	3.65E-03	2.16E-01	-	8.18E-03	4.87E-03	2.88E-01	-	1.09E-02
62	C134 /II/22/23	3.14E-04	2.91E-02	-	6.15E-04	1.57E-03	1.45E-01	-	3.07E-03	3.14E-03	2.91E-01	-	6.15E-03	4.72E-03	4.36E-01	-	9.22E-03	6.29E-03	5.82E-01	-	1.23E-02
63	C135 /II/22/23	2.42E-04	1.24E-02	-	4.78E-04	1.21E-03	6.21E-02	-	2.39E-03	2.42E-03	1.24E-01	-	4.78E-03	3.63E-03	1.86E-01	-	7.16E-03	4.84E-03	2.48E-01	-	9.55E-03
64	C136 /II/22/23	2.52E-04	7.72E-03	2.53E-04	8.57E-04	1.26E-03	3.86E-02	1.26E-03	4.28E-03	2.52E-03	7.72E-02	1.26E-03	8.57E-03	3.78E-03	1.16E-01	6.32E-02	1.29E-02	5.03E-03	1.54E-01	5.06E-03	1.71E-02
65	C137 /II/22/23	2.47E-04	2.68E-03	1.94E-04	4.06E-04	1.24E-03	1.34E-02	9.72E-04	2.03E-03	2.47E-03	2.68E-02	9.72E-04	4.06E-03	3.71E-03	4.02E-02	4.86E-02	6.09E-03	4.94E-03	5.36E-02	3.89E-03	8.11E-03
66	C138 /II/22/23	2.68E-04	3.26E-03	2.95E-04	8.56E-04	1.34E-03	1.63E-02	1.48E-03	4.28E-03	2.68E-03	3.26E-02	1.48E-03	8.56E-03	4.02E-03	4.89E-02	7.38E-02	1.28E-02	5.37E-03	6.52E-02	5.90E-03	1.71E-02
67	C139 /II/22/23	2.98E-04	2.00E-03	-	5.03E-04	1.49E-03	9.99E-03	-	2.51E-03	2.98E-03	2.00E-02	-	5.03E-03	4.48E-03	3.00E-02	-	7.54E-03	5.97E-03	4.00E-02	-	1.01E-02
68	C140 /II/22/23	2.17E-04	1.55E-03	-	4.46E-04	1.08E-03	7.76E-03	-	2.23E-03	2.17E-03	1.55E-02	-	4.46E-03	3.25E-03	2.33E-02	-	6.68E-03	4.34E-03	3.10E-02	-	8.91E-03
69	C141 /II/22/23	2.30E-04	1.68E-03	-	4.36E-04	1.15E-03	8.41E-03	-	2.18E-03	2.30E-03	1.68E-02	-	4.36E-03	3.45E-03	2.52E-02	-	6.54E-03	4.60E-03	3.36E-02	-	8.72E-03
70	C142 /II/22/23	2.33E-04	3.96E-03	-	5.44E-04	1.16E-03	1.98E-02	-	2.72E-03	2.33E-03	3.96E-02	-	5.44E-03	3.49E-03	5.93E-02	-	8.16E-03	4.66E-03	7.91E-02	-	1.09E-02
71	C143 /II/22/23	3.05E-04	-	-	6.83E-04	1.52E-03	-	-	3.41E-03	3.05E-03	-	-	6.83E-03	4.57E-03	-	-	1.02E-02	6.10E-03	-	-	1.37E-02
72	C144 /II/22/23	2.48E-04	5.05E-03	-	5.41E-04	1.24E-03	2.53E-02	-	2.70E-03	2.48E-03	5.05E-02	-	5.41E-03	3.72E-03	7.58E-02	-	8.11E-03	4.96E-03	1.01E-01	-	1.08E-02
73	C145 /II/22/23	2.97E-04	1.41E-03	-	8.91E-04	1.48E-03	7.03E-03	-	4.45E-03	2.97E-03	1.41E-02	-	8.91E-03	4.45E-03	2.11E-02	-	1.34E-02	5.93E-03	2.81E-02	-	1.78E-02
74	C146 /II/22/23	3.75E-04	3.05E-03	7.04E-05	7.66E-04	1.88E-03	1.52E-02	3.52E-04	3.83E-03	3.75E-03	3.05E-02	3.52E-04	7.66E-03	5.63E-03	4.57E-02	1.76E-02	1.15E-02	7.51E-03	6.10E-02	1.41E-03	1.53E-02

75	C147 /II/22/23	3.04E-04	9.99E-04	-	5.15E-04	1.52E-03	5.00E-03	-	2.58E-03	3.04E-03	9.99E-03	-	5.15E-03	4.56E-03	1.50E-02	-	7.73E-03	6.08E-03	2.00E-02	-	1.03E-02
76	C148 /II/22/23	3.18E-04	8.57E-04	-	6.34E-04	1.59E-03	4.28E-03	-	3.17E-03	3.18E-03	8.57E-03	-	6.34E-03	4.76E-03	1.29E-02	-	9.50E-03	6.35E-03	1.71E-02	-	1.27E-02
77	C149 /II/22/23	3.03E-04	-	-	7.37E-04	1.52E-03	-	-	3.68E-03	3.03E-03	-	-	7.37E-03	4.55E-03	-	-	1.10E-02	6.07E-03	-	-	1.47E-02
78	C15 /II/22/23	2.51E-04	2.16E-03	-	7.43E-04	1.25E-03	1.08E-02	-	3.72E-03	2.51E-03	2.16E-02	-	7.43E-03	3.76E-03	3.24E-02	-	1.12E-02	5.02E-03	4.32E-02	-	1.49E-02
79	C86 /II/21/22	2.95E-04	7.71E-05	-	7.80E-04	1.48E-03	3.86E-04	-	3.90E-03	2.95E-03	7.71E-04	-	7.80E-03	4.43E-03	1.16E-03	-	1.17E-02	5.90E-03	1.54E-03	-	1.56E-02
80	C87 /II/21/22	2.40E-04	-	-	5.55E-04	1.20E-03	-	-	2.78E-03	2.40E-03	-	-	5.55E-03	3.61E-03	-	-	8.33E-03	4.81E-03	-	-	1.11E-02
81	C88 /II/21/22	2.53E-04	4.81E-05	-	7.67E-04	1.26E-03	2.40E-04	-	3.83E-03	2.53E-03	4.81E-04	-	7.67E-03	3.79E-03	7.21E-04	-	1.15E-02	5.06E-03	9.62E-04	-	1.53E-02
82	C89 /II/21/22	2.48E-04	5.26E-05	7.39E-05	4.01E-04	1.24E-03	2.63E-04	3.70E-04	2.01E-03	2.48E-03	5.26E-04	3.70E-04	4.01E-03	3.72E-03	7.90E-04	1.85E-02	6.02E-03	4.96E-03	1.05E-03	1.48E-03	8.03E-03
83	C90 /II/21/22	2.45E-04	5.24E-05	1.09E-04	8.05E-04	1.23E-03	2.62E-04	5.46E-04	4.02E-03	2.45E-03	5.24E-04	5.46E-04	8.05E-03	3.68E-03	7.87E-04	2.73E-02	1.21E-02	4.91E-03	1.05E-03	2.18E-03	1.61E-02
84	C91 /II/21/22	2.53E-04	-	-	7.37E-04	1.27E-03	-	-	3.69E-03	2.53E-03	-	-	7.37E-03	3.80E-03	-	-	1.11E-02	5.06E-03	-	-	1.47E-02
85	C92 /II/21/22	2.21E-04	6.17E-05	-	7.53E-04	1.10E-03	3.08E-04	-	3.76E-03	2.21E-03	6.17E-04	-	7.53E-03	3.31E-03	9.25E-04	-	1.13E-02	4.42E-03	1.23E-03	-	1.51E-02
86	C93 /II/21/22	2.14E-04	3.89E-05	-	4.07E-04	1.07E-03	1.94E-04	-	2.04E-03	2.14E-03	3.89E-04	-	4.07E-03	3.21E-03	5.83E-04	-	6.11E-03	4.28E-03	7.77E-04	-	8.15E-03
87	C94 /II/21/22	1.97E-04	4.52E-05	7.46E-05	3.81E-04	9.83E-04	2.26E-04	3.73E-04	1.91E-03	1.97E-03	4.52E-04	3.73E-04	3.81E-03	2.95E-03	6.79E-04	1.87E-02	5.72E-03	3.93E-03	9.05E-04	1.49E-03	7.63E-03
88	C95 /II/21/22	2.16E-04	4.07E-05	-	3.25E-04	1.08E-03	2.04E-04	-	1.63E-03	2.16E-03	4.07E-04	-	3.25E-03	3.24E-03	6.11E-04	-	4.88E-03	4.32E-03	8.15E-04	-	6.51E-03
89	C96 /II/21/22	2.30E-04	-	-	4.88E-04	1.15E-03	-	-	2.44E-03	2.30E-03	-	-	4.88E-03	3.45E-03	-	-	7.31E-03	4.60E-03	-	-	9.75E-03
90	C97 /II/21/22	2.44E-04	-	7.85E-05	5.27E-04	1.22E-03	-	3.92E-04	2.64E-03	2.44E-03	-	3.92E-04	5.27E-03	3.66E-03	-	1.96E-02	7.91E-03	4.88E-03	-	1.57E-03	1.05E-02
91	C98 /II/21/22	4.71E-04	-	-	5.04E-04	2.36E-03	-	-	2.52E-03	4.71E-03	-	-	5.04E-03	7.07E-03	-	-	7.56E-03	9.42E-03	-	-	1.01E-02
92	C99 /II/21/22	3.57E-04	5.31E-05	7.33E-05	8.21E-04	1.78E-03	2.65E-04	3.67E-04	4.10E-03	3.57E-03	5.31E-04	3.67E-04	8.21E-03	5.35E-03	7.96E-04	1.83E-02	1.23E-02	7.13E-03	1.06E-03	1.47E-03	1.64E-02
93	C101 /II/21/22	2.83E-04	5.85E-05	8.23E-05	8.33E-04	1.42E-03	2.93E-04	4.12E-04	4.17E-03	2.83E-03	5.85E-04	4.12E-04	8.33E-03	4.25E-03	8.78E-04	2.06E-02	1.25E-02	5.67E-03	1.17E-03	1.65E-03	1.67E-02

94	C16 /II/21/22	2.07E-04	-	9.42E-05	6.11E-04	1.04E-03	-	4.71E-04	3.06E-03	2.07E-03	-	4.71E-04	6.11E-03	3.11E-03	-	2.36E-02	9.17E-03	4.14E-03	-	1.88E-03	1.22E-02
95	C17 /II/21/22	2.00E-04	8.20E-05	-	1.07E-03	1.00E-03	4.10E-04	-	5.34E-03	2.00E-03	8.20E-04	-	1.07E-02	3.00E-03	1.23E-03	-	1.60E-02	4.00E-03	1.64E-03	-	2.13E-02
96	C18 /II/21/22	1.99E-04	3.29E-05	6.51E-04	7.95E-04	9.96E-04	1.65E-04	3.25E-03	3.98E-03	1.99E-03	3.29E-04	3.25E-03	7.95E-03	2.99E-03	4.94E-04	1.63E-01	1.19E-02	3.98E-03	6.58E-04	1.30E-02	1.59E-02
97	C21 /II/21/22	1.24E-04	-	1.23E-04	1.01E-03	6.19E-04	-	6.16E-04	5.06E-03	1.24E-03	-	6.16E-04	1.01E-02	1.86E-03	-	3.08E-02	1.52E-02	2.48E-03	-	2.46E-03	2.02E-02
98	C39 /II/21/22	2.47E-04	-	1.34E-04	7.09E-04	1.24E-03	-	6.71E-04	3.54E-03	2.47E-03	-	6.71E-04	7.09E-03	3.71E-03	-	3.35E-02	1.06E-02	4.95E-03	-	2.68E-03	1.42E-02
99	C40 /II/21/22	2.18E-04	-	1.21E-04	8.93E-04	1.09E-03	-	6.05E-04	4.47E-03	2.18E-03	-	6.05E-04	8.93E-03	3.27E-03	-	3.03E-02	1.34E-02	4.36E-03	-	2.42E-03	1.79E-02
100	C41 /II/21/22	2.93E-04	6.96E-05	1.08E-04	7.18E-04	1.47E-03	3.48E-04	5.40E-04	3.59E-03	2.93E-03	6.96E-04	5.40E-04	7.18E-03	4.40E-03	1.04E-03	2.70E-02	1.08E-02	5.87E-03	1.39E-03	2.16E-03	1.44E-02
101	C43 /II/21/22	4.86E-04	3.52E-05	2.70E-04	9.20E-04	2.43E-03	1.76E-04	1.35E-03	4.60E-03	4.86E-03	3.52E-04	1.35E-03	9.20E-03	7.29E-03	5.28E-04	6.76E-02	1.38E-02	9.72E-03	7.05E-04	5.41E-03	1.84E-02
102	C44 /II/21/22	1.95E-03	-	1.05E-04	9.78E-04	9.76E-03	-	5.25E-04	4.89E-03	1.95E-02	-	5.25E-04	9.78E-03	2.93E-02	-	2.62E-02	1.47E-02	3.90E-02	-	2.10E-03	1.96E-02
103	C46 /II/21/22	3.09E-04	-	1.78E-04	8.54E-04	1.54E-03	-	8.89E-04	4.27E-03	3.09E-03	-	8.89E-04	8.54E-03	4.63E-03	-	4.44E-02	1.28E-02	6.17E-03	-	3.55E-03	1.71E-02
104	C314 /II/17/18	-	2.77E-02	-	7.22E-03	-	1.38E-01	-	3.61E-02	-	2.77E-01	-	7.22E-02	-	4.15E-01	-	1.08E-01	-	5.54E-01	-	1.44E-01
105	C315 /II/17/18	-	3.02E-03	-	4.22E-03	-	1.51E-02	-	2.11E-02	-	3.02E-02	-	4.22E-02	-	4.54E-02	-	6.33E-02	-	6.05E-02	-	8.45E-02
106	C319 /II/17/18	-	1.70E-03	-	4.72E-04	-	8.51E-03	-	2.36E-03	-	1.70E-02	-	4.72E-03	-	2.55E-02	-	7.08E-03	-	3.41E-02	-	9.44E-03
107	C320 /II/17/18	-	9.69E-04	-	2.41E-03	-	4.85E-03	-	1.21E-02	-	9.69E-03	-	2.41E-02	-	1.45E-02	-	3.62E-02	-	1.94E-02	-	4.82E-02
108	C321 /II/17/18	-	3.32E-03	-	2.42E-03	-	1.66E-02	-	1.21E-02	-	3.32E-02	-	2.42E-02	-	4.98E-02	-	3.63E-02	-	6.64E-02	-	4.84E-02
109	C322 /II/17/18	-	3.52E-03	-	9.10E-04	-	1.76E-02	-	4.55E-03	-	3.52E-02	-	9.10E-03	-	5.28E-02	-	1.37E-02	-	7.04E-02	-	1.82E-02
110	C323 /II/17/18	8.42E-04	-	2.18E-04	2.44E-04	4.21E-03	-	1.09E-03	1.22E-03	8.42E-03	-	1.09E-03	2.44E-03	1.26E-02	-	5.46E-02	3.67E-03	1.68E-02	-	4.37E-03	4.89E-03
111	C324 /II/17/18	-	2.88E-03	-	2.52E-03	-	1.44E-02	-	1.26E-02	-	2.88E-02	-	2.52E-02	-	4.33E-02	-	3.78E-02	-	5.77E-02	-	5.04E-02

112	C35 /I/23/24	1.85E-04	3.90E-04	-	8.26E-04	9.25E-04	1.95E-03	-	4.13E-03	1.85E-03	3.90E-03	-	8.26E-03	2.78E-03	5.86E-03	-	1.24E-02	3.70E-03	7.81E-03	-	1.65E-02
113	C168 /I/22/23	2.08E-04	-	-	3.91E-04	1.04E-03	-	-	1.96E-03	2.08E-03	-	-	3.91E-03	3.11E-03	-	-	5.87E-03	4.15E-03	-	-	7.83E-03
114	C175 /I/22/23	2.06E-04	5.89E-05	-	5.65E-04	1.03E-03	2.94E-04	-	2.82E-03	2.06E-03	5.89E-04	-	5.65E-03	3.09E-03	8.83E-04	-	8.47E-03	4.12E-03	1.18E-03	-	1.13E-02
115	C100 /II/21/22	1.32E-04	3.89E-05	-	1.26E-03	6.61E-04	1.95E-04	-	6.31E-03	1.32E-03	3.89E-04	-	1.26E-02	1.98E-03	5.84E-04	-	1.89E-02	2.64E-03	7.78E-04	-	2.53E-02
116	C20 /II/21/22	1.90E-04	4.52E-05	1.74E-04	9.15E-04	9.48E-04	2.26E-04	8.69E-04	4.58E-03	1.90E-03	4.52E-04	8.69E-04	9.15E-03	2.84E-03	6.78E-04	4.34E-02	1.37E-02	3.79E-03	9.03E-04	3.47E-03	1.83E-02
117	C316 /II/17/18	-	1.78E-03	-	8.09E-04	-	8.91E-03	-	4.05E-03	-	1.78E-02	-	8.09E-03	-	2.67E-02	-	1.21E-02	-	3.57E-02	-	1.62E-02
118	C317 /II/17/18	-	2.37E-03	-	5.23E-04	-	1.18E-02	-	2.61E-03	-	2.37E-02	-	5.23E-03	-	3.55E-02	-	7.84E-03	-	4.73E-02	-	1.05E-02
119	C326 /II/17/18	7.08E-05	1.59E-03	2.31E-04	3.41E-03	3.54E-04	7.94E-03	1.16E-03	1.71E-02	7.08E-04	1.59E-02	1.16E-03	3.41E-02	1.06E-03	2.38E-02	5.78E-02	5.12E-02	1.42E-03	3.18E-02	4.62E-03	6.83E-02

Table S7. Lifetime Average Daily Dose (LADD) [mg/kg] for inhalation exposure to lead, nickel, and cadmium contained in the cigarettes tested.

No.	Sample code	1 cigarette per day			5 cigarettes per day			10 cigarettes per day			15 cigarettes per day			20 cigarettes per day		
		Pb	Ni	Cd	Pb	Ni	Cd	Pb	Ni	Cd	Pb	Ni	Cd	Pb	Ni	Cd
1	C20 /I/23/24	7.15E-05	-	3.04E-05	3.57E-04	-	1.52E-04	7.15E-04	-	3.04E-04	1.07E-03	-	4.57E-04	1.43E-03	-	6.09E-04
2	C21 /I/23/24	2.40E-05	-	2.31E-05	1.20E-04	-	1.16E-04	2.40E-04	-	2.31E-04	3.60E-04	-	3.47E-04	4.80E-04	-	4.63E-04
3	C22 /I/23/24	5.69E-05	-	2.88E-05	2.85E-04	-	1.44E-04	5.69E-04	-	2.88E-04	8.54E-04	-	4.32E-04	1.14E-03	-	5.76E-04
4	C23 /I/23/24	5.20E-05	-	3.39E-05	2.60E-04	-	1.69E-04	5.20E-04	-	3.39E-04	7.81E-04	-	5.08E-04	1.04E-03	-	6.78E-04
5	C24 /I/23/24	6.05E-06	-	2.72E-05	3.02E-05	-	1.36E-04	6.05E-05	-	2.72E-04	9.07E-05	-	4.07E-04	1.21E-04	-	5.43E-04
6	C25 /I/23/24	4.75E-05	-	2.54E-05	2.38E-04	-	1.27E-04	4.75E-04	-	2.54E-04	7.13E-04	-	3.80E-04	9.50E-04	-	5.07E-04
7	C26 /I/23/24	4.83E-05	-	5.33E-05	2.42E-04	-	2.66E-04	4.83E-04	-	5.33E-04	7.25E-04	-	7.99E-04	9.67E-04	-	1.07E-03
8	C27 /I/23/24	2.82E-05	-	4.07E-05	1.41E-04	-	2.04E-04	2.82E-04	-	4.07E-04	4.23E-04	-	6.11E-04	5.63E-04	-	8.15E-04
9	C28 /I/23/24	3.91E-05	-	2.73E-05	1.96E-04	-	1.37E-04	3.91E-04	-	2.73E-04	5.87E-04	-	4.10E-04	7.82E-04	-	5.47E-04
10	C29 /I/23/24	2.34E-05	-	4.00E-05	1.17E-04	-	2.00E-04	2.34E-04	-	4.00E-04	3.51E-04	-	6.00E-04	4.68E-04	-	8.01E-04
11	C30 /I/23/24	-	-	3.43E-05	-	-	1.72E-04	-	-	3.43E-04	-	-	5.15E-04	-	-	6.86E-04
12	C31 /I/23/24	5.17E-05	-	2.61E-05	2.59E-04	-	1.30E-04	5.17E-04	-	2.61E-04	7.76E-04	-	3.91E-04	1.03E-03	-	5.21E-04
13	C32 /I/23/24	5.20E-05	-	3.80E-05	2.60E-04	-	1.90E-04	5.20E-04	-	3.80E-04	7.80E-04	-	5.71E-04	1.04E-03	-	7.61E-04
14	C33 /I/23/24	4.21E-05	-	2.94E-05	2.10E-04	-	1.47E-04	4.21E-04	-	2.94E-04	6.31E-04	-	4.41E-04	8.42E-04	-	5.88E-04
15	C34 /I/23/24	2.94E-05	-	3.32E-05	1.47E-04	-	1.66E-04	2.94E-04	-	3.32E-04	4.41E-04	-	4.98E-04	5.87E-04	-	6.64E-04
16	C36 /I/23/24	5.28E-05	-	3.57E-05	2.64E-04	-	1.78E-04	5.28E-04	-	3.57E-04	7.92E-04	-	5.35E-04	1.06E-03	-	7.14E-04
17	C37 /I/23/24	1.03E-04	-	4.04E-05	5.17E-04	-	2.02E-04	1.03E-03	-	4.04E-04	1.55E-03	-	6.07E-04	2.07E-03	-	8.09E-04

18	C38 /I/23/24	6.59E-05	-	3.48E-05	3.30E-04	-	1.74E-04	6.59E-04	-	3.48E-04	9.89E-04	-	5.22E-04	1.32E-03	-	6.95E-04
19	C39 /I/23/24	3.72E-05	-	2.39E-05	1.86E-04	-	1.19E-04	3.72E-04	-	2.39E-04	5.58E-04	-	3.58E-04	7.44E-04	-	4.78E-04
20	C128 /I/22/23	7.26E-06	8.68E-05	2.69E-05	3.63E-05	4.34E-04	1.34E-04	7.26E-05	8.68E-04	2.69E-04	1.09E-04	1.30E-03	4.03E-04	1.45E-04	1.74E-03	5.38E-04
21	C129 /I/22/23	-	8.58E-05	1.69E-05	-	4.29E-04	8.47E-05	-	8.58E-04	1.69E-04	-	1.29E-03	2.54E-04	-	1.72E-03	3.39E-04
22	C130 /I/22/23	1.27E-05	8.46E-05	1.85E-05	6.36E-05	4.23E-04	9.25E-05	1.27E-04	8.46E-04	1.85E-04	1.91E-04	1.27E-03	2.78E-04	2.54E-04	1.69E-03	3.70E-04
23	C131 /I/22/23	5.83E-06	1.69E-04	1.40E-05	2.91E-05	8.45E-04	7.02E-05	5.83E-05	1.69E-03	1.40E-04	8.74E-05	2.54E-03	2.11E-04	1.17E-04	3.38E-03	2.81E-04
24	C132 /I/22/23	1.40E-05	3.52E-04	1.37E-04	7.00E-05	1.76E-03	6.83E-04	1.40E-04	3.52E-03	1.37E-03	2.10E-04	5.27E-03	2.05E-03	2.80E-04	7.03E-03	2.73E-03
25	C133 /I/22/23	-	2.95E-04	1.70E-05	-	1.48E-03	8.48E-05	-	2.95E-03	1.70E-04	-	4.43E-03	2.54E-04	-	5.90E-03	3.39E-04
26	C134 /I/22/23	-	-	3.96E-05	-	-	1.98E-04	-	-	3.96E-04	-	-	5.93E-04	-	-	7.91E-04
27	C135 /I/22/23	-	2.19E-04	2.34E-05	-	1.09E-03	1.17E-04	-	2.19E-03	2.34E-04	-	3.28E-03	3.50E-04	-	4.37E-03	4.67E-04
28	C136 /I/22/23	-	3.89E-04	3.99E-05	-	1.94E-03	2.00E-04	-	3.89E-03	3.99E-04	-	5.83E-03	5.99E-04	-	7.77E-03	7.98E-04
29	C137 /I/22/23	9.32E-06	4.31E-04	1.61E-05	4.66E-05	2.15E-03	8.03E-05	9.32E-05	4.31E-03	1.61E-04	1.40E-04	6.46E-03	2.41E-04	1.86E-04	8.62E-03	3.21E-04
30	C138 /I/22/23	-	2.22E-04	1.99E-05	-	1.11E-03	9.94E-05	-	2.22E-03	1.99E-04	-	3.33E-03	2.98E-04	-	4.45E-03	3.98E-04
31	C169 /I/22/23	1.27E-05	-	5.07E-05	6.33E-05	-	2.54E-04	1.27E-04	-	5.07E-04	1.90E-04	-	7.61E-04	2.53E-04	-	1.01E-03
32	C170 /I/22/23	-	9.16E-05	2.10E-05	-	4.58E-04	1.05E-04	-	9.16E-04	2.10E-04	-	1.37E-03	3.15E-04	-	1.83E-03	4.19E-04
33	C171 /I/22/23	-	7.29E-05	2.58E-05	-	3.65E-04	1.29E-04	-	7.29E-04	2.58E-04	-	1.09E-03	3.87E-04	-	1.46E-03	5.16E-04
34	C172 /I/22/23	-	-	2.07E-05	-	-	1.04E-04	-	-	2.07E-04	-		3.11E-04	-		4.14E-04
35	C173 /I/22/23	-	8.93E-05	2.37E-05	-	4.47E-04	1.18E-04	-	8.93E-04	2.37E-04	-	1.34E-03	3.55E-04	-	1.79E-03	4.73E-04
36	C174 /I/22/23	-	9.22E-05	3.39E-05	-	4.61E-04	1.70E-04	-	9.22E-04	3.39E-04	-	1.38E-03	5.09E-04	-	1.84E-03	6.79E-04

37	C176 /I/22/23	8.26E-06	8.94E-05	3.08E-05	4.13E-05	4.47E-04	1.54E-04	8.26E-05	8.94E-04	3.08E-04	1.24E-04	1.34E-03	4.63E-04	1.65E-04	1.79E-03	6.17E-04
38	C177 /I/22/23	-	6.29E-05	3.10E-05		3.15E-04	1.55E-04	-	6.29E-04	3.10E-04	-	9.44E-04	4.65E-04	-	1.26E-03	6.20E-04
39	C178 /I/22/23	1.10E-05	7.16E-05	3.83E-05	5.51E-05	3.58E-04	1.92E-04	1.10E-04	7.16E-04	3.83E-04	1.65E-04	1.07E-03	5.75E-04	2.20E-04	1.43E-03	7.66E-04
40	C179 /I/22/23	-	-	2.76E-05	-	-	1.38E-04	-	-	2.76E-04	-	-	4.14E-04	-	-	5.52E-04
41	C180 /I/22/23	-	1.09E-04	2.64E-05	-	5.44E-04	1.32E-04	-	1.09E-03	2.64E-04	-	1.63E-03	3.96E-04	-	2.18E-03	5.28E-04
42	C181 /I/22/23	6.11E-06	9.35E-05	1.76E-05	3.05E-05	4.67E-04	8.80E-05	6.11E-05	9.35E-04	1.76E-04	9.16E-05	1.40E-03	2.64E-04	1.22E-04	1.87E-03	3.52E-04
43	C182 /I/22/23	1.06E-05	8.06E-05	2.27E-05	5.31E-05	4.03E-04	1.14E-04	1.06E-04	8.06E-04	2.27E-04	1.59E-04	1.21E-03	3.41E-04	2.12E-04	1.61E-03	4.55E-04
44	C183 /I/22/23	1.14E-05	-	2.64E-05	5.68E-05	-	1.32E-04	1.14E-04	-	2.64E-04	1.70E-04	-	3.96E-04	2.27E-04	-	5.28E-04
45	C184 /I/22/23	1.57E-05	-	1.49E-05	7.86E-05	-	7.45E-05	1.57E-04	-	1.49E-04	2.36E-04	-	2.23E-04	3.14E-04	-	2.98E-04
46	C185 /I/22/23	5.98E-06	-	1.14E-05	2.99E-05	-	5.71E-05	5.98E-05	-	1.14E-04	8.97E-05	-	1.71E-04	1.20E-04	-	2.28E-04
47	C186 /I/22/23	-	8.17E-05	1.52E-05	-	4.08E-04	7.61E-05	-	8.17E-04	1.52E-04	-	1.23E-03	2.28E-04	-	1.63E-03	3.04E-04
48	C187 /I/22/23	-	7.13E-05	2.01E-05	-	3.57E-04	1.01E-04	-	7.13E-04	2.01E-04	-	1.07E-03	3.02E-04	-	1.43E-03	4.03E-04
49	C188 /I/22/23	2.29E-05	9.49E-05	1.29E-05	1.15E-04	4.74E-04	6.46E-05	2.29E-04	9.49E-04	1.29E-04	3.44E-04	1.42E-03	1.94E-04	4.58E-04	1.90E-03	2.59E-04
50	C64 /II/22/23	-	8.47E-05	3.08E-05	-	4.23E-04	1.54E-04	-	8.47E-04	3.08E-04	-	1.27E-03	4.62E-04	-	1.69E-03	6.16E-04
51	C65 /II/22/23	-	3.86E-04	2.79E-05	-	1.93E-03	1.39E-04	-	3.86E-03	2.79E-04	-	5.79E-03	4.18E-04	-	7.73E-03	5.58E-04
52	C66 /II/22/23	-	-	3.17E-05	-	-	1.59E-04	-	-	3.17E-04	-	-	4.76E-04	-	-	6.35E-04
53	C67 /II/22/23	-	1.04E-04	2.73E-05	-	5.19E-04	1.36E-04	-	1.04E-03	2.73E-04	-	1.56E-03	4.09E-04	-	2.08E-03	5.46E-04
54	C68 /II/22/23	-	-	2.33E-05	-	-	1.17E-04	-	-	2.33E-04	-	-	3.50E-04	-	-	4.66E-04
55	C69 /II/22/23	-	1.16E-04	2.25E-05	-	5.80E-04	1.13E-04	-	1.16E-03	2.25E-04	-	1.74E-03	3.38E-04	-	2.32E-03	4.50E-04

56	C70 /II/22/23	-	-	2.59E-05	-	-	1.29E-04	-	-	2.59E-04	-	-	3.88E-04	-	-	5.18E-04
57	C128 /II/22/23	6.01E-02	-	2.33E-05	3.01E-01	-	1.17E-04	6.01E-01	-	2.33E-04	9.02E-01	-	3.50E-04	1.20E+00	-	4.66E-04
58	C129 /II/22/23	3.11E-02	-	2.57E-05	1.56E-01	-	1.28E-04	3.11E-01	-	2.57E-04	4.67E-01	-	3.85E-04	6.23E-01	-	5.13E-04
59	C130 /II/22/23	1.53E-02	-	2.38E-05	7.67E-02	-	1.19E-04	1.53E-01	-	2.38E-04	2.30E-01	-	3.57E-04	3.07E-01	-	4.76E-04
60	C131 /II/22/23	4.37E-03	-	3.54E-05	2.19E-02	-	1.77E-04	4.37E-02	-	3.54E-04	6.56E-02	-	5.31E-04	8.74E-02	-	7.08E-04
61	C133 /II/22/23	2.51E-03	-	2.38E-05	1.26E-02	-	1.19E-04	2.51E-02	-	2.38E-04	3.77E-02	-	3.57E-04	5.03E-02	-	4.76E-04
62	C134 /II/22/23	5.08E-03	-	2.68E-05	2.54E-02	-	1.34E-04	5.08E-02	-	2.68E-04	7.62E-02	-	4.03E-04	1.02E-01	-	5.37E-04
63	C135 /II/22/23	2.17E-03	-	2.09E-05	1.08E-02	-	1.04E-04	2.17E-02	-	2.09E-04	3.25E-02	-	3.13E-04	4.34E-02	-	4.17E-04
64	C136 /II/22/23	1.35E-03	2.21E-04	3.74E-05	6.74E-03	1.10E-03	1.87E-04	1.35E-02	2.21E-03	3.74E-04	2.02E-02	3.31E-03	5.61E-04	2.70E-02	4.42E-03	7.48E-04
65	C137 /II/22/23	4.68E-04	1.70E-04	1.77E-05	2.34E-03	8.49E-04	8.86E-05	4.68E-03	1.70E-03	1.77E-04	7.02E-03	2.55E-03	2.66E-04	9.36E-03	3.40E-03	3.54E-04
66	C138 /II/22/23	5.69E-04	2.58E-04	3.74E-05	2.85E-03	1.29E-03	1.87E-04	5.69E-03	2.58E-03	3.74E-04	8.54E-03	3.87E-03	5.61E-04	1.14E-02	5.16E-03	7.48E-04
67	C139 /II/22/23	3.49E-04	-	2.19E-05	1.75E-03	-	1.10E-04	3.49E-03	-	2.19E-04	5.24E-03	-	3.29E-04	6.98E-03	-	4.39E-04
68	C140 /II/22/23	2.71E-04	-	1.95E-05	1.35E-03	-	9.73E-05	2.71E-03	-	1.95E-04	4.06E-03	-	2.92E-04	5.42E-03	-	3.89E-04
69	C141 /II/22/23	2.94E-04	-	1.90E-05	1.47E-03	-	9.52E-05	2.94E-03	-	1.90E-04	4.41E-03	-	2.86E-04	5.88E-03	-	3.81E-04
70	C142 /II/22/23	6.91E-04	-	2.38E-05	3.46E-03	-	1.19E-04	6.91E-03	-	2.38E-04	1.04E-02	-	3.57E-04	1.38E-02	-	4.75E-04
71	C143 /II/22/23	-	-	2.98E-05	-	-	1.49E-04	-	-	2.98E-04	-	-	4.47E-04	-	-	5.96E-04
72	C144 /II/22/23	8.83E-04	-	2.36E-05	4.41E-03	-	1.18E-04	8.83E-03	-	2.36E-04	1.32E-02	-	3.54E-04	1.77E-02	-	4.72E-04
73	C145 /II/22/23	2.45E-04	-	3.89E-05	1.23E-03	-	1.94E-04	2.45E-03	-	3.89E-04	3.68E-03	-	5.83E-04	4.91E-03	-	7.78E-04
74	C146 /II/22/23	5.32E-04	6.15E-05	3.34E-05	2.66E-03	3.07E-04	1.67E-04	5.32E-03	6.15E-04	3.34E-04	7.99E-03	9.22E-04	5.02E-04	1.06E-02	1.23E-03	6.69E-04

75	C147 /II/22/23	1.75E-04	-	2.25E-05	8.73E-04	-	1.13E-04	1.75E-03	-	2.25E-04	2.62E-03	-	3.38E-04	3.49E-03	-	4.50E-04
76	C148 /II/22/23	1.50E-04	-	2.77E-05	7.48E-04	-	1.38E-04	1.50E-03	-	2.77E-04	2.24E-03	-	4.15E-04	2.99E-03	-	5.53E-04
77	C149 /II/22/23	-	-	3.22E-05	-	-	1.61E-04		-	3.22E-04		-	4.83E-04		-	6.43E-04
78	C15 /II/22/23	3.77E-04	-	3.25E-05	1.88E-03	-	1.62E-04	3.77E-03	-	3.25E-04	5.65E-03	-	4.87E-04	7.54E-03	-	6.49E-04
79	C86 /II/21/22	1.35E-05	-	3.41E-05	6.74E-05	-	1.70E-04	1.35E-04	-	3.41E-04	2.02E-04	-	5.11E-04	2.69E-04	-	6.82E-04
80	C87 /II/21/22	-	-	2.42E-05	-	-	1.21E-04	-	-	2.42E-04	-	-	3.64E-04	-	-	4.85E-04
81	C88 /II/21/22	8.40E-06	-	3.35E-05	4.20E-05	-	1.67E-04	8.40E-05	-	3.35E-04	1.26E-04	-	5.02E-04	1.68E-04	-	6.70E-04
82	C89 /II/21/22	9.20E-06	6.46E-05	1.75E-05	4.60E-05	3.23E-04	8.76E-05	9.20E-05	6.46E-04	1.75E-04	1.38E-04	9.69E-04	2.63E-04	1.84E-04	1.29E-03	3.51E-04
83	C90 /II/21/22	9.16E-06	9.53E-05	3.51E-05	4.58E-05	4.77E-04	1.76E-04	9.16E-05	9.53E-04	3.51E-04	1.37E-04	1.43E-03	5.27E-04	1.83E-04	1.91E-03	7.03E-04
84	C91 /II/21/22	-	-	3.22E-05	-	-	1.61E-04	-	-	3.22E-04	-	-	4.83E-04	-	-	6.44E-04
85	C92 /II/21/22	1.08E-05	-	3.29E-05	5.39E-05	-	1.64E-04	1.08E-04	-	3.29E-04	1.62E-04	-	4.93E-04	2.15E-04	-	6.57E-04
86	C93 /II/21/22	6.79E-06	-	1.78E-05	3.39E-05	-	8.89E-05	6.79E-05	-	1.78E-04	1.02E-04	-	2.67E-04	1.36E-04	-	3.56E-04
87	C94 /II/21/22	7.90E-06	6.52E-05	1.67E-05	3.95E-05	3.26E-04	8.33E-05	7.90E-05	6.52E-04	1.67E-04	1.19E-04	9.78E-04	2.50E-04	1.58E-04	1.30E-03	3.33E-04
88	C95 /II/21/22	7.12E-06		1.42E-05	3.56E-05	-	7.10E-05	7.12E-05	-	1.42E-04	1.07E-04	-	2.13E-04	1.42E-04	-	2.84E-04
89	C96 /II/21/22	-		2.13E-05	-	-	1.06E-04	-	-	2.13E-04	-	-	3.19E-04	-	-	4.26E-04
90	C97 /II/21/22	-	6.86E-05	2.30E-05	-	3.43E-04	1.15E-04	-	6.86E-04	2.30E-04	-	1.03E-03	3.45E-04	-	1.37E-03	4.60E-04
91	C98 /II/21/22	-	-	2.20E-05	-	-	1.10E-04	-	-	2.20E-04	-	-	3.30E-04	-	-	4.40E-04
92	C99 /II/21/22	9.27E-06	6.41E-05	3.58E-05	4.64E-05	3.20E-04	1.79E-04	9.27E-05	6.41E-04	3.58E-04	1.39E-04	9.61E-04	5.38E-04	1.85E-04	1.28E-03	7.17E-04
93	C101 /II/21/22	1.02E-05	7.19E-05	3.64E-05	5.11E-05	3.60E-04	1.82E-04	1.02E-04	7.19E-04	3.64E-04	1.53E-04	1.08E-03	5.46E-04	2.04E-04	1.44E-03	7.28E-04

94	C16 /II/21/22	-	8.23E-05	2.67E-05	-	4.11E-04	1.33E-04	-	8.23E-04	2.67E-04	-	1.23E-03	4.00E-04	-	1.65E-03	5.34E-04
95	C17 /II/21/22	1.43E-05	-	4.66E-05	7.16E-05	-	2.33E-04	1.43E-04	-	4.66E-04	2.15E-04	-	6.99E-04	2.86E-04	-	9.32E-04
96	C18 /II/21/22	5.75E-06	5.68E-04	3.47E-05	2.88E-05	2.84E-03	1.74E-04	5.75E-05	5.68E-03	3.47E-04	8.63E-05	8.52E-03	5.21E-04	1.15E-04	1.14E-02	6.94E-04
97	C21 /II/21/22	-	1.08E-04	4.42E-05	-	5.38E-04	2.21E-04	-	1.08E-03	4.42E-04	-	1.61E-03	6.63E-04	-	2.15E-03	8.84E-04
98	C39 /II/21/22	-	1.17E-04	3.10E-05	-	5.86E-04	1.55E-04	-	1.17E-03	3.10E-04	-	1.76E-03	4.64E-04	-	2.34E-03	6.19E-04
99	C40 /II/21/22	-	1.06E-04	3.90E-05	-	5.28E-04	1.95E-04	-	1.06E-03	3.90E-04	-	1.59E-03	5.85E-04	-	2.11E-03	7.80E-04
100	C41 /II/21/22	1.21E-05	9.42E-05	3.14E-05	6.07E-05	4.71E-04	1.57E-04	1.21E-04	9.42E-04	3.14E-04	1.82E-04	1.41E-03	4.70E-04	2.43E-04	1.88E-03	6.27E-04
101	C43 /II/21/22	6.15E-06	2.36E-04	4.02E-05	3.08E-05	1.18E-03	2.01E-04	6.15E-05	2.36E-03	4.02E-04	9.23E-05	3.54E-03	6.03E-04	1.23E-04	4.72E-03	8.03E-04
102	C44 /II/21/22	-	9.16E-05	4.27E-05	-	4.58E-04	2.14E-04	-	9.16E-04	4.27E-04	-	1.37E-03	6.41E-04	-	1.83E-03	8.55E-04
103	C46 /II/21/22	-	1.55E-04	3.73E-05	-	7.76E-04	1.86E-04	-	1.55E-03	3.73E-04	-	2.33E-03	5.59E-04	-	3.10E-03	7.46E-04
104	C314 /II/17/18	4.84E-03	-	3.15E-04	2.42E-02	-	1.58E-03	4.84E-02	-	3.15E-03	7.26E-02	-	4.73E-03	9.67E-02	-	6.31E-03
105	C315 /II/17/18	5.28E-04	-	1.84E-04	2.64E-03	-	9.22E-04	5.28E-03	-	1.84E-03	7.92E-03	-	2.77E-03	1.06E-02	-	3.69E-03
106	C319 /II/17/18	2.97E-04	-	2.06E-05	1.49E-03	-	1.03E-04	2.97E-03	-	2.06E-04	4.46E-03	-	3.09E-04	5.95E-03	-	4.12E-04
107	C320 /II/17/18	1.69E-04	-	1.05E-04	8.47E-04	-	5.26E-04	1.69E-03	-	1.05E-03	2.54E-03	-	1.58E-03	3.39E-03	-	2.11E-03
108	C321 /II/17/18	5.80E-04	-	1.06E-04	2.90E-03	-	5.28E-04	5.80E-03	-	1.06E-03	8.70E-03	-	1.58E-03	1.16E-02	-	2.11E-03
109	C322 /II/17/18	6.15E-04	-	3.98E-05	3.08E-03	-	1.99E-04	6.15E-03	-	3.98E-04	9.23E-03	-	5.96E-04	1.23E-02	-	7.95E-04
110	C323 /II/17/18	-	1.91E-04	1.07E-05	-	9.53E-04	5.34E-05	-	1.91E-03	1.07E-04	-	2.86E-03	1.60E-04	-	3.81E-03	2.13E-04
111	C324 /II/17/18	5.04E-04	-	1.10E-04	2.52E-03	-	5.50E-04	5.04E-03	-	1.10E-03	7.56E-03	-	1.65E-03	1.01E-02	-	2.20E-03

112	C35 /I/23/24	6.82E-05	-	3.61E-05	3.41E-04	-	1.80E-04	6.82E-04	-	3.61E-04	1.02E-03	-	5.41E-04	1.36E-03	-	7.22E-04
113	C168 /I/22/23	-	-	1.71E-05	-	-	8.54E-05	-	-	1.71E-04	-	-	2.56E-04	-	-	3.42E-04
114	C175 /I/22/23	1.03E-05	-	2.47E-05	5.14E-05	-	1.23E-04	1.03E-04	-	2.47E-04	1.54E-04	-	3.70E-04	2.06E-04	-	4.93E-04
115	C100 /II/21/22	6.79E-06	-	5.51E-05	3.40E-05	-	2.76E-04	6.79E-05	-	5.51E-04	1.02E-04	-	8.27E-04	1.36E-04	-	1.10E-03
116	C20 /II/21/22	7.89E-06	1.52E-04	4.00E-05	3.95E-05	7.59E-04	2.00E-04	7.89E-05	1.52E-03	4.00E-04	1.18E-04	2.28E-03	6.00E-04	1.58E-04	3.03E-03	7.99E-04
117	C316 /II/17/18	3.11E-04	-	3.53E-05	1.56E-03	-	1.77E-04	3.11E-03	-	3.53E-04	4.67E-03	-	5.30E-04	6.23E-03	-	7.07E-04
118	C317 /II/17/18	4.13E-04	-	2.28E-05	2.07E-03	-	1.14E-04	4.13E-03	-	2.28E-04	6.20E-03	-	3.42E-04	8.27E-03	-	4.56E-04
119	C326 /II/17/18	2.78E-04	2.02E-04	1.49E-04	1.39E-03	1.01E-03	7.45E-04	2.78E-03	2.02E-03	1.49E-03	4.16E-03	3.03E-03	2.24E-03	5.55E-03	4.04E-03	2.98E-03

Table S8. Lifetime Cancer Risk (LCR) for inhalation exposure to lead, nickel, and cadmium contained in the cigarettes tested.

No.	Sample code	1 cigarette per day			5 cigarettes per day			10 cigarettes per day			15 cigarettes per day			20 cigarettes per day		
		Pb	Ni	Cd	Pb	Ni	Cd	Pb	Ni	Cd	Pb	Ni	Cd	Pb	Ni	Cd
1	C20 /I/23/24	5.96E+00	-	1.69E-02	2.98E+01	-	8.46E-02	5.96E+01	-	1.69E-01	8.94E+01	-	2.54E-01	1.19E+02	-	3.38E-01
2	C21 /I/23/24	2.00E+00	-	1.29E-02	1.00E+01	-	6.43E-02	2.00E+01	-	1.29E-01	3.00E+01	-	1.93E-01	4.00E+01	-	2.57E-01
3	C22 /I/23/24	4.74E+00	-	1.60E-02	2.37E+01	-	8.00E-02	4.74E+01	-	1.60E-01	7.12E+01	-	2.40E-01	9.49E+01	-	3.20E-01
4	C23 /I/23/24	4.34E+00	-	1.88E-02	2.17E+01	-	9.41E-02	4.34E+01	-	1.88E-01	6.51E+01	-	2.82E-01	8.67E+01	-	3.77E-01
5	C24 /I/23/24	5.04E-01	-	1.51E-02	2.52E+00	-	7.55E-02	5.04E+00	-	1.51E-01	7.56E+00	-	2.26E-01	1.01E+01	-	3.02E-01
6	C25 /I/23/24	3.96E+00	-	1.41E-02	1.98E+01	-	7.04E-02	3.96E+01	-	1.41E-01	5.94E+01	-	2.11E-01	7.92E+01	-	2.82E-01
7	C26 /I/23/24	4.03E+00	-	2.96E-02	2.01E+01	-	1.48E-01	4.03E+01	-	2.96E-01	6.04E+01	-	4.44E-01	8.06E+01	-	5.92E-01
8	C27 /I/23/24	2.35E+00	-	2.26E-02	1.17E+01	-	1.13E-01	2.35E+01	-	2.26E-01	3.52E+01	-	3.39E-01	4.69E+01	-	4.53E-01
9	C28 /I/23/24	3.26E+00	-	1.52E-02	1.63E+01	-	7.59E-02	3.26E+01	-	1.52E-01	4.89E+01	-	2.28E-01	6.52E+01	-	3.04E-01
10	C29 /I/23/24	1.95E+00	-	2.22E-02	9.76E+00	-	1.11E-01	1.95E+01	-	2.22E-01	2.93E+01	-	3.34E-01	3.90E+01	-	4.45E-01
11	C30 /I/23/24	-	-	1.91E-02	-	-	9.53E-02	-	-	1.91E-01	-	-	2.86E-01	-	-	3.81E-01
12	C31 /I/23/24	4.31E+00	-	1.45E-02	2.16E+01	-	7.24E-02	4.31E+01	-	1.45E-01	6.47E+01	-	2.17E-01	8.62E+01	-	2.90E-01
13	C32 /I/23/24	4.33E+00	-	2.11E-02	2.17E+01	-	1.06E-01	4.33E+01	-	2.11E-01	6.50E+01	-	3.17E-01	8.67E+01	-	4.23E-01
14	C33 /I/23/24	3.51E+00	-	1.63E-02	1.75E+01	-	8.17E-02	3.51E+01	-	1.63E-01	5.26E+01	-	2.45E-01	7.01E+01	-	3.27E-01
15	C34 /I/23/24	2.45E+00	-	1.84E-02	1.22E+01	-	9.22E-02	2.45E+01	-	1.84E-01	3.67E+01	-	2.77E-01	4.90E+01	-	3.69E-01
16	C36 /I/23/24	4.40E+00	-	1.98E-02	2.20E+01	-	9.91E-02	4.40E+01	-	1.98E-01	6.60E+01	-	2.97E-01	8.80E+01	-	3.96E-01
17	C37 /I/23/24	8.62E+00	-	2.25E-02	4.31E+01	-	1.12E-01	8.62E+01	-	2.25E-01	1.29E+02	-	3.37E-01	1.72E+02	-	4.49E-01

18	C38 /I/23/24	5.49E+00	-	1.93E-02	2.75E+01	-	9.66E-02	5.49E+01	-	1.93E-01	8.24E+01	-	2.90E-01	1.10E+02	-	3.86E-01
19	C39 /I/23/24	3.10E+00	-	1.33E-02	1.55E+01	-	6.63E-02	3.10E+01	-	1.33E-01	4.65E+01	-	1.99E-01	6.20E+01	-	2.65E-01
20	C128 /I/22/23	6.05E-01	3.62E-01	1.49E-02	3.02E+00	1.81E+00	7.47E-02	6.05E+00	3.62E+00	1.49E-01	9.07E+00	5.43E+00	2.24E-01	1.21E+01	7.24E+00	2.99E-01
21	C129 /I/22/23	-	3.57E-01	9.41E-03	-	1.79E+00	4.70E-02	-	3.57E+00	9.41E-02	-	5.36E+00	1.41E-01	-	7.15E+00	1.88E-01
22	C130 /I/22/23	1.06E+00	3.52E-01	1.03E-02	5.30E+00	1.76E+00	5.14E-02	1.06E+01	3.52E+00	1.03E-01	1.59E+01	5.29E+00	1.54E-01	2.12E+01	7.05E+00	2.06E-01
23	C131 /I/22/23	4.86E-01	7.04E-01	7.81E-03	2.43E+00	3.52E+00	3.90E-02	4.86E+00	7.04E+00	7.81E-02	7.29E+00	1.06E+01	1.17E-01	9.71E+00	1.41E+01	1.56E-01
24	C132 /I/22/23	1.17E+00	1.46E+00	7.59E-02	5.83E+00	7.32E+00	3.79E-01	1.17E+01	1.46E+01	7.59E-01	1.75E+01	2.20E+01	1.14E+00	2.33E+01	2.93E+01	1.52E+00
25	C133 /I/22/23	-	1.23E+00	9.42E-03	-	6.15E+00	4.71E-02	-	1.23E+01	9.42E-02	-	1.84E+01	1.41E-01	-	2.46E+01	1.88E-01
26	C134 /I/22/23	-	-	2.20E-02	-	-	1.10E-01	-	-	2.20E-01	-	-	3.30E-01	-	-	4.40E-01
27	C135 /I/22/23	-	9.11E-01	1.30E-02	-	4.56E+00	6.49E-02	-	9.11E+00	1.30E-01	-	1.37E+01	1.95E-01	-	1.82E+01	2.60E-01
28	C136 /I/22/23	-	1.62E+00	2.22E-02	-	8.10E+00	1.11E-01	-	1.62E+01	2.22E-01	-	2.43E+01	3.33E-01	-	3.24E+01	4.43E-01
29	C137 /I/22/23	7.76E-01	1.80E+00	8.92E-03	3.88E+00	8.98E+00	4.46E-02	7.76E+00	1.80E+01	8.92E-02	1.16E+01	2.69E+01	1.34E-01	1.55E+01	3.59E+01	1.78E-01
30	C138 /I/22/23	-	9.26E-01	1.10E-02	-	4.63E+00	5.52E-02	-	9.26E+00	1.10E-01	-	1.39E+01	1.66E-01	-	1.85E+01	2.21E-01
31	C169 /I/22/23	1.05E+00	-	2.82E-02	5.27E+00	-	1.41E-01	1.05E+01	-	2.82E-01	1.58E+01	-	4.23E-01	2.11E+01	-	5.64E-01
32	C170 /I/22/23	-	3.82E-01	1.17E-02	-	1.91E+00	5.83E-02	-	3.82E+00	1.17E-01	-	5.72E+00	1.75E-01	-	7.63E+00	2.33E-01
33	C171 /I/22/23	-	3.04E-01	1.43E-02	-	1.52E+00	7.17E-02	-	3.04E+00	1.43E-01	-	4.56E+00	2.15E-01	-	6.08E+00	2.87E-01
34	C172 /I/22/23	-	-	1.15E-02	-		5.75E-02	-	-	1.15E-01	-		1.73E-01	-	-	2.30E-01
35	C173 /I/22/23	-	3.72E-01	1.31E-02	-	1.86E+00	6.57E-02	-	3.72E+00	1.31E-01	-	5.58E+00	1.97E-01	-	7.44E+00	2.63E-01
36	C174 /I/22/23	-	3.84E-01	1.89E-02	-	1.92E+00	9.43E-02	-	3.84E+00	1.89E-01	-	5.76E+00	2.83E-01	-	7.68E+00	3.77E-01

37	C176 /I/22/23	6.88E-01	3.73E-01	1.71E-02	3.44E+00	1.86E+00	8.57E-02	6.88E+00	3.73E+00	1.71E-01	1.03E+01	5.59E+00	2.57E-01	1.38E+01	7.45E+00	3.43E-01
38	C177 /I/22/23	-	2.62E-01	1.72E-02		1.31E+00	8.62E-02	-	2.62E+00	1.72E-01	-	3.93E+00	2.59E-01		5.24E+00	3.45E-01
39	C178 /I/22/23	9.18E-01	2.99E-01	2.13E-02	4.59E+00	1.49E+00	1.06E-01	9.18E+00	2.99E+00	2.13E-01	1.38E+01	4.48E+00	3.19E-01	1.84E+01	5.97E+00	4.26E-01
40	C179 /I/22/23	-	-	1.53E-02	-	-	7.67E-02	-	-	1.53E-01	-	-	2.30E-01	-	-	3.07E-01
41	C180 /I/22/23	-	4.53E-01	1.47E-02	-	2.27E+00	7.34E-02	-	4.53E+00	1.47E-01	-	6.80E+00	2.20E-01	-	9.06E+00	2.93E-01
42	C181 /I/22/23	5.09E-01	3.90E-01	9.77E-03	2.54E+00	1.95E+00	4.89E-02	5.09E+00	3.90E+00	9.77E-02	7.63E+00	5.84E+00	1.47E-01	1.02E+01	7.79E+00	1.95E-01
43	C182 /I/22/23	8.84E-01	3.36E-01	1.26E-02	4.42E+00	1.68E+00	6.31E-02	8.84E+00	3.36E+00	1.26E-01	1.33E+01	5.04E+00	1.89E-01	1.77E+01	6.72E+00	2.53E-01
44	C183 /I/22/23	9.46E-01	-	1.47E-02	4.73E+00	-	7.33E-02	9.46E+00	-	1.47E-01	1.42E+01	-	2.20E-01	1.89E+01	-	2.93E-01
45	C184 /I/22/23	1.31E+00	-	8.27E-03	6.55E+00	-	4.14E-02	1.31E+01	-	8.27E-02	1.97E+01	-	1.24E-01	2.62E+01	-	1.65E-01
46	C185 /I/22/23	4.99E-01	-	6.34E-03	2.49E+00	-	3.17E-02	4.99E+00	-	6.34E-02	7.48E+00	-	9.51E-02	9.97E+00	-	1.27E-01
47	C186 /I/22/23	-	3.40E-01	8.45E-03	-	1.70E+00	4.23E-02	-	3.40E+00	8.45E-02	-	5.11E+00	1.27E-01	-	6.81E+00	1.69E-01
48	C187 /I/22/23	-	2.97E-01	1.12E-02	-	1.49E+00	5.59E-02	-	2.97E+00	1.12E-01	-	4.46E+00	1.68E-01	-	5.94E+00	2.24E-01
49	C188 /I/22/23	1.91E+00	3.95E-01	7.18E-03	9.55E+00	1.98E+00	3.59E-02	1.91E+01	3.95E+00	7.18E-02	2.86E+01	5.93E+00	1.08E-01	3.82E+01	7.91E+00	1.44E-01
50	C64 /II/22/23	-	3.53E-01	1.71E-02	-	1.76E+00	8.55E-02	-	3.53E+00	1.71E-01	-	5.29E+00	2.57E-01	-	7.06E+00	3.42E-01
51	C65 /II/22/23	-	1.61E+00	1.55E-02	-	8.05E+00	7.75E-02	-	1.61E+01	1.55E-01	-	2.41E+01	2.32E-01	-	3.22E+01	3.10E-01
52	C66 /II/22/23	-	-	1.76E-02	-	-	8.82E-02	-	-	1.76E-01	-	-	2.64E-01	-	-	3.53E-01
53	C67 /II/22/23	-	4.32E-01	1.52E-02	-	2.16E+00	7.58E-02	-	4.32E+00	1.52E-01	-	6.49E+00	2.27E-01	-	8.65E+00	3.03E-01
54	C68 /II/22/23	-	-	1.29E-02	-	-	6.47E-02	-	-	1.29E-01	-	-	1.94E-01	-	-	2.59E-01
55	C69 /II/22/23	-	4.83E-01	1.25E-02	-	2.41E+00	6.25E-02	-	4.83E+00	1.25E-01	-	7.24E+00	1.88E-01	-	9.66E+00	2.50E-01

56	C70 /II/22/23	-	-	1.44E-02	-	-	7.19E-02	-	-	1.44E-01	-	-	2.16E-01	-	-	2.88E-01
57	C128 /II/22/23	5.01E+03	-	1.30E-02	2.50E+04	-	6.48E-02	5.01E+04	-	1.30E-01	7.51E+04	-	1.94E-01	1.00E+05	-	2.59E-01
58	C129 /II/22/23	2.59E+03	-	1.43E-02	1.30E+04	-	7.13E-02	2.59E+04	-	1.43E-01	3.89E+04	-	2.14E-01	5.19E+04	-	2.85E-01
59	C130 /II/22/23	1.28E+03	-	1.32E-02	6.39E+03	-	6.61E-02	1.28E+04	-	1.32E-01	1.92E+04	-	1.98E-01	2.56E+04	-	2.64E-01
60	C131 /II/22/23	3.64E+02	-	1.97E-02	1.82E+03	-	9.83E-02	3.64E+03	-	1.97E-01	5.46E+03	-	2.95E-01	7.28E+03	-	3.93E-01
61	C133 /II/22/23	2.09E+02	-	1.32E-02	1.05E+03	-	6.62E-02	2.09E+03	-	1.32E-01	3.14E+03	-	1.98E-01	4.19E+03	-	2.65E-01
62	C134 /II/22/23	4.23E+02	-	1.49E-02	2.12E+03	-	7.46E-02	4.23E+03	-	1.49E-01	6.35E+03	-	2.24E-01	8.47E+03	-	2.98E-01
63	C135 /II/22/23	1.81E+02	-	1.16E-02	9.04E+02	-	5.79E-02	1.81E+03	-	1.16E-01	2.71E+03	-	1.74E-01	3.61E+03	-	2.32E-01
64	C136 /II/22/23	1.12E+02	9.20E-01	2.08E-02	5.62E+02	4.60E+00	1.04E-01	1.12E+03	9.20E+00	2.08E-01	1.69E+03	1.38E+01	3.12E-01	2.25E+03	1.84E+01	4.16E-01
65	C137 /II/22/23	3.90E+01	7.07E-01	9.84E-03	1.95E+02	3.54E+00	4.92E-02	3.90E+02	7.07E+00	9.84E-02	5.85E+02	1.06E+01	1.48E-01	7.80E+02	1.41E+01	1.97E-01
66	C138 /II/22/23	4.74E+01	1.07E+00	2.08E-02	2.37E+02	5.37E+00	1.04E-01	4.74E+02	1.07E+01	2.08E-01	7.12E+02	1.61E+01	3.12E-01	9.49E+02	2.15E+01	4.16E-01
67	C139 /II/22/23	2.91E+01	-	1.22E-02	1.45E+02	-	6.10E-02	2.91E+02	-	1.22E-01	4.36E+02	-	1.83E-01	5.82E+02	-	2.44E-01
68	C140 /II/22/23	2.26E+01	-	1.08E-02	1.13E+02	-	5.41E-02	2.26E+02	-	1.08E-01	3.39E+02	-	1.62E-01	4.52E+02	-	2.16E-01
69	C141 /II/22/23	2.45E+01	-	1.06E-02	1.22E+02	-	5.29E-02	2.45E+02	-	1.06E-01	3.67E+02	-	1.59E-01	4.90E+02	-	2.12E-01
70	C142 /II/22/23	5.76E+01	-	1.32E-02	2.88E+02	-	6.60E-02	5.76E+02	-	1.32E-01	8.64E+02	-	1.98E-01	1.15E+03	-	2.64E-01
71	C143 /II/22/23	-	-	1.66E-02	-	-	8.28E-02	-	-	1.66E-01	-	-	2.48E-01	-	-	3.31E-01
72	C144 /II/22/23	7.36E+01	-	1.31E-02	3.68E+02	-	6.56E-02	7.36E+02	-	1.31E-01	1.10E+03	-	1.97E-01	1.47E+03	-	2.62E-01
73	C145 /II/22/23	2.05E+01	-	2.16E-02	1.02E+02	-	1.08E-01	2.05E+02	-	2.16E-01	3.07E+02	-	3.24E-01	4.09E+02	-	4.32E-01
74	C146 /II/22/23	4.44E+01	2.56E-01	1.86E-02	2.22E+02	1.28E+00	9.29E-02	4.44E+02	2.56E+00	1.86E-01	6.66E+02	3.84E+00	2.79E-01	8.87E+02	5.12E+00	3.72E-01

75	C147 /II/22/23	1.45E+01	-	1.25E-02	7.27E+01	-	6.25E-02	1.45E+02	-	1.25E-01	2.18E+02	-	1.88E-01	2.91E+02	-	2.50E-01
76	C148 /II/22/23	1.25E+01	-	1.54E-02	6.24E+01	-	7.69E-02	1.25E+02	-	1.54E-01	1.87E+02	-	2.31E-01	2.49E+02	-	3.07E-01
77	C149 /II/22/23	-	-	1.79E-02	-	-	8.94E-02	-	-	1.79E-01	-	-	2.68E-01	-	-	3.57E-01
78	C15 /II/22/23	3.14E+01	-	1.80E-02	1.57E+02	-	9.02E-02	3.14E+02	-	1.80E-01	4.71E+02	-	2.71E-01	6.28E+02	-	3.61E-01
79	C86 /II/21/22	1.12E+00	-	1.89E-02	5.61E+00	-	9.47E-02	1.12E+01	-	1.89E-01	1.68E+01	-	2.84E-01	2.25E+01	-	3.79E-01
80	C87 /II/21/22	-	-	1.35E-02	-	-	6.73E-02	-	-	1.35E-01	-	-	2.02E-01	-	-	2.69E-01
81	C88 /II/21/22	7.00E-01	-	1.86E-02	3.50E+00	-	9.30E-02	7.00E+00	-	1.86E-01	1.05E+01	-	2.79E-01	1.40E+01	-	3.72E-01
82	C89 /II/21/22	7.66E-01	2.69E-01	9.74E-03	3.83E+00	1.35E+00	4.87E-02	7.66E+00	2.69E+00	9.74E-02	1.15E+01	4.04E+00	1.46E-01	1.53E+01	5.38E+00	1.95E-01
83	C90 /II/21/22	7.63E-01	3.97E-01	1.95E-02	3.82E+00	1.99E+00	9.76E-02	7.63E+00	3.97E+00	1.95E-01	1.14E+01	5.96E+00	2.93E-01	1.53E+01	7.94E+00	3.90E-01
84	C91 /II/21/22	-	-	1.79E-02	-	-	8.94E-02	-	-	1.79E-01	-	-	2.68E-01	-	-	3.58E-01
85	C92 /II/21/22	8.98E-01	-	1.83E-02	4.49E+00	-	9.13E-02	8.98E+00	-	1.83E-01	1.35E+01	-	2.74E-01	1.80E+01	-	3.65E-01
86	C93 /II/21/22	5.66E-01	-	9.88E-03	2.83E+00	-	4.94E-02	5.66E+00	-	9.88E-02	8.48E+00	-	1.48E-01	1.13E+01	-	1.98E-01
87	C94 /II/21/22	6.59E-01	2.72E-01	9.25E-03	3.29E+00	1.36E+00	4.63E-02	6.59E+00	2.72E+00	9.25E-02	9.88E+00	4.07E+00	1.39E-01	1.32E+01	5.43E+00	1.85E-01
88	C95 /II/21/22	5.93E-01	-	7.89E-03	2.96E+00	-	3.95E-02	5.93E+00	-	7.89E-02	8.89E+00	-	1.18E-01	1.19E+01	-	1.58E-01
89	C96 /II/21/22	-	-	1.18E-02	-	-	5.91E-02	-	-	1.18E-01	-	-	1.77E-01	-	-	2.37E-01
90	C97 /II/21/22	-	2.86E-01	1.28E-02	-	1.43E+00	6.40E-02	-	2.86E+00	1.28E-01	-	4.28E+00	1.92E-01	-	5.71E+00	2.56E-01
91	C98 /II/21/22	-	-	1.22E-02	-	-	6.11E-02	-	-	1.22E-01	-	-	1.83E-01	-	-	2.45E-01
92	C99 /II/21/22	7.73E-01	2.67E-01	1.99E-02	3.86E+00	1.33E+00	9.96E-02	7.73E+00	2.67E+00	1.99E-01	1.16E+01	4.00E+00	2.99E-01	1.55E+01	5.34E+00	3.98E-01
93	C101 /II/21/22	8.52E-01	3.00E-01	2.02E-02	4.26E+00	1.50E+00	1.01E-01	8.52E+00	3.00E+00	2.02E-01	1.28E+01	4.49E+00	3.03E-01	1.70E+01	5.99E+00	4.04E-01

94	C16 /II/21/22	-	3.43E-01	1.48E-02	-	1.71E+00	7.41E-02	-	3.43E+00	1.48E-01	-	5.14E+00	2.22E-01	-	6.86E+00	2.96E-01
95	C17 /II/21/22	1.19E+00	-	2.59E-02	5.97E+00	-	1.29E-01	1.19E+01		2.59E-01	1.79E+01	-	3.88E-01	2.39E+01	-	5.18E-01
96	C18 /II/21/22	4.79E-01	2.37E+00	1.93E-02	2.40E+00	1.18E+01	9.64E-02	4.79E+00	2.37E+01	1.93E-01	7.19E+00	3.55E+01	2.89E-01	9.59E+00	4.74E+01	3.86E-01
97	C21 /II/21/22	-	4.48E-01	2.46E-02	-	2.24E+00	1.23E-01	-	4.48E+00	2.46E-01	-	6.73E+00	3.68E-01	-	8.97E+00	4.91E-01
98	C39 /II/21/22	-	4.88E-01	1.72E-02	-	2.44E+00	8.60E-02	-	4.88E+00	1.72E-01	-	7.32E+00	2.58E-01	-	9.77E+00	3.44E-01
99	C40 /II/21/22	-	4.40E-01	2.17E-02	-	2.20E+00	1.08E-01	-	4.40E+00	2.17E-01	-	6.61E+00	3.25E-01	-	8.81E+00	4.33E-01
100	C41 /II/21/22	1.01E+00	3.93E-01	1.74E-02	5.06E+00	1.96E+00	8.71E-02	1.01E+01	3.93E+00	1.74E-01	1.52E+01	5.89E+00	2.61E-01	2.02E+01	7.85E+00	3.48E-01
101	C43 /II/21/22	5.13E-01	9.84E-01	2.23E-02	2.56E+00	4.92E+00	1.12E-01	5.13E+00	9.84E+00	2.23E-01	7.69E+00	1.48E+01	3.35E-01	1.03E+01	1.97E+01	4.46E-01
102	C44 /II/21/22	-	3.82E-01	2.37E-02	-	1.91E+00	1.19E-01	-	3.82E+00	2.37E-01	-	5.73E+00	3.56E-01	-	7.64E+00	4.75E-01
103	C46 /II/21/22	-	6.47E-01	2.07E-02	-	3.23E+00	1.04E-01	-	6.47E+00	2.07E-01	-	9.70E+00	3.11E-01	-	1.29E+01	4.14E-01
104	C314 /II/17/18	4.03E+02	-	1.75E-01	2.02E+03	-	8.76E-01	4.03E+03	-	1.75E+00	6.05E+03	-	2.63E+00	8.06E+03	-	3.50E+00
105	C315 /II/17/18	4.40E+01	-	1.02E-01	2.20E+02	-	5.12E-01	4.40E+02	-	1.02E+00	6.60E+02	-	1.54E+00	8.80E+02	-	2.05E+00
106	C319 /II/17/18	2.48E+01	-	1.15E-02	1.24E+02	-	5.73E-02	2.48E+02	-	1.15E-01	3.72E+02	-	1.72E-01	4.96E+02	-	2.29E-01
107	C320 /II/17/18	1.41E+01	-	5.85E-02	7.05E+01	-	2.92E-01	1.41E+02	-	5.85E-01	2.12E+02	-	8.77E-01	2.82E+02	-	1.17E+00
108	C321 /II/17/18	4.83E+01	-	5.87E-02	2.42E+02	-	2.93E-01	4.83E+02	-	5.87E-01	7.25E+02	-	8.80E-01	9.66E+02	-	1.17E+00
109	C322 /II/17/18	5.13E+01	-	2.21E-02	2.56E+02	-	1.10E-01	5.13E+02	-	2.21E-01	7.69E+02	-	3.31E-01	1.03E+03	-	4.42E-01
110	C323 /II/17/18	-	7.94E-01	5.93E-03	-	3.97E+00	2.96E-02	-	7.94E+00	5.93E-02	-	1.19E+01	8.89E-02	-	1.59E+01	1.19E-01
111	C324 /II/17/18	4.20E+01	-	6.11E-02	2.10E+02	-	3.06E-01	4.20E+02	-	6.11E-01	6.30E+02	-	9.17E-01	8.40E+02	-	1.22E+00

112	C35 /I/23/24	5.68E+00	-	2.00E-02	2.84E+01	-	1.00E-01	5.68E+01	-	2.00E-01	8.52E+01	-	3.01E-01	1.14E+02	-	4.01E-01
113	C168 /I/22/23	-	-	9.49E-03	-	-	4.75E-02	-	-	9.49E-02	-	-	1.42E-01	-	-	1.90E-01
114	C175 /I/22/23	8.57E-01	-	1.37E-02	4.28E+00	-	6.85E-02	8.57E+00	-	1.37E-01	1.29E+01	-	2.06E-01	1.71E+01	-	2.74E-01
115	C100 /II/21/22	5.66E-01	-	3.06E-02	2.83E+00	-	1.53E-01	5.66E+00	-	3.06E-01	8.49E+00	-	4.59E-01	1.13E+01	-	6.13E-01
116	C20 /II/21/22	6.58E-01	6.32E-01	2.22E-02	3.29E+00	3.16E+00	1.11E-01	6.58E+00	6.32E+00	2.22E-01	9.86E+00	9.48E+00	3.33E-01	1.32E+01	1.26E+01	4.44E-01
117	C316 /II/17/18	2.59E+01	-	1.96E-02	1.30E+02	-	9.82E-02	2.59E+02	-	1.96E-01	3.89E+02	-	2.94E-01	5.19E+02	-	3.93E-01
118	C317 /II/17/18	3.44E+01	-	1.27E-02	1.72E+02	-	6.34E-02	3.44E+02	-	1.27E-01	5.17E+02	-	1.90E-01	6.89E+02	-	2.54E-01
119	C326 /II/17/18	2.31E+01	8.41E-01	8.28E-02	1.16E+02	4.21E+00	4.14E-01	2.31E+02	8.41E+00	8.28E-01	3.47E+02	1.26E+01	1.24E+00	4.63E+02	1.68E+01	1.66E+00

Table S9. Lifetime Average Daily Dose (LADD) [mg/kg] for ingestion exposure to lead, nickel, and cadmium contained in the cigarettes tested.

No.	Sample code	1 cigarette per day			5 cigarettes per day			10 cigarettes per day			15 cigarettes per day			20 cigarettes per day		
		Pb	Ni	Cd	Pb	Ni	Cd	Pb	Ni	Cd	Pb	Ni	Cd	Pb	Ni	Cd
1	C20 /I/23/24	7.02E-07	-	2.99E-07	3.51E-06	-	1.49E-06	7.02E-06	-	2.99E-06	1.05E-05	-	4.48E-06	1.40E-05	-	5.98E-06
2	C21 /I/23/24	2.36E-07	-	2.27E-07	1.18E-06	-	1.14E-06	2.36E-06	-	2.27E-06	3.54E-06	-	3.41E-06	4.71E-06	-	4.54E-06
3	C22 /I/23/24	5.59E-07	-	2.83E-07	2.79E-06	-	1.41E-06	5.59E-06	-	2.83E-06	8.38E-06	-	4.24E-06	1.12E-05	-	5.66E-06
4	C23 /I/23/24	5.11E-07	-	3.33E-07	2.55E-06	-	1.66E-06	5.11E-06	-	3.33E-06	7.66E-06	-	4.99E-06	1.02E-05	-	6.65E-06
5	C24 /I/23/24	5.94E-08	-	2.67E-07	2.97E-07	-	1.33E-06	5.94E-07	-	2.67E-06	8.90E-07	-	4.00E-06	1.19E-06	-	5.33E-06
6	C25 /I/23/24	4.66E-07	-	2.49E-07	2.33E-06	-	1.24E-06	4.66E-06	-	2.49E-06	7.00E-06	-	3.73E-06	9.33E-06	-	4.98E-06
7	C26 /I/23/24	4.74E-07	-	5.23E-07	2.37E-06	-	2.62E-06	4.74E-06	-	5.23E-06	7.12E-06	-	7.85E-06	9.49E-06	-	1.05E-05
8	C27 /I/23/24	2.76E-07	-	4.00E-07	1.38E-06	-	2.00E-06	2.76E-06	-	4.00E-06	4.15E-06	-	6.00E-06	5.53E-06	-	7.99E-06
9	C28 /I/23/24	3.84E-07	-	2.68E-07	1.92E-06	-	1.34E-06	3.84E-06	-	2.68E-06	5.76E-06	-	4.02E-06	7.68E-06	-	5.36E-06
10	C29 /I/23/24	2.30E-07	-	3.93E-07	1.15E-06	-	1.96E-06	2.30E-06	-	3.93E-06	3.45E-06	-	5.89E-06	4.60E-06	-	7.86E-06
11	C30 /I/23/24	-	-	3.37E-07	-	-	1.68E-06	-	-	3.37E-06	-	-	5.05E-06	-	-	6.73E-06
12	C31 /I/23/24	5.08E-07	-	2.56E-07	2.54E-06	-	1.28E-06	5.08E-06	-	2.56E-06	7.62E-06	-	3.84E-06	1.02E-05	-	5.12E-06
13	C32 /I/23/24	5.11E-07	-	3.73E-07	2.55E-06	-	1.87E-06	5.11E-06	-	3.73E-06	7.66E-06	-	5.60E-06	1.02E-05	-	7.47E-06
14	C33 /I/23/24	4.13E-07	-	2.89E-07	2.06E-06	-	1.44E-06	4.13E-06	-	2.89E-06	6.19E-06	-	4.33E-06	8.26E-06	-	5.77E-06
15	C34 /I/23/24	2.88E-07	-	3.26E-07	1.44E-06	-	1.63E-06	2.88E-06	-	3.26E-06	4.32E-06	-	4.88E-06	5.76E-06	-	6.51E-06
16	C36 /I/23/24	5.18E-07	-	3.50E-07	2.59E-06	-	1.75E-06	5.18E-06	-	3.50E-06	7.78E-06	-	5.25E-06	1.04E-05	-	7.00E-06
17	C37 /I/23/24	1.02E-06	-	3.97E-07	5.08E-06	-	1.98E-06	1.02E-05	-	3.97E-06	1.52E-05	-	5.95E-06	2.03E-05	-	7.94E-06

18	C38 /I/23/24	6.47E-07	-	3.41E-07	3.23E-06	-	1.71E-06	6.47E-06	-	3.41E-06	9.70E-06	-	5.12E-06	1.29E-05	-	6.83E-06
19	C39 /I/23/24	3.65E-07	-	2.34E-07	1.83E-06	-	1.17E-06	3.65E-06	-	2.34E-06	5.48E-06	-	3.52E-06	7.30E-06	-	4.69E-06
20	C128 /I/22/23	7.12E-08	8.52E-07	2.64E-07	3.56E-07	4.26E-06	1.32E-06	7.12E-07	8.52E-06	2.64E-06	1.07E-06	1.28E-05	3.96E-06	1.42E-06	1.70E-05	5.28E-06
21	C129 /I/22/23	-	8.42E-07	1.66E-07	-	4.21E-06	8.31E-07	-	8.42E-06	1.66E-06	-	1.26E-05	2.49E-06	-	1.68E-05	3.32E-06
22	C130 /I/22/23	1.25E-07	8.30E-07	1.82E-07	6.24E-07	4.15E-06	9.08E-07	1.25E-06	8.30E-06	1.82E-06	1.87E-06	1.25E-05	2.72E-06	2.50E-06	1.66E-05	3.63E-06
23	C131 /I/22/23	5.72E-08	1.66E-06	1.38E-07	2.86E-07	8.29E-06	6.89E-07	5.72E-07	1.66E-05	1.38E-06	8.58E-07	2.49E-05	2.07E-06	1.14E-06	3.32E-05	2.76E-06
24	C132 /I/22/23	1.37E-07	3.45E-06	1.34E-06	6.87E-07	1.72E-05	6.70E-06	1.37E-06	3.45E-05	1.34E-05	2.06E-06	5.17E-05	2.01E-05	2.75E-06	6.90E-05	2.68E-05
25	C133 /I/22/23	-	2.90E-06	1.66E-07	-	1.45E-05	8.32E-07	-	2.90E-05	1.66E-06	-	4.34E-05	2.50E-06	-	5.79E-05	3.33E-06
26	C134 /I/22/23	-	-	3.88E-07	-	-	1.94E-06	-	-	3.88E-06	-	-	5.82E-06	-	-	7.77E-06
27	C135 /I/22/23	-	2.15E-06	2.29E-07	-	1.07E-05	1.15E-06	-	2.15E-05	2.29E-06	-	3.22E-05	3.44E-06	-	4.29E-05	4.59E-06
28	C136 /I/22/23	-	3.81E-06	3.92E-07	-	1.91E-05	1.96E-06	-	3.81E-05	3.92E-06	-	5.72E-05	5.87E-06	-	7.63E-05	7.83E-06
29	C137 /I/22/23	9.14E-08	4.23E-06	1.58E-07	4.57E-07	2.11E-05	7.88E-07	9.14E-07	4.23E-05	1.58E-06	1.37E-06	6.34E-05	2.36E-06	1.83E-06	8.46E-05	3.15E-06
30	C138 /I/22/23	-	2.18E-06	1.95E-07	-	1.09E-05	9.76E-07	-	2.18E-05	1.95E-06	-	3.27E-05	2.93E-06	-	4.36E-05	3.90E-06
31	C169 /I/22/23	1.24E-07	-	4.98E-07	6.21E-07	-	2.49E-06	1.24E-06	-	4.98E-06	1.86E-06	-	7.47E-06	2.48E-06	-	9.96E-06
32	C170 /I/22/23	-	8.99E-07	2.06E-07	-	4.49E-06	1.03E-06	-	8.99E-06	2.06E-06	-	1.35E-05	3.09E-06	-	1.80E-05	4.12E-06
33	C171 /I/22/23	-	7.16E-07	2.53E-07	-	3.58E-06	1.27E-06	-	7.16E-06	2.53E-06	-	1.07E-05	3.80E-06	-	1.43E-05	5.06E-06
34	C172 /I/22/23	-	-	2.03E-07	-	-	1.02E-06	-	-	2.03E-06	-	-	3.05E-06	-	-	4.06E-06
35	C173 /I/22/23	-	8.77E-07	2.32E-07	-	4.38E-06	1.16E-06	-	8.77E-06	2.32E-06	-	1.31E-05	3.48E-06	-	1.75E-05	4.64E-06
36	C174 /I/22/23	-	9.05E-07	3.33E-07	-	4.52E-06	1.67E-06	-	9.05E-06	3.33E-06	-	1.36E-05	5.00E-06	-	1.81E-05	6.66E-06

37	C176 /I/22/23	8.11E-08	8.78E-07	3.03E-07	4.05E-07	4.39E-06	1.51E-06	8.11E-07	8.78E-06	3.03E-06	1.22E-06	1.32E-05	4.54E-06	1.62E-06	1.76E-05	6.05E-06
38	C177 /I/22/23	-	6.18E-07	3.04E-07	-	3.09E-06	1.52E-06	-	6.18E-06	3.04E-06	-	9.26E-06	4.57E-06	-	1.24E-05	6.09E-06
39	C178 /I/22/23	1.08E-07	7.03E-07	3.76E-07	5.41E-07	3.52E-06	1.88E-06	1.08E-06	7.03E-06	3.76E-06	1.62E-06	1.05E-05	5.64E-06	2.16E-06	1.41E-05	7.52E-06
40	C179 /I/22/23	-	-	2.71E-07	-	-	1.35E-06	-	-	2.71E-06	-	-	4.06E-06	-	-	5.42E-06
41	C180 /I/22/23	-	1.07E-06	2.59E-07	-	5.34E-06	1.30E-06	-	1.07E-05	2.59E-06	-	1.60E-05	3.89E-06	-	2.14E-05	5.18E-06
42	C181 /I/22/23	5.99E-08	9.18E-07	1.73E-07	3.00E-07	4.59E-06	8.63E-07	5.99E-07	9.18E-06	1.73E-06	8.99E-07	1.38E-05	2.59E-06	1.20E-06	1.84E-05	3.45E-06
43	C182 /I/22/23	1.04E-07	7.91E-07	2.23E-07	5.21E-07	3.96E-06	1.12E-06	1.04E-06	7.91E-06	2.23E-06	1.56E-06	1.19E-05	3.35E-06	2.08E-06	1.58E-05	4.46E-06
44	C183 /I/22/23	1.11E-07	-	2.59E-07	5.57E-07	-	1.29E-06	1.11E-06	-	2.59E-06	1.67E-06	-	3.88E-06	2.23E-06	-	5.18E-06
45	C184 /I/22/23	1.54E-07	-	1.46E-07	7.71E-07	-	7.31E-07	1.54E-06	-	1.46E-06	2.31E-06	-	2.19E-06	3.09E-06	-	2.92E-06
46	C185 /I/22/23	5.87E-08	-	1.12E-07	2.94E-07	-	5.60E-07	5.87E-07	-	1.12E-06	8.81E-07	-	1.68E-06	1.17E-06	-	2.24E-06
47	C186 /I/22/23	-	8.02E-07	1.49E-07	-	4.01E-06	7.47E-07	-	8.02E-06	1.49E-06	-	1.20E-05	2.24E-06	-	1.60E-05	2.99E-06
48	C187 /I/22/23	-	7.00E-07	1.98E-07	-	3.50E-06	9.88E-07	-	7.00E-06	1.98E-06	-	1.05E-05	2.96E-06	-	1.40E-05	3.95E-06
49	C188 /I/22/23	2.25E-07	9.31E-07	1.27E-07	1.12E-06	4.66E-06	6.34E-07	2.25E-06	9.31E-06	1.27E-06	3.37E-06	1.40E-05	1.90E-06	4.50E-06	1.86E-05	2.54E-06
50	C64 /II/22/23	-	8.31E-07	3.02E-07	-	4.16E-06	1.51E-06	-	8.31E-06	3.02E-06	-	1.25E-05	4.53E-06	-	1.66E-05	6.04E-06
51	C65 /II/22/23	-	3.79E-06	2.74E-07	-	1.90E-05	1.37E-06	-	3.79E-05	2.74E-06	-	5.69E-05	4.11E-06	-	7.58E-05	5.47E-06
52	C66 /II/22/23	-	-	3.11E-07	-	-	1.56E-06	-	-	3.11E-06	-	-	4.67E-06	-	-	6.23E-06
53	C67 /II/22/23	-	1.02E-06	2.68E-07	-	5.09E-06	1.34E-06	-	1.02E-05	2.68E-06	-	1.53E-05	4.02E-06	-	2.04E-05	5.36E-06
54	C68 /II/22/23	-	-	2.29E-07	-	-	1.14E-06	-	-	2.29E-06	-	-	3.43E-06	-	-	4.58E-06
55	C69 /II/22/23	-	1.14E-06	2.21E-07	-	5.69E-06	1.10E-06	-	1.14E-05	2.21E-06	-	1.71E-05	3.31E-06	-	2.27E-05	4.42E-06

56	C70 /II/22/23	-	-	2.54E-07	-	-	1.27E-06	-	-	2.54E-06	-	-	3.81E-06	-	-	5.08E-06
57	C128 /II/22/23	5.90E-04	-	2.29E-07	2.95E-03	-	1.14E-06	5.90E-03	-	2.29E-06	8.85E-03	-	3.43E-06	1.18E-02	-	4.58E-06
58	C129 /II/22/23	3.06E-04	-	2.52E-07	1.53E-03	-	1.26E-06	3.06E-03	-	2.52E-06	4.58E-03	-	3.78E-06	6.11E-03	-	5.04E-06
59	C130 /II/22/23	1.50E-04	-	2.34E-07	7.52E-04	-	1.17E-06	1.50E-03	-	2.34E-06	2.26E-03	-	3.50E-06	3.01E-03	-	4.67E-06
60	C131 /II/22/23	4.29E-05	-	3.47E-07	2.14E-04	-	1.74E-06	4.29E-04	-	3.47E-06	6.43E-04	-	5.21E-06	8.58E-04	-	6.95E-06
61	C133 /II/22/23	2.47E-05	-	2.34E-07	1.23E-04	-	1.17E-06	2.47E-04	-	2.34E-06	3.70E-04	-	3.51E-06	4.93E-04	-	4.67E-06
62	C134 /II/22/23	4.99E-05	-	2.63E-07	2.49E-04	-	1.32E-06	4.99E-04	-	2.63E-06	7.48E-04	-	3.95E-06	9.97E-04	-	5.27E-06
63	C135 /II/22/23	2.13E-05	-	2.05E-07	1.06E-04	-	1.02E-06	2.13E-04	-	2.05E-06	3.19E-04	-	3.07E-06	4.26E-04	-	4.09E-06
64	C136 /II/22/23	1.32E-05	2.17E-06	3.67E-07	6.62E-05	1.08E-05	1.84E-06	1.32E-04	2.17E-05	3.67E-06	1.99E-04	3.25E-05	5.51E-06	2.65E-04	4.33E-05	7.34E-06
65	C137 /II/22/23	4.60E-06	1.67E-06	1.74E-07	2.30E-05	8.33E-06	8.69E-07	4.60E-05	1.67E-05	1.74E-06	6.89E-05	2.50E-05	2.61E-06	9.19E-05	3.33E-05	3.48E-06
66	C138 /II/22/23	5.59E-06	2.53E-06	3.67E-07	2.79E-05	1.27E-05	1.84E-06	5.59E-05	2.53E-05	3.67E-06	8.38E-05	3.80E-05	5.51E-06	1.12E-04	5.06E-05	7.34E-06
67	C139 /II/22/23	3.43E-06	-	2.15E-07	1.71E-05	-	1.08E-06	3.43E-05	-	2.15E-06	5.14E-05	-	3.23E-06	6.85E-05	-	4.31E-06
68	C140 /II/22/23	2.66E-06	-	1.91E-07	1.33E-05	-	9.55E-07	2.66E-05	-	1.91E-06	3.99E-05	-	2.86E-06	5.32E-05	-	3.82E-06
69	C141 /II/22/23	2.88E-06	-	1.87E-07	1.44E-05	-	9.35E-07	2.88E-05	-	1.87E-06	4.32E-05	-	2.80E-06	5.77E-05	-	3.74E-06
70	C142 /II/22/23	6.78E-06	-	2.33E-07	3.39E-05	-	1.17E-06	6.78E-05	-	2.33E-06	1.02E-04	-	3.50E-06	1.36E-04	-	4.67E-06
71	C143 /II/22/23	-	-	2.93E-07	-	-	1.46E-06	-	-	2.93E-06	-	-	4.39E-06	-	-	5.85E-06
72	C144 /II/22/23	8.66E-06	-	2.32E-07	4.33E-05	-	1.16E-06	8.66E-05	-	2.32E-06	1.30E-04	-	3.47E-06	1.73E-04	-	4.63E-06
73	C145 /II/22/23	2.41E-06	-	3.82E-07	1.20E-05	-	1.91E-06	2.41E-05	-	3.82E-06	3.61E-05	-	5.73E-06	4.82E-05	-	7.63E-06
74	C146 /II/22/23	5.23E-06	6.03E-07	3.28E-07	2.61E-05	3.02E-06	1.64E-06	5.23E-05	6.03E-06	3.28E-06	7.84E-05	9.05E-06	4.92E-06	1.05E-04	1.21E-05	6.57E-06

75	C147 /II/22/23	1.71E-06	-	2.21E-07	8.56E-06	-	1.10E-06	1.71E-05	-	2.21E-06	2.57E-05	-	3.31E-06	3.43E-05	-	4.42E-06
76	C148 /II/22/23	1.47E-06	-	2.72E-07	7.34E-06	-	1.36E-06	1.47E-05	-	2.72E-06	2.20E-05	-	4.07E-06	2.94E-05	-	5.43E-06
77	C149 /II/22/23	-	-	3.16E-07	-	-	1.58E-06	-	-	3.16E-06	-	-	4.74E-06	-	-	6.31E-06
78	C15 /II/22/23	3.70E-06	-	3.19E-07	1.85E-05	-	1.59E-06	3.70E-05	-	3.19E-06	5.55E-05	-	4.78E-06	7.40E-05	-	6.37E-06
79	C86 /II/21/22	1.32E-07	-	3.34E-07	6.61E-07	-	1.67E-06	1.32E-06	-	3.34E-06	1.98E-06	-	5.02E-06	2.64E-06	-	6.69E-06
80	C87 /II/21/22	-	-	2.38E-07	-	-	1.19E-06	-	-	2.38E-06	-	-	3.57E-06	-	-	4.76E-06
81	C88 /II/21/22	8.25E-08	-	3.29E-07	4.12E-07	-	1.64E-06	8.25E-07	-	3.29E-06	1.24E-06	-	4.93E-06	1.65E-06	-	6.57E-06
82	C89 /II/21/22	9.02E-08	6.34E-07	1.72E-07	4.51E-07	3.17E-06	8.60E-07	9.02E-07	6.34E-06	1.72E-06	1.35E-06	9.51E-06	2.58E-06	1.80E-06	1.27E-05	3.44E-06
83	C90 /II/21/22	8.99E-08	9.36E-07	3.45E-07	4.49E-07	4.68E-06	1.72E-06	8.99E-07	9.36E-06	3.45E-06	1.35E-06	1.40E-05	5.17E-06	1.80E-06	1.87E-05	6.90E-06
84	C91 /II/21/22	-	-	3.16E-07	-	-	1.58E-06	-	-	3.16E-06	-	-	4.74E-06	-	-	6.32E-06
85	C92 /II/21/22	1.06E-07	-	3.23E-07	5.29E-07	-	1.61E-06	1.06E-06	-	3.23E-06	1.59E-06	-	4.84E-06	2.11E-06	-	6.45E-06
86	C93 /II/21/22	6.66E-08	-	1.75E-07	3.33E-07	-	8.73E-07	6.66E-07	-	1.75E-06	9.99E-07	-	2.62E-06	1.33E-06	-	3.49E-06
87	C94 /II/21/22	7.76E-08	6.40E-07	1.63E-07	3.88E-07	3.20E-06	8.17E-07	7.76E-07	6.40E-06	1.63E-06	1.16E-06	9.60E-06	2.45E-06	1.55E-06	1.28E-05	3.27E-06
88	C95 /II/21/22	6.98E-08	-	1.39E-07	3.49E-07	-	6.97E-07	6.98E-07	-	1.39E-06	1.05E-06	-	2.09E-06	1.40E-06	-	2.79E-06
89	C96 /II/21/22	-	-	2.09E-07	-	-	1.04E-06	-	-	2.09E-06	-	-	3.13E-06	-	-	4.18E-06
90	C97 /II/21/22	-	6.73E-07	2.26E-07	-	3.36E-06	1.13E-06	-	6.73E-06	2.26E-06	-	1.01E-05	3.39E-06	-	1.35E-05	4.52E-06
91	C98 /II/21/22	-	-	2.16E-07	-	-	1.08E-06	-	-	2.16E-06	-	-	3.24E-06	-	-	4.32E-06
92	C99 /II/21/22	9.10E-08	6.29E-07	3.52E-07	4.55E-07	3.14E-06	1.76E-06	9.10E-07	6.29E-06	3.52E-06	1.36E-06	9.43E-06	5.28E-06	1.82E-06	1.26E-05	7.04E-06
93	C101 /II/21/22	1.00E-07	7.06E-07	3.57E-07	5.02E-07	3.53E-06	1.79E-06	1.00E-06	7.06E-06	3.57E-06	1.50E-06	1.06E-05	5.36E-06	2.01E-06	1.41E-05	7.14E-06

94	C16 /II/21/22	-	8.08E-07	2.62E-07	-	4.04E-06	1.31E-06	-	8.08E-06	2.62E-06	-	1.21E-05	3.93E-06	-	1.62E-05	5.24E-06
95	C17 /II/21/22	1.41E-07	-	4.57E-07	7.03E-07	-	2.29E-06	1.41E-06	-	4.57E-06	2.11E-06	-	6.86E-06	2.81E-06	-	9.15E-06
96	C18 /II/21/22	5.64E-08	5.58E-06	3.41E-07	2.82E-07	2.79E-05	1.70E-06	5.64E-07	5.58E-05	3.41E-06	8.47E-07	8.37E-05	5.11E-06	1.13E-06	1.12E-04	6.81E-06
97	C21 /II/21/22	-	1.06E-06	4.34E-07	-	5.28E-06	2.17E-06	-	1.06E-05	4.34E-06	-	1.58E-05	6.51E-06	-	2.11E-05	8.68E-06
98	C39 /II/21/22	-	1.15E-06	3.04E-07	-	5.75E-06	1.52E-06	-	1.15E-05	3.04E-06	-	1.73E-05	4.56E-06	-	2.30E-05	6.08E-06
99	C40 /II/21/22	-	1.04E-06	3.83E-07	-	5.19E-06	1.91E-06	-	1.04E-05	3.83E-06	-	1.56E-05	5.74E-06	-	2.07E-05	7.66E-06
100	C41 /II/21/22	1.19E-07	9.25E-07	3.08E-07	5.96E-07	4.62E-06	1.54E-06	1.19E-06	9.25E-06	3.08E-06	1.79E-06	1.39E-05	4.62E-06	2.38E-06	1.85E-05	6.16E-06
101	C43 /II/21/22	6.04E-08	2.32E-06	3.94E-07	3.02E-07	1.16E-05	1.97E-06	6.04E-07	2.32E-05	3.94E-06	9.06E-07	3.48E-05	5.91E-06	1.21E-06	4.64E-05	7.88E-06
102	C44 /II/21/22	-	8.99E-07	4.19E-07	-	4.50E-06	2.10E-06	-	8.99E-06	4.19E-06	-	1.35E-05	6.29E-06	-	1.80E-05	8.39E-06
103	C46 /II/21/22	-	1.52E-06	3.66E-07	-	7.62E-06	1.83E-06	-	1.52E-05	3.66E-06	-	2.29E-05	5.49E-06	-	3.05E-05	7.32E-06
104	C314 /II/17/18	4.75E-05	-	3.10E-06	2.37E-04	-	1.55E-05	4.75E-04	-	3.10E-05	7.12E-04	-	4.64E-05	9.49E-04	-	6.19E-05
105	C315 /II/17/18	5.18E-06	-	1.81E-06	2.59E-05	-	9.05E-06	5.18E-05	-	1.81E-05	7.78E-05	-	2.71E-05	1.04E-04	-	3.62E-05
106	C319 /II/17/18	2.92E-06	-	2.02E-07	1.46E-05	-	1.01E-06	2.92E-05	-	2.02E-06	4.38E-05	-	3.03E-06	5.84E-05	-	4.05E-06
107	C320 /II/17/18	1.66E-06	-	1.03E-06	8.31E-06	-	5.17E-06	1.66E-05	-	1.03E-05	2.49E-05	-	1.55E-05	3.32E-05	-	2.07E-05
108	C321 /II/17/18	5.69E-06	-	1.04E-06	2.84E-05	-	5.18E-06	5.69E-05	-	1.04E-05	8.53E-05	-	1.56E-05	1.14E-04	-	2.07E-05
109	C322 /II/17/18	6.04E-06	-	3.90E-07	3.02E-05	-	1.95E-06	6.04E-05	-	3.90E-06	9.05E-05	-	5.85E-06	1.21E-04	-	7.80E-06
110	C323 /II/17/18	-	1.87E-06	1.05E-07	-	9.35E-06	5.24E-07	-	1.87E-05	1.05E-06	-	2.81E-05	1.57E-06	-	3.74E-05	2.10E-06
111	C324 /II/17/18	4.95E-06	-	1.08E-06	2.47E-05	-	5.40E-06	4.95E-05	-	1.08E-05	7.42E-05	-	1.62E-05	9.89E-05	-	2.16E-05

112	C35 /I/23/24	6.69E-07	-	3.54E-07	3.35E-06	-	1.77E-06	6.69E-06	-	3.54E-06	1.00E-05	-	5.31E-06	1.34E-05	-	7.08E-06
113	C168 /I/22/23	-	-	1.68E-07	-	-	8.39E-07	-	-	1.68E-06	-	-	2.52E-06	-	-	3.35E-06
114	C175 /I/22/23	1.01E-07	-	2.42E-07	5.05E-07	-	1.21E-06	1.01E-06	-	2.42E-06	1.51E-06	-	3.63E-06	2.02E-06	-	4.84E-06
115	C100 /II/21/22	6.67E-08	-	5.41E-07	3.33E-07	-	2.71E-06	6.67E-07	-	5.41E-06	1.00E-06	-	8.12E-06	1.33E-06	-	1.08E-05
116	C20 /II/21/22	7.74E-08	1.49E-06	3.92E-07	3.87E-07	7.45E-06	1.96E-06	7.74E-07	1.49E-05	3.92E-06	1.16E-06	2.23E-05	5.88E-06	1.55E-06	2.98E-05	7.85E-06
117	C316 /II/17/18	3.06E-06	-	3.47E-07	1.53E-05	-	1.73E-06	3.06E-05	-	3.47E-06	4.58E-05	-	5.20E-06	6.11E-05	-	6.94E-06
118	C317 /II/17/18	4.06E-06	-	2.24E-07	2.03E-05	-	1.12E-06	4.06E-05	-	2.24E-06	6.08E-05	-	3.36E-06	8.11E-05	-	4.48E-06
119	C326 /II/17/18	2.72E-06	1.98E-06	1.46E-06	1.36E-05	9.90E-06	7.31E-06	2.72E-05	1.98E-05	1.46E-05	4.09E-05	2.97E-05	2.19E-05	5.45E-05	3.96E-05	2.93E-05

Table S10. Lifetime Cancer Risk (LCR) for ingestion exposure to lead, nickel, and cadmium contained in the cigarettes tested.

No.	Sample code	1 cigarette per day			5 cigarettes per day			10 cigarettes per day			15 cigarettes per day			20 cigarettes per day		
		**Pb**	**Ni**	**Cd**	**Pb**	**Ni**	**Cd**	**Pb**	**Ni**	**Cd**	**Pb**	**Ni**	**Cd**	**Pb**	**Ni**	**Cd**
1	C20 /I/23/24	8.25E-05	-	7.86E-07	**4.13E-04**	-	3.93E-06	**8.25E-04**	-	7.86E-06	**1.24E-03**	-	1.18E-05	**1.65E-03**	-	1.57E-05
2	C21 /I/23/24	2.77E-05	-	5.97E-07	**1.39E-04**	-	2.99E-06	**2.77E-04**	-	5.97E-06	**4.16E-04**	-	8.96E-06	**5.55E-04**	-	1.19E-05
3	C22 /I/23/24	6.57E-05	-	7.44E-07	**3.29E-04**	-	3.72E-06	**6.57E-04**	-	7.44E-06	**9.86E-04**	-	1.12E-05	**1.31E-03**	-	1.49E-05
4	C23 /I/23/24	6.01E-05	-	8.75E-07	**3.00E-04**	-	4.38E-06	**6.01E-04**	-	8.75E-06	**9.01E-04**	-	1.31E-05	**1.20E-03**	-	1.75E-05
5	C24 /I/23/24	6.98E-06	-	7.02E-07	3.49E-05	-	3.51E-06	6.98E-05	-	7.02E-06	**1.05E-04**	-	1.05E-05	**1.40E-04**	-	1.40E-05
6	C25 /I/23/24	5.49E-05	-	6.55E-07	**2.74E-04**	-	3.27E-06	**5.49E-04**	-	6.55E-06	**8.23E-04**	-	9.82E-06	**1.10E-03**	-	1.31E-05
7	C26 /I/23/24	5.58E-05	-	1.38E-06	**2.79E-04**	-	6.88E-06	**5.58E-04**	-	1.38E-05	**8.37E-04**	-	2.06E-05	**1.12E-03**	-	2.75E-05
8	C27 /I/23/24	3.25E-05	-	1.05E-06	**1.63E-04**	-	5.26E-06	**3.25E-04**	-	1.05E-05	**4.88E-04**	-	1.58E-05	**6.50E-04**	-	2.10E-05
9	C28 /I/23/24	4.52E-05	-	7.06E-07	**2.26E-04**	-	3.53E-06	**4.52E-04**	-	7.06E-06	**6.77E-04**	-	1.06E-05	**9.03E-04**	-	1.41E-05
10	C29 /I/23/24	2.70E-05	-	1.03E-06	**1.35E-04**	-	5.17E-06	**2.70E-04**	-	1.03E-05	**4.06E-04**	-	1.55E-05	**5.41E-04**	-	2.07E-05
11	C30 /I/23/24	-	-	8.86E-07	-	-	4.43E-06	-	-	8.86E-06	-	-	1.33E-05	**-**	-	1.77E-05
12	C31 /I/23/24	5.97E-05	-	6.73E-07	**2.99E-04**	-	3.37E-06	**5.97E-04**	-	6.73E-06	**8.96E-04**	-	1.01E-05	**1.19E-03**	-	1.35E-05
13	C32 /I/23/24	6.01E-05	-	9.83E-07	**3.00E-04**	-	4.91E-06	**6.01E-04**	-	9.83E-06	**9.01E-04**	-	1.47E-05	**1.20E-03**	-	1.97E-05
14	C33 /I/23/24	4.86E-05	-	7.59E-07	**2.43E-04**	-	3.80E-06	**4.86E-04**	-	7.59E-06	**7.29E-04**	-	1.14E-05	**9.72E-04**	-	1.52E-05
15	C34 /I/23/24	3.39E-05	-	8.57E-07	**1.70E-04**	-	4.28E-06	**3.39E-04**	-	8.57E-06	**5.09E-04**	-	1.29E-05	**6.78E-04**	-	1.71E-05
16	C36 /I/23/24	6.10E-05	-	9.22E-07	**3.05E-04**	-	4.61E-06	**6.10E-04**	-	9.22E-06	**9.15E-04**	-	1.38E-05	**1.22E-03**	-	1.84E-05
17	C37 /I/23/24	**1.19E-04**	-	1.04E-06	**5.97E-04**	-	5.22E-06	**1.19E-03**	-	1.04E-05	**1.79E-03**	-	1.57E-05	**2.39E-03**	-	2.09E-05

18	C38 /I/23/24	7.61E-05	-	8.98E-07	**3.80E-04**	-	4.49E-06	**7.61E-04**	-	8.98E-06	**1.14E-03**	-	1.35E-05	**1.52E-03**	-	1.80E-05
19	C39 /I/23/24	4.30E-05	-	6.17E-07	**2.15E-04**	-	3.08E-06	**4.30E-04**	-	6.17E-06	**6.44E-04**	-	9.25E-06	**8.59E-04**	-	1.23E-05
20	C128 /I/22/23	8.38E-06	5.01E-07	6.94E-07	4.19E-05	2.51E-06	3.47E-06	8.38E-05	2.51E-06	6.94E-06	**1.26E-04**	1.47E-06	1.04E-05	**1.68E-04**	1.00E-05	1.39E-05
21	C129 /I/22/23	-	4.95E-07	4.37E-07	-	2.48E-06	2.19E-06	-	2.48E-06	4.37E-06	-	1.46E-06	6.56E-06	-	9.90E-06	8.75E-06
22	C130 /I/22/23	1.47E-05	4.88E-07	4.78E-07	7.34E-05	2.44E-06	2.39E-06	**1.47E-04**	2.44E-06	4.78E-06	**2.20E-04**	1.44E-06	7.17E-06	**2.94E-04**	9.77E-06	9.56E-06
23	C131 /I/22/23	6.73E-06	9.76E-07	3.63E-07	3.36E-05	4.88E-06	1.81E-06	6.73E-05	4.88E-06	3.63E-06	**1.01E-04**	2.87E-06	5.44E-06	**1.35E-04**	1.95E-05	7.26E-06
24	C132 /I/22/23	1.62E-05	2.03E-06	3.53E-06	8.08E-05	1.01E-05	1.76E-05	**1.62E-04**	1.01E-05	3.53E-05	**2.42E-04**	5.97E-06	5.29E-05	**3.23E-04**	4.06E-05	7.05E-05
25	C133 /I/22/23	-	1.70E-06	4.38E-07	-	8.52E-06	2.19E-06	-	8.52E-06	4.38E-06	-	5.01E-06	6.57E-06	-	3.41E-05	8.76E-06
26	C134 /I/22/23	-	2.75E-07	1.02E-06	-	1.38E-06	5.11E-06	-	1.38E-06	1.02E-05	-	8.09E-07	1.53E-05	-	5.50E-06	2.04E-05
27	C135 /I/22/23	-	1.26E-06	6.03E-07	-	6.31E-06	3.02E-06	-	6.31E-06	6.03E-06	-	3.71E-06	9.05E-06	-	2.53E-05	1.21E-05
28	C136 /I/22/23	-	2.24E-06	1.03E-06	-	1.12E-05	5.15E-06	-	1.12E-05	1.03E-05	-	6.60E-06	1.55E-05	-	4.49E-05	2.06E-05
29	C137 /I/22/23	1.08E-05	2.49E-06	4.15E-07	5.38E-05	1.24E-05	2.07E-06	**1.08E-04**	1.24E-05	4.15E-06	**1.61E-04**	7.31E-06	6.22E-06	**2.15E-04**	4.97E-05	8.30E-06
30	C138 /I/22/23	-	1.28E-06	5.13E-07	-	6.42E-06	2.57E-06	-	6.42E-06	5.13E-06	-	3.77E-06	7.70E-06	-	2.57E-05	1.03E-05
31	C169 /I/22/23	1.46E-05	2.66E-07	1.31E-06	7.31E-05	1.33E-06	6.55E-06	**1.46E-04**	1.33E-06	1.31E-05	**2.19E-04**	7.83E-07	1.97E-05	**2.92E-04**	5.32E-06	2.62E-05
32	C170 /I/22/23	-	5.29E-07	5.42E-07	-	2.64E-06	2.71E-06	-	2.64E-06	5.42E-06	-	1.55E-06	8.12E-06	-	1.06E-05	1.08E-05
33	C171 /I/22/23	-	4.21E-07	6.66E-07	-	2.10E-06	3.33E-06	-	2.10E-06	6.66E-06	-	1.24E-06	1.00E-05	-	8.42E-06	1.33E-05
34	C172 /I/22/23	-	2.67E-07	5.35E-07	-	1.33E-06	2.67E-06	-	1.33E-06	5.35E-06	-	7.85E-07	8.02E-06	-	5.34E-06	1.07E-05
35	C173 /I/22/23	-	5.16E-07	6.11E-07	-	2.58E-06	3.05E-06	-	2.58E-06	6.11E-06	-	1.52E-06	9.16E-06	-	1.03E-05	1.22E-05
36	C174 /I/22/23	-	5.32E-07	8.76E-07	-	2.66E-06	4.38E-06	-	2.66E-06	8.76E-06	-	1.56E-06	1.31E-05	-	1.06E-05	1.75E-05

37	C176 /I/22/23	9.54E-06	5.16E-07	7.97E-07	4.77E-05	2.58E-06	3.98E-06	9.54E-05	2.58E-06	7.97E-06	**1.43E-04**	1.52E-06	1.20E-05	**1.91E-04**	1.03E-05	1.59E-05
38	C177 /I/22/23	-	3.63E-07	8.01E-07	-	1.82E-06	4.01E-06	-	1.82E-06	8.01E-06	-	1.07E-06	1.20E-05	-	7.27E-06	1.60E-05
39	C178 /I/22/23	1.27E-05	4.14E-07	9.89E-07	6.36E-05	2.07E-06	4.95E-06	**1.27E-04**	2.07E-06	9.89E-06	**1.91E-04**	1.22E-06	1.48E-05	**2.54E-04**	8.27E-06	1.98E-05
40	C179 /I/22/23	-	2.40E-07	7.13E-07	-	1.20E-06	3.56E-06	-	1.20E-06	7.13E-06	-	7.07E-07	1.07E-05	-	4.81E-06	1.43E-05
41	C180 /I/22/23	-	6.28E-07	6.82E-07	-	3.14E-06	3.41E-06	-	3.14E-06	6.82E-06	-	1.85E-06	1.02E-05	-	1.26E-05	1.36E-05
42	C181 /I/22/23	7.05E-06	5.40E-07	4.54E-07	3.52E-05	2.70E-06	2.27E-06	7.05E-05	2.70E-06	4.54E-06	**1.06E-04**	1.59E-06	6.82E-06	**1.41E-04**	1.08E-05	9.09E-06
43	C182 /I/22/23	1.23E-05	4.66E-07	5.87E-07	6.13E-05	2.33E-06	2.94E-06	**1.23E-04**	2.33E-06	5.87E-06	**1.84E-04**	1.37E-06	8.81E-06	**2.45E-04**	9.31E-06	1.17E-05
44	C183 /I/22/23	1.31E-05	2.46E-07	6.81E-07	6.56E-05	1.23E-06	3.41E-06	**1.31E-04**	1.23E-06	6.81E-06	**1.97E-04**	7.23E-07	1.02E-05	**2.62E-04**	4.92E-06	1.36E-05
45	C184 /I/22/23	1.82E-05	3.26E-07	3.85E-07	9.08E-05	1.63E-06	1.92E-06	**1.82E-04**	1.63E-06	3.85E-06	**2.72E-04**	9.59E-07	5.77E-06	**3.63E-04**	6.52E-06	7.69E-06
46	C185 /I/22/23	6.91E-06	2.53E-07	2.95E-07	3.45E-05	1.26E-06	1.47E-06	6.91E-05	1.26E-06	2.95E-06	**1.04E-04**	7.43E-07	4.42E-06	**1.38E-04**	5.05E-06	5.90E-06
47	C186 /I/22/23	-	4.72E-07	3.93E-07	-	2.36E-06	1.97E-06	-	2.36E-06	3.93E-06	-	1.39E-06	5.90E-06	-	9.43E-06	7.86E-06
48	C187 /I/22/23	-	4.12E-07	5.20E-07	-	2.06E-06	2.60E-06	-	2.06E-06	5.20E-06	-	1.21E-06	7.80E-06	-	8.24E-06	1.04E-05
49	C188 /I/22/23	2.65E-05	5.48E-07	3.34E-07	**1.32E-04**	2.74E-06	1.67E-06	**2.65E-04**	2.74E-06	3.34E-06	**3.97E-04**	1.61E-06	5.01E-06	**5.29E-04**	1.10E-05	6.68E-06
50	C64 /II/22/23	-	4.89E-07	7.95E-07	-	2.44E-06	3.98E-06	-	2.44E-06	7.95E-06	-	1.44E-06	1.19E-05	-	9.78E-06	1.59E-05
51	C65 /II/22/23	-	2.23E-06	7.20E-07	-	1.12E-05	3.60E-06	-	1.12E-05	7.20E-06	-	6.56E-06	1.08E-05	-	4.46E-05	1.44E-05
52	C66 /II/22/23	-	2.83E-07	8.20E-07	-	1.42E-06	4.10E-06	-	1.42E-06	8.20E-06	-	8.33E-07	1.23E-05	-	5.67E-06	1.64E-05
53	C67 /II/22/23	-	5.99E-07	7.05E-07	-	3.00E-06	3.52E-06	-	3.00E-06	7.05E-06	-	1.76E-06	1.06E-05	-	1.20E-05	1.41E-05
54	C68 /II/22/23	-	3.11E-07	6.02E-07	-	1.55E-06	3.01E-06	-	1.55E-06	6.02E-06	-	9.14E-07	9.03E-06	-	6.22E-06	1.20E-05
55	C69 /II/22/23	-	6.69E-07	5.81E-07	-	3.35E-06	2.91E-06	-	3.35E-06	5.81E-06	-	1.97E-06	8.72E-06	-	1.34E-05	1.16E-05

56	C70 /II/22/23	-	2.24E-07	6.68E-07	-	1.12E-06	3.34E-06	-	1.12E-06	6.68E-06	-	6.60E-07	1.00E-05	-	4.49E-06	1.34E-05
57	C128 /II/22/23	**6.94E-02**	2.56E-07	6.02E-07	**3.47E-01**	1.28E-06	3.01E-06	**6.94E-01**	1.28E-06	6.02E-06	**1.04E+00**	7.54E-07	9.03E-06	**1.39E+00**	5.13E-06	1.20E-05
58	C129 /II/22/23	**3.59E-02**	-	6.63E-07	**1.80E-01**	-	3.31E-06	**3.59E-01**	-	6.63E-06	**5.39E-01**	-	9.94E-06	**7.19E-01**	-	1.33E-05
59	C130 /II/22/23	**1.77E-02**	-	6.14E-07	**8.85E-02**	-	3.07E-06	**1.77E-01**	-	6.14E-06	**2.66E-01**	-	9.22E-06	**3.54E-01**	-	1.23E-05
60	C131 /II/22/23	**5.05E-03**	2.30E-07	9.14E-07	**2.52E-02**	1.15E-06	4.57E-06	**5.05E-02**	1.15E-06	9.14E-06	**7.57E-02**	6.77E-07	1.37E-05	**1.01E-01**	4.60E-06	1.83E-05
61	C133 /II/22/23	**2.90E-03**	-	6.15E-07	**1.45E-02**	-	3.08E-06	**2.90E-02**	-	6.15E-06	**4.35E-02**	-	9.23E-06	**5.81E-02**	-	1.23E-05
62	C134 /II/22/23	**5.87E-03**	-	6.93E-07	**2.93E-02**	-	3.47E-06	**5.87E-02**	-	6.93E-06	**8.80E-02**	-	1.04E-05	**1.17E-01**	-	1.39E-05
63	C135 /II/22/23	**2.50E-03**	2.55E-07	5.39E-07	**1.25E-02**	1.28E-06	2.69E-06	**2.50E-02**	1.28E-06	5.39E-06	**3.76E-02**	7.50E-07	8.08E-06	**5.01E-02**	5.10E-06	1.08E-05
64	C136 /II/22/23	**1.56E-03**	1.27E-06	9.66E-07	**7.79E-03**	6.37E-06	4.83E-06	**1.56E-02**	6.37E-06	9.66E-06	**2.34E-02**	3.75E-06	1.45E-05	**3.11E-02**	2.55E-05	1.93E-05
65	C137 /II/22/23	**5.41E-04**	9.80E-07	4.58E-07	**2.70E-03**	4.90E-06	2.29E-06	**5.41E-03**	4.90E-06	4.58E-06	**8.11E-03**	2.88E-06	6.86E-06	**1.08E-02**	1.96E-05	9.15E-06
66	C138 /II/22/23	**6.57E-04**	1.49E-06	9.66E-07	**3.29E-03**	7.44E-06	4.83E-06	**6.57E-03**	7.44E-06	9.66E-06	**9.86E-03**	4.38E-06	1.45E-05	**1.31E-02**	2.98E-05	1.93E-05
67	C139 /II/22/23	**4.03E-04**	-	5.67E-07	**2.01E-03**	-	2.83E-06	**4.03E-03**	-	5.67E-06	**6.04E-03**	-	8.50E-06	**8.06E-03**	-	1.13E-05
68	C140 /II/22/23	**3.13E-04**	-	5.03E-07	**1.56E-03**	-	2.51E-06	**3.13E-03**	-	5.03E-06	**4.69E-03**	-	7.54E-06	**6.26E-03**	-	1.01E-05
69	C141 /II/22/23	**3.39E-04**	-	4.92E-07	**1.70E-03**	-	2.46E-06	**3.39E-03**	-	4.92E-06	**5.09E-03**	-	7.38E-06	**6.78E-03**	-	9.84E-06
70	C142 /II/22/23	**7.98E-04**	-	6.14E-07	**3.99E-03**	-	3.07E-06	**7.98E-03**	-	6.14E-06	**1.20E-02**	-	9.21E-06	**1.60E-02**	-	1.23E-05
71	C143 /II/22/23	-	-	7.70E-07	-	-	3.85E-06	-	-	7.70E-06	-	-	1.15E-05	-	-	1.54E-05
72	C144 /II/22/23	**1.02E-03**	-	6.10E-07	**5.10E-03**	-	3.05E-06	**1.02E-02**	-	6.10E-06	**1.53E-02**	-	9.14E-06	**2.04E-02**	-	1.22E-05
73	C145 /II/22/23	**2.83E-04**	-	1.00E-06	**1.42E-03**	-	5.02E-06	**2.83E-03**	-	1.00E-05	**4.25E-03**	-	1.51E-05	**5.67E-03**	-	2.01E-05
74	C146 /II/22/23	**6.15E-04**	3.55E-07	8.64E-07	**3.07E-03**	1.77E-06	4.32E-06	**6.15E-03**	1.77E-06	8.64E-06	**9.22E-03**	1.04E-06	1.30E-05	**1.23E-02**	7.10E-06	1.73E-05

75	C147 /II/22/23	**2.01E-04**	-	5.81E-07	**1.01E-03**	-	2.91E-06	**2.01E-03**	-	5.81E-06	**3.02E-03**	-	8.72E-06	**4.03E-03**	-	1.16E-05
76	C148 /II/22/23	**1.73E-04**	-	7.15E-07	**8.64E-04**	-	3.57E-06	**1.73E-03**	-	7.15E-06	**2.59E-03**	-	1.07E-05	**3.46E-03**	-	1.43E-05
77	C149 /II/22/23	-	-	8.31E-07	-	-	4.15E-06	-	-	8.31E-06	-	-	1.25E-05	-	-	1.66E-05
78	C15 /II/22/23	**4.35E-04**	-	8.38E-07	**2.18E-03**	-	4.19E-06	**4.35E-03**	-	8.38E-06	**6.53E-03**	-	1.26E-05	**8.70E-03**	-	1.68E-05
79	C86 /II/21/22	1.56E-05	-	8.80E-07	7.78E-05	-	4.40E-06	**1.56E-04**	-	8.80E-06	**2.33E-04**	-	1.32E-05	**3.11E-04**	-	1.76E-05
80	C87 /II/21/22	-	-	6.26E-07	-	-	3.13E-06	-	-	6.26E-06	-	-	9.39E-06	-	-	1.25E-05
81	C88 /II/21/22	9.70E-06	-	8.65E-07	4.85E-05	-	4.32E-06	9.70E-05	-	8.65E-06	**1.46E-04**	-	1.30E-05	**1.94E-04**	-	1.73E-05
82	C89 /II/21/22	1.06E-05	3.73E-07	4.53E-07	5.31E-05	1.86E-06	2.26E-06	**1.06E-04**	1.86E-06	4.53E-06	**1.59E-04**	1.10E-06	6.79E-06	**2.12E-04**	7.46E-06	9.05E-06
83	C90 /II/21/22	1.06E-05	5.50E-07	9.07E-07	5.29E-05	2.75E-06	4.54E-06	**1.06E-04**	2.75E-06	9.07E-06	**1.59E-04**	1.62E-06	1.36E-05	**2.12E-04**	1.10E-05	1.81E-05
84	C91 /II/21/22	-	-	8.31E-07	-	-	4.16E-06	-	-	8.31E-06	-	-	1.25E-05	-	-	1.66E-05
85	C92 /II/21/22	1.24E-05	-	8.49E-07	6.22E-05	-	4.24E-06	**1.24E-04**	-	8.49E-06	**1.87E-04**	-	1.27E-05	**2.49E-04**	-	1.70E-05
86	C93 /II/21/22	7.84E-06	-	4.59E-07	3.92E-05	-	2.30E-06	7.84E-05	-	4.59E-06	**1.18E-04**	-	6.89E-06	**1.57E-04**	-	9.19E-06
87	C94 /II/21/22	9.12E-06	3.76E-07	4.30E-07	4.56E-05	1.88E-06	2.15E-06	9.12E-05	1.88E-06	4.30E-06	**1.37E-04**	1.11E-06	6.45E-06	**1.82E-04**	7.53E-06	8.60E-06
88	C95 /II/21/22	8.22E-06	-	3.67E-07	4.11E-05	-	1.83E-06	8.22E-05	-	3.67E-06	**1.23E-04**	-	5.50E-06	**1.64E-04**	-	7.34E-06
89	C96 /II/21/22	-	-	5.50E-07	-	-	2.75E-06	-	-	5.50E-06	-	-	8.25E-06	-	-	1.10E-05
90	C97 /II/21/22	-	3.96E-07	5.95E-07	-	1.98E-06	2.97E-06	-	1.98E-06	5.95E-06	-	1.16E-06	8.92E-06	-	7.91E-06	1.19E-05
91	C98 /II/21/22	-	-	5.68E-07	-	-	2.84E-06	-	-	5.68E-06	-	-	8.52E-06	-	-	1.14E-05
92	C99 /II/21/22	1.07E-05	3.70E-07	9.26E-07	5.35E-05	1.85E-06	4.63E-06	**1.07E-04**	1.85E-06	9.26E-06	**1.61E-04**	1.09E-06	1.39E-05	**2.14E-04**	7.40E-06	1.85E-05
93	C101 /II/21/22	1.18E-05	4.15E-07	9.40E-07	5.90E-05	2.08E-06	4.70E-06	**1.18E-04**	2.08E-06	9.40E-06	**1.77E-04**	1.22E-06	1.41E-05	**2.36E-04**	8.30E-06	1.88E-05

94	C16 /II/21/22	-	4.75E-07	6.89E-07	-	2.38E-06	3.45E-06	-	2.38E-06	6.89E-06	-	1.40E-06	1.03E-05	-	9.50E-06	1.38E-05
95	C17 /II/21/22	1.65E-05	2.36E-07	1.20E-06	8.27E-05	1.18E-06	6.02E-06	**1.65E-04**	1.18E-06	1.20E-05	**2.48E-04**	6.94E-07	1.81E-05	**3.31E-04**	4.72E-06	2.41E-05
96	C18 /II/21/22	6.64E-06	3.28E-06	8.97E-07	3.32E-05	1.64E-05	4.48E-06	6.64E-05	1.64E-05	8.97E-06	9.96E-05	9.65E-06	1.35E-05	**1.33E-04**	6.56E-05	1.79E-05
97	C21 /II/21/22	-	6.21E-07	1.14E-06	-	3.11E-06	5.71E-06	-	3.11E-06	1.14E-05	-	1.83E-06	1.71E-05	-	1.24E-05	2.28E-05
98	C39 /II/21/22	-	6.77E-07	7.99E-07	-	3.38E-06	4.00E-06	-	3.38E-06	7.99E-06	-	1.99E-06	1.20E-05	-	1.35E-05	1.60E-05
99	C40 /II/21/22	-	6.10E-07	1.01E-06	-	3.05E-06	5.04E-06	-	3.05E-06	1.01E-05	-	1.79E-06	1.51E-05	-	1.22E-05	2.02E-05
100	C41 /II/21/22	1.40E-05	5.44E-07	8.10E-07	7.01E-05	2.72E-06	4.05E-06	**1.40E-04**	2.72E-06	8.10E-06	**2.10E-04**	1.60E-06	1.21E-05	**2.81E-04**	1.09E-05	1.62E-05
101	C43 /II/21/22	7.10E-06	1.36E-06	1.04E-06	3.55E-05	6.82E-06	5.19E-06	7.10E-05	6.82E-06	1.04E-05	**1.07E-04**	4.01E-06	1.56E-05	**1.42E-04**	2.73E-05	2.07E-05
102	C44 /II/21/22	-	5.29E-07	1.10E-06	-	2.65E-06	5.52E-06	-	2.65E-06	1.10E-05	-	1.56E-06	1.66E-05	-	1.06E-05	2.21E-05
103	C46 /II/21/22	-	8.96E-07	9.63E-07	-	4.48E-06	4.81E-06	-	4.48E-06	9.63E-06	-	2.64E-06	1.44E-05	-	1.79E-05	1.93E-05
104	C314 /II/17/18	**5.58E-03**	-	8.15E-06	**2.79E-02**	-	4.07E-05	**5.58E-02**	-	8.15E-05	**8.38E-02**	-	**1.22E-04**	**1.12E-01**	-	**1.63E-04**
105	C315 /II/17/18	**6.10E-04**	-	4.76E-06	**3.05E-03**	-	2.38E-05	**6.10E-03**	-	4.76E-05	**9.15E-03**	-	7.14E-05	**1.22E-02**	-	9.52E-05
106	C319 /II/17/18	**3.43E-04**	-	5.32E-07	**1.72E-03**	-	2.66E-06	**3.43E-03**	-	5.32E-06	**5.15E-03**	-	7.98E-06	**6.87E-03**	-	1.06E-05
107	C320 /II/17/18	**1.95E-04**	-	2.72E-06	**9.77E-04**	-	1.36E-05	**1.95E-03**	-	2.72E-05	**2.93E-03**	-	4.08E-05	**3.91E-03**	-	5.44E-05
108	C321 /II/17/18	**6.69E-04**	-	2.73E-06	**3.35E-03**	-	1.36E-05	**6.69E-03**	-	2.73E-05	**1.00E-02**	-	4.09E-05	**1.34E-02**	-	5.46E-05
109	C322 /II/17/18	**7.10E-04**	-	1.03E-06	**3.55E-03**	-	5.13E-06	**7.10E-03**	-	1.03E-05	**1.07E-02**	-	1.54E-05	**1.42E-02**	-	2.05E-05
110	C323 /II/17/18	-	1.10E-06	2.76E-07	-	5.50E-06	1.38E-06	-	5.50E-06	2.76E-06	-	3.24E-06	4.14E-06	-	2.20E-05	5.51E-06
111	C324 /II/17/18	**5.82E-04**	-	2.84E-06	**2.91E-03**	-	1.42E-05	**5.82E-03**	-	2.84E-05	**8.73E-03**	-	4.26E-05	**1.16E-02**	-	5.68E-05

112	C35 /I/23/24	7.87E-05	-	9.32E-07	**3.94E-04**	-	4.66E-06	**7.87E-04**	-	9.32E-06	**1.18E-03**	-	1.40E-05	**1.57E-03**	-	1.86E-05
113	C168 /I/22/23	-	3.05E-07	4.41E-07	-	1.53E-06	2.21E-06	-	1.53E-06	4.41E-06	-	8.98E-07	6.62E-06	-	6.11E-06	8.83E-06
114	C175 /I/22/23	1.19E-05	2.83E-07	6.37E-07	5.94E-05	1.41E-06	3.19E-06	**1.19E-04**	1.41E-06	6.37E-06	**1.78E-04**	8.32E-07	9.56E-06	**2.37E-04**	5.65E-06	1.27E-05
115	C100 /II/21/22	7.85E-06	-	1.42E-06	3.92E-05	-	7.12E-06	7.85E-05	-	1.42E-05	**1.18E-04**	-	2.14E-05	**1.57E-04**	-	2.85E-05
116	C20 /II/21/22	9.11E-06	8.76E-07	1.03E-06	4.56E-05	4.38E-06	5.16E-06	9.11E-05	4.38E-06	1.03E-05	**1.37E-04**	2.58E-06	1.55E-05	**1.82E-04**	1.75E-05	2.06E-05
117	C316 /II/17/18	**3.60E-04**	-	9.13E-07	**1.80E-03**	-	4.56E-06	**3.60E-03**	-	9.13E-06	**5.39E-03**	-	1.37E-05	**7.19E-03**	-	1.83E-05
118	C317 /II/17/18	**4.77E-04**	-	5.89E-07	**2.39E-03**	-	2.95E-06	**4.77E-03**	-	5.89E-06	**7.16E-03**	-	8.84E-06	**9.54E-03**	-	1.18E-05
119	C326 /II/17/18	**3.20E-04**	1.17E-06	3.85E-06	**1.60E-03**	5.83E-06	1.92E-05	**3.20E-03**	5.83E-06	3.85E-05	**4.81E-03**	3.43E-06	5.77E-05	**6.41E-03**	2.33E-05	7.70E-05
